# General Consistency of Strong Discontinuity Kinematics in Embedded Finite Element Method (E-FEM) Formulations

**DOI:** 10.3390/ma14195640

**Published:** 2021-09-28

**Authors:** Alejandro Ortega Laborin, Emmanuel Roubin, Yann Malecot, Laurent Daudeville

**Affiliations:** Université Grenoble Alpes, CNRS, Grenoble INP, 3SR, 38000 Grenoble, France; emmanuel.roubin@univ-grenoble-alpes.fr (E.R.); yann.malecot@univ-grenoble-alpes.fr (Y.M.); laurent.daudeville@univ-grenoble-alpes.fr (L.D.)

**Keywords:** E-FEM modelling, embedded strong discontinuity, enhanced finite elements, incompatible modes, local fracture modelling, finite element enhancement functions

## Abstract

This paper performs an in-depth study of the theoretical basis behind the strong discontinuity methods to improve local fracture simulations using the Embedded Finite Element Method (E-FEM). The process starts from a review of the elemental enhancement functions found in current E-FEM literature, providing the reader a solid context of E-FEM fundamentals. A set of theoretical pathologies is then discussed, which prevent current frameworks from attaining full kinematic consistency and introduce unintended mesh dependencies. Based on this analysis, a new proposal of strong discontinuity enhancement functions is presented considering generalised fracture kinematics in a full tridimensional setting and a more robust definition of internal auxiliary functions. Element-level simulations are performed to compare the outputs within a group of selected E-FEM approaches, including the novel proposal. Simulations show that the new element formulation grants a wider level of basic kinematic coherence between the local fracture outputs and element kinematics, demonstrating an increase in robustness that might drive the usefulness of E-FEM techniques for fracture simulations to a higher level.

## 1. Introduction

One of the core features of the E-FEM modelling approach is the ability to simulate local material fractures by the introduction of strong discontinuity enhancement functions to elemental displacement fields. These functions are driven by internal variables representing the fracture kinematics in the normal and parallel directions to a given fracture plane.

This kind of internal variable fracture representation is based on the theory of incompatible modes [1], which allows contiguous elements in the same mesh to have internal kinematic states that do not respect rigorous continuity between them. This gives rise to efficient and transparent solution processes where element fracture kinematic variables can be calculated in a completely internal solution process. The update of all elemental contributions for a nonlinear global solution step can then be performed using a traditional FEM solver framework, requiring no modifications to it unless the framework is deliberately enriched with a non-local analysis feature such as, for example, the use of a crack path field method to ensure continuity between elements [2].

The E-FEM approach has been praised for its simple yet powerful capacity for the accurate modelling of local and global fracture processes of quasi-brittle materials over other methods such as X-FEM or the Partition of Unity [3]. The latter have a more robust and deeper definition of their kinematics, attacking directly the definition of nodal interpolation functions and their support. This allows a natural continuity across elements and a more enriched description of a fracture in general [4]. However, this additional complexity works as a double-edged sword, bringing more challenges to their numerical implementation.

Despite this fact, the E-FEM approach has not become as popular as other alternative FEM options such as X-FEM or gained much ground in commercial FEM packages such as ABAQUS or LS-DYNA [5,6,7]. While the simplicity of the E-FEM approach has been acknowledged many times, there are some numerical issues that prevent this formulation from gaining a consensual solid stance as a reliable and accurate structural numerical simulation approach for material fractures. These have been recognised by many authors working in the field: mesh dependencies, severe stress locking, unintended coupling between fracture displacement modes, among others [8,9,10].

**Figure 1:**
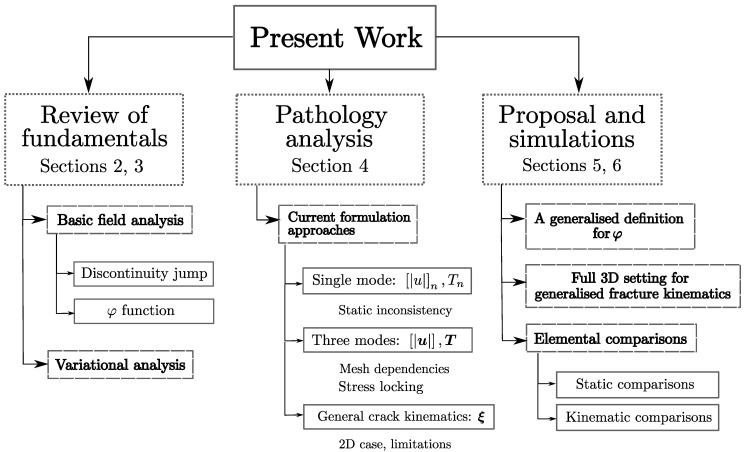
Auxiliary schema describing the general organisation of the theoretical works presented in this article.

The intent of this work is to make an analysis of how the basic definitions of strong discontinuity kinematics have been introduced so far on the E-FEM framework (Section 2 and Section 3) to identify the roots of its potential theoretical faults and related numerical issues (Section 4). Some authors provided their valuable insights on these challenges and proposed theoretical enhancements as a workaround [8,11,12,13]. This work attempts to consolidate these efforts to establish a basic understanding that allows for a new set of wiser E-FEM proposals in a general 3D format, avoiding current issues and bringing more robustness and modelling capabilities for the framework. Following the previous considerations, this paper proposes a new specific E-FEM strong discontinuity enhancement scheme (Section 5). Simulations at the element level are performed to compare the results to older enhancement proposals (Section 6), with encouraging conclusions (Section 7). For the reader’s convenience, Figure 1 describes the general layout and progression of the aforementioned discussions.

It is important to remark that the present work will focus on the branch of the E-FEM framework related to SKON (Statically and Kinematically Optimal Non-symmetric) formulations, which is the one that produces the most physically consistent models, both kinematically and statically [14,15,16,17]. The authors believe it is worth continuing the theoretical works in this line since its physical correctness sets a solid foundation for modelling further complex crack phenomena such as reclosure, local compression and explicit local friction, as well as the possibility to consider other kinds of discontinuities in a simultaneous fashion.

## 2. Analysis of Basic Internal Strong Discontinuity Kinematics

**Figure 2:**
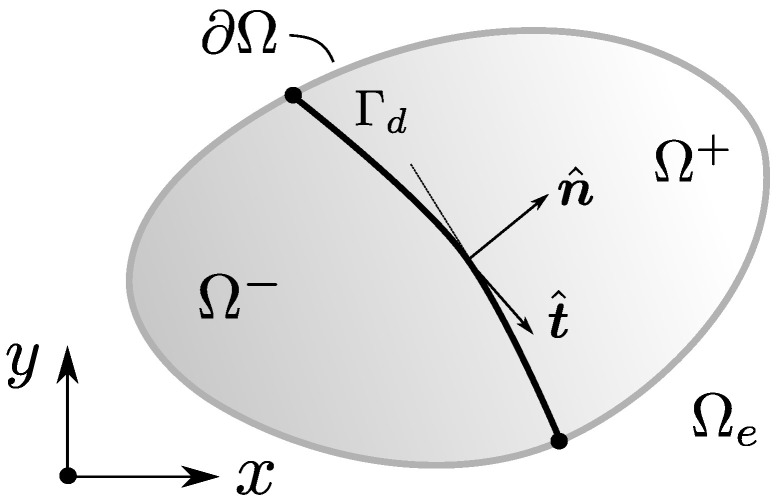
Basic schematic of an embedded strong discontinuity in 2D.

**Figure 3:**
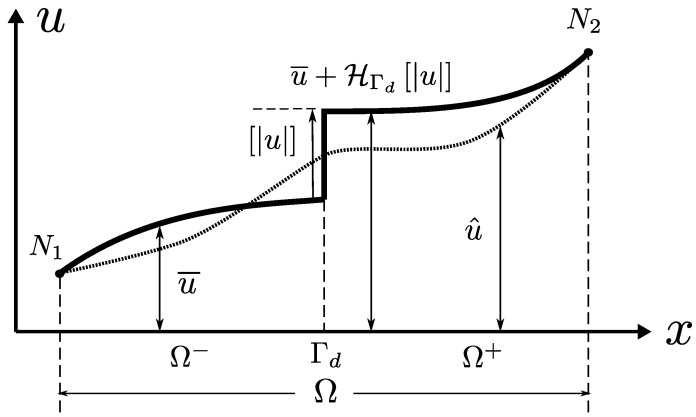
Example of the behaviour of the field *u* for a 1D element (conformed by nodes $N_{1}$ and $N_{2}$) with a constant displacement jump $\left[\left|u\right|\right]$, as well as a corresponding $\hat{u}$.

The starting point of every fracture representation within the E-FEM framework is to enrich the basic displacement field of a continuous body with the direct addition of a mathematical discontinuity function representing the displacement jump from one split region of the body to the other:

(1) $$u=\bar{u}+H_{\Gamma_{d}}\left[\left|u\right|\right],$$

where the theoretical displacement of the body u is set as the composition of a regular displacement $\bar{u}$ and a jump normally introduced by means of a Heaviside function having the location of the fault surface $\Gamma_{d}$ as its main trigger. An internal fracture kinematics vector $\left[\left|u\right|\right]$ is used to represent the general motion of one of the fractured regions with respect to the other. Figure 2 and Figure 3 are very common illustrations used by authors in the field to visualise the discontinuous enrichment for the fracture of a body split into two regions $\Omega^{+}$ and $\Omega^{-}$.

The origin, shape and orientation of the surface $\Gamma_{d}$ representing the fracture remain completely independent from this analysis, and will depend on the specific strain localisation criterion chosen for studying a given material. As the general philosophy of the E-FEM framework tends to aim for formulation simplicity, the most common fracture surface of choice is typically a plane or a line. This work assumes this simple geometry as the fracture surface from now on. We will also assume that the analysis is performed on a general 3D domain unless specific remarks for 2D or 1D cases are made.

While Equation (1) already allows for the calculation of a valid strain field directly through a symmetric gradient operator $\nabla^{s}\left(•\right)=\frac{1}{2}\left[\nabla \left(•\right)+\nabla^{t}\left(•\right)\right]$, there is a couple of important basic remarks worth making at this point.

### 2.1. Kinematic Consistency of Boundary Condition Imposition

A discretised element whose kinematics are governed by Equation (1) does not allow a consistent imposition of boundary conditions on any traditional global FEM solution engine. This is because the displacements on the borders of the $\Omega^{-}$ domain of the element (i.e., the nodes on it) are directly reachable through the vector $\bar{u}$, while the displacements on the borders of $\Omega^{+}$ are rather a composition of $\bar{u}$ and $\left[\left|u\right|\right]$. We can use the vector $\bar{u}$ alone to impose boundary conditions on $\Omega^{-}$, but not on $\Omega^{+}$ since the actual displacement in this region remains a composition of two different vectors $\bar{u}+\left[\left|u\right|\right]$.

**Figure 4:**
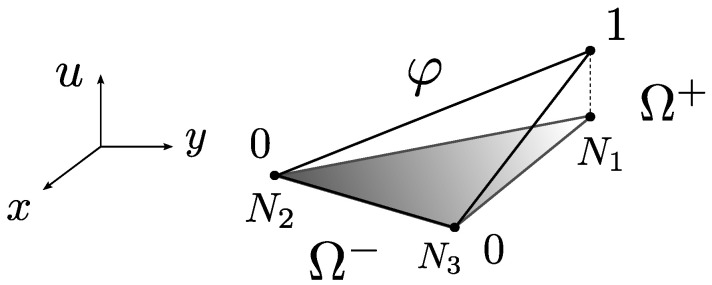
Example of the φ function for a constant stress triangle defined by the nodes $N_{1},N_{2},N_{3}$, with node 1 on the $\Omega^{+}$ domain and the others on $\Omega^{-}$. Note that for this case there is only one possible linear φ definition.

This issue has been well identified even from the first evolving steps of the framework and also treated by Oliver [10] through the introduction of the so-called auxiliary function φ. The function φ is defined as to allow for the existence of a new displacement vector

(2) $$\hat{u}=\bar{u}+\varphi \left[\left|u\right|\right],$$

by letting φ have a single property: it will have the value of 1 on any node lying on the border of $\Omega^{+}$ and zero in those on the remaining $\Omega^{-}$ border:

(3) $$\varphi =\left\{\begin{array}{cc} 1 & x\in \Omega^{+}\\ 0 & x\in \Omega^{-} \end{array}\right.,\;i=1,..,N$$

where $x_{i}$ denotes a nodal position. This is the only formal mathematical requirement as stated in [10] apart from the basic fact that φ should be continuous. The most common form of φ taken from the literature is yet the simplest one to satisfy Equation (3) for a given element geometry. For example, for the linear tetrahedron, the simplest φ remains a composition of standard linear interpolation functions. In this case, φ also happens to be unique. Analogous constructions for φ can be easily made for a 2D triangle and for 1D cases (Figure 4).

If φ complies with the previous definition, then the new vector $\hat{u}$ can be seen as a continuous fusion of the local displacement field and the jump vector associated with the fracture, having border displacements that coincide with those of u. Thus, for the practical purposes of nodal displacements in a discretised element, $\hat{u}$ contains all the correct information of border nodes and can be used for imposing boundary conditions in a direct and unequivocal fashion. Note that, on the other hand, the displacement field within the element does not coincide with that of u. $\hat{u}$ can be seen as a special version of u where the discontinuity jump is continuously fused by φ along the element domain. φ could be thus interpreted as a sort of regularisation function (see Figure 3).

With this definition in mind, Equation (1) is rewritten as

(4) $$u=\hat{u}+\left(H_{\Gamma }-\varphi \right)\left[\left|u\right|\right],$$

where the previous vector $\bar{u}$ has been replaced by the newly defined vector $\hat{u}$, and the discontinuity base function now becomes composed of both a Heaviside and φ. From this point, authors generally stop referring to $\bar{u}$ and simply take $\hat{u}$ as the de facto standard solution vector for the reasons mentioned above, and Equation (4) is taken as the reference for developing all derived mechanical fields.

While the introduction of φ solves a fundamental consistency problem on boundary conditions imposition, it is also responsible for other undesired effects on general element kinematics and fracture mode coupling. A deeper discussion is presented in Section 4.

### 2.2. Kinematics at Terminal Separation Conditions and Meaning of $\left[\left|u\right|\right]$

Post-localisation behaviour within an element is generally modelled by making use of one of two popular options: a continuous strong discontinuity approach (CSDA) or a discrete strong discontinuity approach (DSDA) [18,19]. Regardless of the approach taken, usually there is an initial mechanical connection between the newly split bodies, which will harden and/or decay as the crack separation evolves depending on the governing post-localisation law. Eventually, there should be no influencing forces from $\Omega^{+}$ to $\Omega^{-}$ when the split bodies have sufficiently separated. This state of failure will be referred to as a terminal separation condition. In such state, the traction happening at the fracture surface should be zero, and the internal fracture kinematics vector $\left[\left|u\right|\right]$ will practically represent the rigid body motion of the $\Omega^{+}$ domain with respect to $\Omega^{-}$, depending on the definition chosen for the vector $\left[\left|u\right|\right]$.

$\left[\left|u\right|\right]$ in most of the current E-FEM literature has been simply defined as a one-to-three components vector of constant values in space at the element scale, describing a rigid body translation of $\Omega^{+}$ with respect to $\Omega^{-}$. However, as $\left[\left|u\right|\right]$ stands as an internal element definition with no strong global restrictions, it could be arranged to describe more complex kinematic modes such as rigid body rotations, having non-constant component behaviour through the failure surface $\Gamma_{d}$.

**Figure 5:**
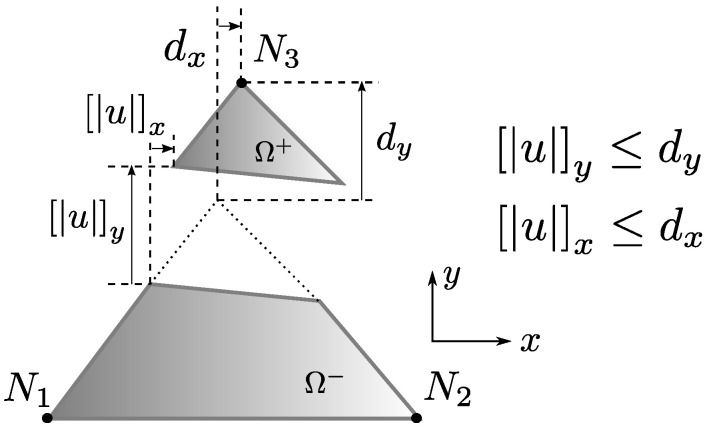
Example of kinematic consistency that should hold between the fracture displacement vector $\left[\left|u\right|\right]$ and the nodal displacement vector d for a constant stress triangle.

$\left[\left|u\right|\right]$ thus remains a free modelling choice in this sense. However, whatever this choice may be, its behaviour should be consistent with the standard element kinematics (whether u or $\hat{u}$). An obvious example is that the magnitude of a constant $\left[\left|u\right|\right]$ in the normal direction $\hat{n}$ to the discontinuity plane (crack normal separation) should be no greater than the magnitude of the relative nodal displacement in the element giving rise to this separation. This is shown in Figure 5 for a 2D constant stress triangle (CST). A relative displacement $d_{n}$ is imposed on this element and, assuming post-localisation, a corresponding crack separation ${\left[\left|u\right|\right]}_{n}$ develops. By common sense, it should be expected that ${\left[\left|u\right|\right]}_{n}\leq d_{n}$, where at terminal separation conditions, we could probably have ${\left[\left|u\right|\right]}_{n}≃d_{n}$ depending on the specific model.

Considering this, it is important to remark the framework does **not** explicitly guarantee any kinematic consistency between the internal variable $\left[\left|u\right|\right]$ and a standard global variable $\hat{u}$ or any derived quantities such as $d_{n}$. This is due to the same fact that $\left[\left|u\right|\right]$ is an internal element definition. This is especially true when reaching a terminal separation condition, where there is no further influence of any post-localisation law derived through CSDA or DSDA that would imply any additional coercion to $\left[\left|u\right|\right]$ in any way. In a terminal separation condition, there is no other governing law than that of the purely natural kinematics arising from the modelling choices made in the basic formulation of the strong discontinuity, such as φ.

In the end, it should be clear that the physical meaning of $\left[\left|u\right|\right]$ should be carefully assessed with respect to the formulation choices made within the E-FEM framework. This will be further discussed in Section 4 and Section 5.

Having all this in mind, the strain field is extracted by taking the symmetrical gradient $\nabla^{s}\left(•\right)$ in Equation (4). This study will assume a constant vector $\left[\left|u\right|\right]$ through $\Gamma_{d}$, which implies that this strong discontinuity study will be limited to a rigid body translation of $\Omega^{+}$ with respect to $\Omega^{-}$. The strain field can be thus expressed as:

(5) $$\begin{array}{c} \varepsilon =\nabla^{s}u=\nabla^{s}\hat{u}+\nabla^{s}\left[\left(H_{\Gamma }-\varphi \right)\left[\left|u\right|\right]\right]\\ \Rightarrow \varepsilon =\nabla^{s}\hat{u}-{\left(\nabla \varphi ⊗\left[\left|u\right|\right]\right)}^{s}+{\left(\nabla H_{\Gamma }⊗\left[\left|u\right|\right]\right)}^{s}\\ \Rightarrow \varepsilon =\nabla^{s}\hat{u}\underset{{\hat{\varepsilon }}_{b}}{\underset{︸}{-{\left(\nabla \varphi ⊗\left[\left|u\right|\right]\right)}^{s}}}\underset{{\hat{\varepsilon }}_{u}}{\underset{︸}{+\delta_{\Gamma }{\left(\hat{n}⊗\left[\left|u\right|\right]\right)}^{s}}}, \end{array}$$

where the first tensor product is identified as the bounded part of the strong discontinuity strain ${\hat{\varepsilon }}_{b}$ and the second tensor product is the unbounded strain ${\hat{\varepsilon }}_{u}$. $\delta_{\Gamma }$ is the Dirac delta, with a trigger position corresponding to the failure surface $\Gamma_{d}$, just as the Heaviside was described previously.

## 3. In-Depth Analysis of Variational Foundations

The use of customised field enhancement functions on the E-FEM framework requires a variational principle that grants sufficient flexibility to effectively manage multiple independent mechanical fields when making element discretisation. After all, the E-FEM remains a mixed FE formulation. This is the main reason why the three-field Hu–Washizu principle has been the preferred approach for almost all authors working on the framework. There are, however, many details usually omitted in the description of its application that merit formal mathematical justification. This will allow the reader to know why some numerical choices prevail in the E-FEM literature and also to better understand the measures taken for improving the formulation explained in later sections. We take the Hu–Washizu functional for three independent fields of displacement, strain and stress $\left(u,\varepsilon ,\sigma \right)$ as the point of departure:

(6) $$\begin{array}{cc} \; & I_{HW}\left(u,\varepsilon ,\sigma \right)=\frac{1}{2}\int_{\Omega }\bar{\bar{\varepsilon }}:C:\bar{\bar{\varepsilon }}\;dV-\int_{\Omega }\bar{\bar{\sigma }}:\bar{\bar{\varepsilon }}\;dV\\ & +\int_{\Omega }\bar{\bar{\sigma }}:\nabla u\;dV-\int_{\Omega }f_{b}\cdot u\;dV-\int_{\partial \Omega }t\cdot u\;dA, \end{array}$$

where the double bar notation $\bar{\bar{•}}$ specifies a mechanical field on its 2nd order tensor format (the omission of it would mean that the field is on the Voigt vector notation), C is a fourth-order linear elasticity tensor, $f_{b}$ is a vector of body forces and t is a vector of traction forces as commonly found in the conventional FEM. To reach equilibrium, a stationary condition for this functional is required, and this implies taking the variation of it along with the variations associated with each of the displacement, strain and stress fields $\left(\delta I_{HW}\left(\delta u,\delta \varepsilon ,\delta \sigma \right)=0\right)$, leading to three independent equations, already written using a Voigt vector notation:

(7a) $$\begin{array}{c} \int_{\Omega }\partial \delta u^{t}\sigma \;dV-\int_{\Omega }\delta u^{t}f_{b}\;dV-\int_{\partial \Omega }\delta u^{t}t\;dA=0 \end{array}$$

(7b) $$\begin{array}{c} \int_{\Omega_{e}}\delta \sigma^{t}\left(\partial u-\varepsilon \right)dV=0 \end{array}$$

(7c) $$\begin{array}{c} \int_{\Omega_{e}}\delta \varepsilon^{t}\left(\sigma \left(\varepsilon \right)-\sigma \right)dV=0 \end{array}$$

It is worth noticing the difference made in Equation (7c) between $\sigma \left(\varepsilon \right)$, which is the stress field calculated from the constitutive relations (in this case through an elastic relation $\sigma \left(\varepsilon \right)=C\varepsilon$), and σ, which is the independent stress field to be found in the analysis. These fields are not necessarily equal, and the variational analysis will provide a specific relation between them.

The system of Equations (7a)–(7c) is the most commonly used form of the Hu–Washizu principle as seen in the E-FEM literature. The solution for the system, in this continuous format, inevitably returns the equilibrium of the body (already expressed in Equation (7a)), the strong kinematic compatibility and constitutive relations:

(8a) $$\begin{array}{c} \nabla u-\bar{\bar{\varepsilon }}=0 \end{array}$$

(8b) $$\begin{array}{c} \bar{\bar{\sigma }}\left(\varepsilon \right)-\bar{\bar{\sigma }}=0 \end{array}$$

From this point, any further step requires stating a field discretisation strategy, and flexibility will be drawn from Equations (8a) and (8b) as required, having cases where these are not necessarily satisfied in a strong manner.

### 3.1. A Word on the Discretisation Strategy

When using the Hu–Washizu functional, it is important to remember that the three fields of displacement, strain and stress $\left(u,\varepsilon ,\sigma \right)$ along with the other three fields of displacement variation, strain variation and stress variation $\left(\delta u,\delta \varepsilon ,\delta \sigma \right)$ are all completely independent. Therefore, each of the six fields will require a unique discretisation choice and its entirely up to each study to determine the most convenient field discretisation setup, keeping in mind that the main goal of the framework is to represent the effects of the strong discontinuity in the most physically consistent, yet numerically efficient way. Apart from this, the only formal restriction is to satisfy the system of Equations (7a)–(7c) at all costs.

It should be remembered that the discretisation strategy follows the outline of the SKON family of E-FEM approaches: the structure of the Hu–Washizu principle is exploited to render the formulation as physically consistent as possible, including both correct kinematics and statics simultaneously, resulting in a non-symmetric FE formulation.

#### 3.1.1. Displacement Field Discretisation

For discretising the displacement field u, authors naturally take advantage of the facts discussed in Section 2.1, and take only $\hat{u}$ into account:

(9) $$u=\hat{u}=Nd,$$

with N as a standard interpolation matrix and d the standard nodal displacement vector. The variation of the displacement field δu is also discretised in the same way, taking the displacement vector variation δd:

(10) δu=Nδd

Equation (9) implies that the displacement field to be found through this strategy does not exactly correspond to the initial definition of u in the interior of the element but will perfectly coincide with it on the nodes, and therefore the vector d contains always the real displacement of the element through its nodes. This is considered as enough information to be extracted from the displacement field as there is generally no interest in studying it in detail considering that the vector $\left[\left|u\right|\right]$ already contains embedded crack displacement information. The advantages emerge when dealing with Equation (7a), as the simplification of the displacement field (especially the displacement variation field) will allow reaching the classical standard FEM global solution form without any additional terms involved. Note that Equation (9) does not imply Equation (10) in any way: having the same interpolation matrix N is totally a strategic choice that is convenient at this point. This present work will comply with it.

#### 3.1.2. Strain Field Discretisation

Discretisation of the strain field ε is performed following all the terms described in Equation (5), giving this field the role for modelling all strong discontinuity kinematics on the element, including bound and unbound terms:

(11) $$\varepsilon =Bd+G_{s}\left[\left|u\right|\right]=Bd+\left(G_{sb}+G_{su}\right)\left[\left|u\right|\right],$$

where B is the standard matrix corresponding to the partial differentiations of N and $G_{s}$ is the corresponding matrix for the strong discontinuity terms, including a bounded part $G_{sb}$ and an unbounded part $G_{su}$. The specific form of the matrix operators $G_{sb}$ and $G_{su}$ is constructed by considering Equation (5) alone. Their structure is fixed and determined by the natural kinematics of the strong discontinuity model assumed. A strain field following such idea within the E-FEM framework is referred to as a Kinematically Enhanced Strain (KES).

The choice for the strain variation field stays analogous by considering the same standard strain term associated with standard displacement variations, but leaving a choice open for a matrix $G_{s}^{*}$ which, for reasons explained later (and also a deliberate choice), will adopt the same bounded-unbounded composition $G_{sb}^{*}$, $G_{su}^{*}$ and will be associated with the variation of the crack displacements $\delta \left[\left|u\right|\right]$:

(12) $$\delta \varepsilon =B\delta d+G_{s}^{*}\delta \left[\left|u\right|\right]=B\delta d+\left(G_{sb}^{*}+G_{sn}^{*}\right)\delta \left[\left|u\right|\right]$$

In this case, the specific form of the matrix operators $G_{sb}^{*}$ and $G_{su}^{*}$ is not determined by strong discontinuity kinematics directly. It is rather derived from the demands of the variational principle itself through the satisfaction of Equation (7), as it will be shown in the following sections. This means in principle that the components of this strain field attend more to *static considerations*. A strain field following such idea within the E-FEM framework is referred to as an Enhanced Assumed Strain (EAS).

#### 3.1.3. Stress Field Discretisation

Finally, the independent stress σ and stress variation fields δσ are simply designated in a free-form fashion:

(13a) $$\begin{array}{cc} \sigma & =Ss \end{array}$$

(13b) $$\begin{array}{cc} \delta \sigma & =S^{*}\delta s, \end{array}$$

where s is an independent stress vector with the state of stresses associated with each node of a given element (e.g., a six-component vector for a 3D, constant field). δσ would be its corresponding variation. The interpolation matrices S and $S^{*}$ are absolutely unrelated to any of the previously defined entities in the other mechanical fields.

#### 3.1.4. Calculated Stress Field Discretisation

The stress field coming from the constitutive relation $\sigma \left(\varepsilon \right)$ will be calculated by taking only the *bounded* part of ε. The boundedness of $\sigma \left(\varepsilon \right)$ despite having unbounded terms within ε has been discussed since the first attempts of consolidating the E-FEM framework [15]. It remains an accepted practice in current theoretical developments. Considering this, this stress field is expressed as:

(14) $$\sigma \left(\varepsilon \right)=C\varepsilon_{b}=C\left(Bd+G_{sb}\left[\left|u\right|\right]\right)$$

### 3.2. Application of the Discretisation Strategy

This is probably the hardest and the most loosely described mathematical development section in the E-FEM framework while it determines most of the operational conditions of the numerical solution. The definitions of the chosen discretisation strategy in the previous section are basically thrown into the variational principle represented by Equation (7) to study their mathematical implications. The intention in this section is to unfold key parts of the process that strongly regulate the structure of current formulations as well as of future proposals.

#### 3.2.1. Basic Orthogonality Analysis: The Role of $S^{*}$

We will start by taking Equation (7b), which brings the following expression after considering the definitions of u, ε, the fact that $\left[\left|u\right|\right]$ is a constant and that δs remains completely arbitrary:

$$\begin{array}{c} \int_{\Omega_{e}}\delta \sigma^{t}\left(\partial u-\varepsilon \right)dV=\int_{\Omega_{e}}\delta s^{t}S^{*t}\left(Bd-\left[Bd+G_{s}\left[\left|u\right|\right]\right]\right)dV=\delta s^{t}\int_{\Omega_{e}}S^{*t}G_{s}dV\left[\left|u\right|\right]=0 \end{array}$$

(15) $$\begin{array}{c} \Rightarrow \int_{\Omega_{e}}S^{*t}G_{s}dV=0 \end{array}$$

Equation (15) demands an orthogonality between $S^{*t}$ and $G_{s}$ in a weak sense. Given that $G_{s}$ has the specific purpose of modelling the kinematics of the strong discontinuity, it shall not be modified to satisfy Equation (15). Thus, $S^{*}$ has to comply. Note that this variational relation does not impose any form of restrictions to critical functions, as $S^{*}$ will not give shape to any *real* mechanical field. It could be stated that $S^{*}$ becomes just a support interpolation matrix that allows $G_{s}$ to exist. We can choose whatever kinematics we want for the strong discontinuity implied in $G_{s}$, as long as we can actually prove that this support interpolation matrix $S^{*}$ satisfying Equation (15) exists.

At this point, the specific form of φ comes into play, which is important to discuss because it determines the overall form of $G_{s}$. $G_{s}$ in turn is composed of bounded and unbounded parts $G_{sb}$ and $G_{su}$. Let us suppose now that we work on a linear tetrahedron, and thus with a linear φ=a+bx+cy+dz, which is one of the most prominent choices made in the E-FEM literature. With this assumption and considering the structure of Equation (5), expressions for matrix operators $G_{sb}$ and $G_{su}$ on $\left[\left|u\right|\right]$ can be devised as:

(16) $$G_{sb}=-\left[\begin{array}{ccc} \varphi_{,x} & 0 & 0\\ 0 & \varphi_{,y} & 0\\ 0 & 0 & \varphi_{,z}\\ \varphi_{,y} & \varphi_{,x} & 0\\ 0 & \varphi_{,z} & \varphi_{,y}\\ \varphi_{,z} & 0 & \varphi_{,x} \end{array}\right]=-\nabla^{\prime },\;G_{su}=-\delta_{\Gamma }\left[\begin{array}{ccc} n_{x} & 0 & 0\\ 0 & n_{y} & 0\\ 0 & 0 & n_{z}\\ n_{y} & n_{x} & 0\\ 0 & n_{z} & n_{y}\\ n_{z} & 0 & n_{x} \end{array}\right]=-\delta_{\Gamma }H_{s}$$

where $\varphi_{x},\varphi_{y},\varphi_{z}$ are the partial derivatives of φ and $n_{x},n_{y},n_{z}$ are the components of the normal vector $\hat{n}$ to the crack plane. If we assume for a first trial that $S^{*}$ remains constant, there will be only one single option to define it: the identity matrix $I_{6}$ (or a scalar multiple of it). Equation (15) can be then worked using integral properties of the Dirac delta to show that:

(17) $$\int_{\Omega_{e}}S^{*t}G_{s}\;dV=\int_{\Omega_{e}}I_{6}\left(-\nabla^{\prime }+\delta_{\Gamma }H_{s}\right)\;dV=V_{e}\nabla^{\prime }+A_{\Gamma }H_{s},$$

where $V_{e}$ is the element volume and $A_{\Gamma }$ is the area of the crack surface. It can be easily seen that, as $\nabla^{\prime }$ and *V* do not have any kind of relation to $A_{\Gamma }$ and $H_{s}$, Equation (17) can never be asserted as zero and thus a constant $S^{*}$ satisfying Equation (15) does not exist. Note that a constant $S^{*}$ was chosen to be consistent with the natural order for a stress field associated with a standard linear displacement field on a linear tetrahedron. There is no option but to go for a non-conventional definition for $S^{*}$. Fortunately, Hu–Washizu allows for this.

Using a linear $S^{*}$ would imply a linear interpolation matrix based on a series of four points $\left(x_{i}^{*},y_{i}^{*},z_{i}^{*}\right)$, which would make a total of 12 free parameters to achieve the sought orthogonality. Let us define the four associated interpolation functions $S_{i}^{*}=a_{i}^{*}+b_{i}^{*}x+c_{i}^{*}y+d_{i}^{*}z$ to these points. Equation (15) can then be worked as:

(18) $$-\left(\int_{\Omega_{e}}S^{*t}dV\right)\nabla^{\prime }-\left(\int_{A_{\Gamma }}S^{*t}dA\right)H_{s}=0,$$

leading to a linear system of the form:

(19) $$S_{i}^{*V_{e}}\varphi_{,j}+S_{i}^{*A_{\Gamma }}n_{j}=0,\;i=1,2,3,4\;j=x,y,z$$

where $S_{i}^{*V_{e}}$ are the interpolation functions $S_{i}^{*}$ integrated through the element volume $V_{e}$, and $S_{i}^{*A_{\Gamma }}$ are these same functions but integrated over the crack surface $A_{\Gamma }$. As there are a total of 12 independent equations to fulfil and $S^{*}$ has 12 free parameters, a unique solution for $S^{*}$ exists.

Note that this system will determine a very specific set of nodal positions $\left(x_{i}^{*},y_{i}^{*},z_{i}^{*}\right)$ that are absolutely unrelated to *real* element nodal positions. The base of $S^{*}$ is not the standard element base and thus it will be entirely different to that coming from S, which has to be the real one. This means that, from this very point, the framework is changed from a classical Galerkin scheme to an asymmetric Petrov-Galerkin scheme, a fact that almost all authors in the current E-FEM literature ignore. For more complex choices of φ or even a non-constant $\left[\left|u\right|\right]$, it can be shown by the aforementioned approach that a sufficiently elaborate $S^{*}$ can be chosen to satisfy Equation (15). This last point is the main takeaway from the work in Equation (7b).

#### 3.2.2. The Bridge between Real and Constitutive Stress Fields

Equation (7c) can be worked by using Equation (12) and noting that δd and $\delta \left[\left|u\right|\right]$ remain independent, giving rise to two new equations:

$$\begin{array}{c} \int_{\Omega_{e}}\delta \varepsilon^{t}\left(\sigma \left(\varepsilon \right)-\sigma \right)dV=\int_{\Omega_{e}}\left(\delta d^{t}B^{t}+\delta {\left[\left|u\right|\right]}^{t}G_{s}^{*t}\right)\left(\sigma \left(\varepsilon \right)-Ss\right)dV\\ =\delta d^{t}\int_{\Omega_{e}}B^{t}\left(\sigma \left(\varepsilon \right)-Ss\right)dV+\delta {\left[\left|u\right|\right]}^{t}\int_{\Omega_{e}}G_{s}^{*t}\left(\sigma \left(\varepsilon \right)-Ss\right)dV=0 \end{array}$$

(20a) $$\begin{array}{c} \Rightarrow \int_{\Omega }B^{t}\sigma \left(\varepsilon \right)dV=\int_{\Omega }B^{t}Ss\;dV \end{array}$$

(20b) $$\begin{array}{c} \Rightarrow \int_{\Omega }G_{s}^{*t}\sigma \left(\varepsilon \right)dV=\int_{\Omega }G_{s}^{*t}Ss\;dV \end{array}$$

Equation (20a) is the only equation that allows to establish a direct relation between the calculated stresses from the constitutive law $\sigma \left(\varepsilon \right)$ and the interpolated *real* stress Ss, since the form of B is known beforehand for a given standard element geometry. Equation (20a) is a weak equality involving $B^{t}$ as a weight factor. The form of S always remains a choice. For a constant real stress field, S will be a 6×6 identity matrix, whereas for a linear real stress interpolation, S will be a dense 6×24 matrix, and so on. Based on this choice and the form of B, Equation (20a) may or may not grant a strong equality between constitutive stresses and real interpolated stresses. In the case that S is chosen to match the order of $\sigma \left(\varepsilon \right)$, Equation (20a) effectively reduces to a strong equality. Otherwise, for the case in which we have a constant B and a constant S, a real stress vector s can still be calculated through a simple volume averaged integral regardless of the order of $\sigma \left(\varepsilon \right)$:

(21) $$s=\frac{1}{V_{e}}\int_{\Omega }\sigma \left(\varepsilon \right)dV$$

Note that, in any case, real stress calculations remain explicit and do not require methods such as a least-squares approach as in other mixed element variational frameworks [1,20].

#### 3.2.3. The Bridge between Real and Constitutive Traction Vectors

Equation (20b) is treated differently. As mentioned before, $G_{s}^{*}$ is assumed to be composed of bounded and unbounded terms as its counterpart $G_{s}$, and in this section, it will be clarified why. Usually, authors in the field choose at this point to enforce weak orthogonality conditions to each separate side of Equation (20b) since it becomes easier to devise the traction vector explicitly [14]. The present analysis will continue to do so. This creates once again two new relations:

(22) $$\int_{\Omega }G_{s}^{*t}Ss\;dV=\int_{\Omega }G_{s}^{*t}\sigma \left(\varepsilon \right)\;dV=0$$

The main idea is to derive an expression for the *real* traction vector T based on the *real* stress field (Ss), and then connecting it to the traction vector based on constitutive stress $T_{\varepsilon }$, so that T can be explicitly calculated. This can be achieved by developing the left and centre side of Equation (22), leading to:

(23) $$\int_{\Omega }G_{sb}^{*t}Ss\;dV+\int_{\Omega }G_{su}^{*t}Ss\;dV=\int_{\Omega }G_{sb}^{*t}\sigma \left(\varepsilon \right)\;dV+\int_{\Omega }G_{su}^{*t}\sigma \left(\varepsilon \right)\;dV$$

If we propose the specific form of the unbounded $G_{su}^{*}$ as exactly the same as $G_{su}=\delta_{\Gamma }H_{s}$, we can use again the Dirac delta’s properties to arrive at:

$$\begin{array}{c} \int_{\Omega }G_{sb}^{*t}Ss\;dV+\int_{\Gamma_{d}}H_{s}^{t}SsdA=\int_{\Omega }G_{sb}^{*t}\sigma \left(\varepsilon \right)\;dV+\int_{\Gamma_{d}}H_{s}^{t}\sigma \left(\varepsilon \right)\;dA \end{array}$$

(24) $$\begin{array}{c} \Rightarrow \int_{\Gamma_{d}}T\;dA=\int_{\Omega }G_{sb}^{*t}\left[\sigma \left(\varepsilon \right)-Ss\right]dV+\int_{\Gamma_{d}}T_{\varepsilon }\;dA, \end{array}$$

where it has been recognised that $H_{s}^{t}$ remains a stress projection operator over the crack surface having $\hat{n}$ as its normal, thus returning traction vectors at once operating over both stress fields. The main intent of assuming $G_{su}^{*}$ is to effectively reveal the traction vectors T and $T_{\varepsilon }$. Depending on the stress relation coming from Equation (20a), Equation (24) might be simplified. A strong equality between T and $T_{\varepsilon }$ is possible only if we can ensure that the respective stress fields are constant and equal to each other.

#### 3.2.4. EAS and Static Considerations—The Patch Test Condition

The zero implied on the left side of Equation (22) allows to define $G_{s}^{*}$ under the EAS approach. The arbitrary vector s can be then taken out for reaching the following weak orthogonality condition:

(25) $$\int_{\Omega }G_{s}^{*t}S\;dV=0$$

As the form S is chosen based on real physics, it is clear that $G_{s}^{*}$ must be determined by it. The works on Equation (24) have already required the unbounded part $G_{su}^{*}$ to be equal to $\delta_{\Gamma }H_{s}^{*}$ in order to obtain the traction vectors, so only the bounded part $G_{sb}^{*}$ can be used to satisfy Equation (25):

(26) $$\int_{\Omega }G_{sb}^{*t}S\;dV+H_{s}^{t}\int_{\Gamma_{d}}SdA=0,$$

where the final form of $G_{sb}^{*}$ will basically depend on the choice of S. At this stage, authors generally also seek to comply with the traditional FE *patch test* to ensure element density convergence for a given parent element by verifying that it is able to work with constant stress fields regardless of its underlying complexity [1,14,20]. This implies to **additionally** satisfy Equation (26) for the case of a constant S, regardless of the form already chosen for it. This is the reason why most of the authors working on the E-FEM framework try to choose a constant S from the beginning, so that only one single solution for Equation (26) is required, satisfying the patch test for once and for all. For that case indeed, the solution to Equation (26) simplifies to the expression commonly found in E-FEM literature for the SKON, EAS approach [21,22]:

(27) $$G_{sb}^{*}=-\frac{A_{\Gamma }}{V_{e}}H_{s},$$

for which $G_{sb}^{*}$ has also been set as a constant matrix, as well. For other cases that are less restrictive, $G_{sb}^{*}$ has to rise in complexity by simultaneously satisfying Equation (26) for both a constant and a higher degree S, in a process analogous to that one followed during the analysis in Section 3.2.1. This leads to a linear system on the coefficients for $G_{sb}^{*}$ similar to that of Equation (19).

Note that, no matter what the choice is, it inevitably leads to $G_{sb}\neq G_{sb}^{*}$, again departing from a classical Galerkin scheme to a Petrov–Garlekin one. This is the only way to retain consistent kinematic representation while remaining variationally consistent under the light of Hu–Washizu. In the proposals made in the next sections, we will retain the choice of a constant real stress field, along with a constant S and the validity of Equation (27).

#### 3.2.5. Final Traction Calculation

If a constant $G_{sb}^{*}$ is retained, Equations (21) and (24) allow further simplifications to reach a weak equality between traction vectors:

(28) $${\bar{T}}_{\varepsilon }=\bar{T}=T,$$

where we have used the fact that a constant σ yields a constant T. Having cleared this link, the right hand of Equation (22) finally leads a way to explicitly calculate a surface average of the traction vector $\bar{T}$, for then applying any pertinent damage, softening or traction–separation laws. The calculation is simply stated as:

(29) $$T=\frac{1}{A_{\Gamma }}\int_{\Gamma_{d}}T_{\varepsilon }\;dA=-\frac{1}{A_{\Gamma }}\int_{\Omega }G_{sb}^{*t}\sigma \left(\varepsilon \right)\;dV$$

From now on, traction vector calculations will be simply referring to T.

#### 3.2.6. Internal–External Force Balance

This part of the variational analysis has been left to last as it does not bring any remarkable implications. Equation (7a) can be stated as follows:

(30) $$\int_{\Omega }B^{t}\sigma \;dV=\int_{\Omega }N^{t}f_{b}\;dV+\int_{\Omega }N^{t}t\;dA,$$

where Equation (20a) can be used literally as it is to vanish the real stress field from the expression and leave all force balance calculations in terms of the calculated stress field $\sigma \left(\varepsilon \right)$:

(31) $$\int_{\Omega }B^{t}\sigma \left(\varepsilon \right)\;dV=\int_{\Omega }N^{t}f_{b}\;dV+\int_{\Omega }N^{t}t\;dA$$

Note that no strong equality between the stress fields is required to satisfy Equation (31) in any way. With this last equation, the application of the complete variational principle for a wide range of cases typically considered on the E-FEM framework is finished. The present work will now take all the basics presented in Section 2 and Section 3 to describe the general inconsistency problems on commonly proposed versions of the formulation in a precise manner.

## 4. Current Formulation Approaches and Associated Pathologies

The following analysis takes Equation (29) as a departure point. After developing $G_{sb}^{*}$ and $\sigma \left(\varepsilon \right)$, the following form can be reached:

(32) $$T=T_{e}+M{\left[\left|u\right|\right]}_{\hat{n}},$$

where $T_{e}$ is a traction vector depending only on the standard element displacement vector d, and M is a crack stiffness matrix. The vector ${\left[\left|u\right|\right]}_{\hat{n}}$ is the crack displacement vector expressed on the local frame $\hat{n}$.

Equation (32), which is expressed entirely on the local crack base ($\hat{n},\hat{t},\hat{m}$), is by itself probably the most important yet one of the most overlooked variationally-based expressions in this strong discontinuity analysis framework. Indeed, it grants information about the influence of the load-driven and the crack separation-driven parts on the overall evolution of the fracture traction T. The latter determines, along the chosen softening laws, the complete post-localisation mechanics of the element, including what happens in terminal separation conditions. Note that Equation (32) has been derived merely from the discretisation strategy of the framework and the pure kinematics model proposed for the strong discontinuity itself. It has absolutely nothing to do with the application of any further traction–separation law schemes. The following subsections describe different ways to handle Equation (32) depending on the general fracture modelling approach.

### 4.1. Single Mode Formulations

Single mode formulations are those that consider a single kinematic mode associated with the fracture within an element, normally a rigid body normal separation or a parallel sliding distance (the latter assuming a uniform fracture plane $\Gamma_{d}$). This implies the suppression of all other fracture kinematic modes. For illustrating the implications of this choice, Equation (32) can be taken under terminal separation conditions: that is, assuming all averaged traction components are driven down to zero after the crack has sufficiently developed. We present the resulting equation as a system taking each of the local fracture plane directions:

(33) $$\begin{array}{cc} \; & T_{n}=T_{en}+M_{nn}{\left[\left|u\right|\right]}_{n}+M_{nt}{\left[\left|u\right|\right]}_{t}+M_{nm}{\left[\left|u\right|\right]}_{t}=0\\ \; & T_{t}=T_{et}+M_{tn}{\left[\left|u\right|\right]}_{n}+M_{tt}{\left[\left|u\right|\right]}_{t}+M_{tm}{\left[\left|u\right|\right]}_{t}=0\\ \; & T_{m}=T_{em}+M_{mn}{\left[\left|u\right|\right]}_{n}+M_{mt}{\left[\left|u\right|\right]}_{t}+M_{mm}{\left[\left|u\right|\right]}_{t}=0 \end{array}$$

As an example, considering a single normal separation mode formulation, the model will be entirely based on ${\left[\left|u\right|\right]}_{n}$ and will automatically make ${\left[\left|u\right|\right]}_{t}={\left[\left|u\right|\right]}_{m}=0$. For the system given by Equation (33), this would imply:

(34) $$\begin{array}{cc} \; & T_{n}=T_{en}+M_{nn}{\left[\left|u\right|\right]}_{n}=0\\ \; & T_{t}=T_{et}+M_{tn}{\left[\left|u\right|\right]}_{n}=0\\ \; & T_{m}=T_{em}+M_{mn}{\left[\left|u\right|\right]}_{n}=0 \end{array}$$

The updated Equation (34) remains overconstrained and cannot be satisfied by a single value of ${\left[\left|u\right|\right]}_{n}$. Authors choosing this approach will only take the traction–separation equation corresponding to the chosen fracture mode to be satisfied. In this case, only the first equation in (34) depicting the general traction component $T_{n}$ will be driven to zero. A specific ${\left[\left|u\right|\right]}_{n}$ value will ensure this condition. This ${\left[\left|u\right|\right]}_{n}$ will evidently not satisfy the remaining traction–separation relations in the system. As the components $T_{et},T_{em}$ depend on the arbitrary disposition of the nodal displacement vector d, general traction components $T_{t},T_{t}$ will take totally non-physical values. This leads to the creation of undesired spurious internal forces that will produce severe stress locking. In order for single mode formulations to thrive during entire nonlinear FEM simulations, it is required to deliberately drive these crack surface traction components to zero during a numerical solution, along with any other stresses still associated with the crack. This can be achieved by simply applying an arbitrary decay explicitly to these or by setting them immediately to zero when localisation is detected on a given element [21].

The only conditions in which this kind of formulation will naturally drive all stresses down to zero is for a specific combination of load (through d) and element geometry orientation with respect to $\Gamma_{d}$ (coefficients $M_{nn},M_{tn},M_{mn}$). As it will be shown later in a more detailed analysis of M for a linear tetrahedron and a normal separation mode, these conditions correspond to purely axial strain on the $\hat{n}$ direction, having an element face parallel to a planar $\Gamma_{d}$.

Because of the aforementioned reasons, single mode formulations do not ensure physical sense for crack internal variables. However, they have the evident advantage of their simplicity and numerical solution speed, as the system to solve for $\left[\left|u\right|\right]$ becomes a single algebraic equation. As simple as it is, this kind of formulation has been successfully used for the modelling of complex fracture phenomena in heterogeneous, quasi-brittle materials [22,23,24]. It has granted satisfactory results for the global strength prediction and overall crack position of actual test specimens under tensile and compressive loads, even when considering triaxial confinement preloading conditions [25].

### 4.2. Full Crack Translation Formulations: The Role of φ

Full crack translation formulations consider all crack displacement components when articulating the traction–separation equation system. In terminal conditions, the full system depicted by Equation (33) is to be solved for all variables ${\left[\left|u\right|\right]}_{n},{\left[\left|u\right|\right]}_{t},{\left[\left|u\right|\right]}_{m}$. Regardless of the nature of the traction–separation decay (exponential, polynomial, etc.), Equation (33) remains strictly linear. A unique solution will always exist for $\left[\left|u\right|\right]$ given that M is not singular. A detailed analysis of M is thus relevant in this study.

The crack stiffness matrix M has a direct dependence on $G_{sb}^{*}$ and $\sigma \left(\varepsilon \right)$ (Equation (29)). $\sigma \left(\varepsilon \right)$ in turn depends directly on $G_{sb}$ (Equation (14)), and thus on φ (Equation (16)). As already discussed in Section 2.1, the nature of φ depends on the parent element, and authors typically take the simplest mathematical definition of it satisfying the basic requirements in Equation (3).

The case of a linear tetrahedron, a parent element will be taken again as an illustrating reference. The simplest φ is linear and easily constructed by stating linear interpolation functions taking the values of 1 or 0 depending on which nodes are on which side of the fracture surface. A 2D case is again depicted in Figure 4. Note that it is not required to use the same function φ for all three displacement components ${\left[\left|u\right|\right]}_{n},{\left[\left|u\right|\right]}_{t},{\left[\left|u\right|\right]}_{m}$. Note that in this case there is only one possible definition of a linear φ for this parent element, anyway.

The standard deformation matrix B can be expressed as a block partition in a node-wise manner:

(35) $$B=\left[\begin{array}{cccc} B_{1} & B_{2} & B_{3} & B_{4} \end{array}\right],$$

where each block $B_{i}$ can be further expressed as a function of the unit vector $\Psi_{i}={\left[\Psi_{ix},\Psi_{iy},\Psi_{iz}\right]}^{t}$ normal to the opposing face to each node *i* in the tetrahedron (this is naturally coming from the basic FEM formulation of this parent element):

(36) $$B_{i}=\frac{A_{i}}{3V_{e}}\left[\begin{array}{ccc} \Psi_{ix} & 0 & 0\\ 0 & \Psi_{iy} & 0\\ 0 & 0 & \Psi_{iz}\\ \Psi_{iy} & \Psi_{ix} & 0\\ 0 & \Psi_{iz} & \Psi_{iy}\\ \Psi_{iz} & 0 & \Psi_{ix} \end{array}\right],$$

where $A_{i}$ is the area of the opposite face to node *i*. Knowing that φ follows a similar structure as B but considering the selective condition in Equation (3), $G_{sb}$ can be expressed as a superposition of the blocks defined in Equation (36):

(37) $$G_{sb}=-\sum_{i}^{N_{e}}p_{i}B_{i},$$

where $p_{i}$ adopts a value of 1 on the nodes lying on $\Omega^{+}$, and zero otherwise.

On the other hand, a constant field σ will be assumed so that the EAS approach matrix $G_{sb}^{*}$ can be obtained using Equation (27). With Equation (14) in mind, Equation (29) can now be developed to obtain the following:

(38) $$\begin{array}{c} \underset{K_{e}}{\underset{︸}{\left(\frac{1}{V_{e}}\int_{\Omega }H_{s}^{T}CB\;dV\right)}}d+\underset{K_{m}}{\underset{︸}{\left(\frac{1}{V_{e}}\int_{\Omega }H_{s}^{T}CG_{sb}dV\right)}}\left[\left|u\right|\right] \end{array}$$

(39) $$\begin{array}{c} T=R^{T}K_{e}d+R^{T}K_{m}R{\left[\left|u\right|\right]}_{\hat{n}}=T_{e}+M{\left[\left|u\right|\right]}_{\hat{n}}, \end{array}$$

where R is a rotation matrix allowing the passage from a global coordinate crack displacement vector $\left[\left|u\right|\right]$ to a local-based ${\left[\left|u\right|\right]}_{\hat{n}}$. Working an explicit form for M yields finally:

(40) $$M=-\frac{1}{V_{e}}\left[\begin{array}{ccc} c_{n1}\hat{n}\cdot \Psi & c_{n2}\hat{t}\cdot \Psi & c_{n2}\hat{m}\cdot \Psi \\ c_{s}\hat{t}\cdot \Psi & c_{s}\hat{n}\cdot \Psi & 0\\ c_{s}\hat{m}\cdot \Psi & 0 & c_{s}\hat{n}\cdot \Psi \end{array}\right],$$

where · denotes a dot product ($\hat{t}\cdot \Psi ={\hat{t}}^{T}\Psi$) and $\Psi =A_{i}\sum \Psi_{i}$ follows the superposition brought by Equation (37). The constants $c_{n1},c_{n2},c_{s}$ are a function of the linear elastic material model parameters E,ν. The reader can find more details about the derivation of this compact expression for M in Appendix A.

**Figure 6:**
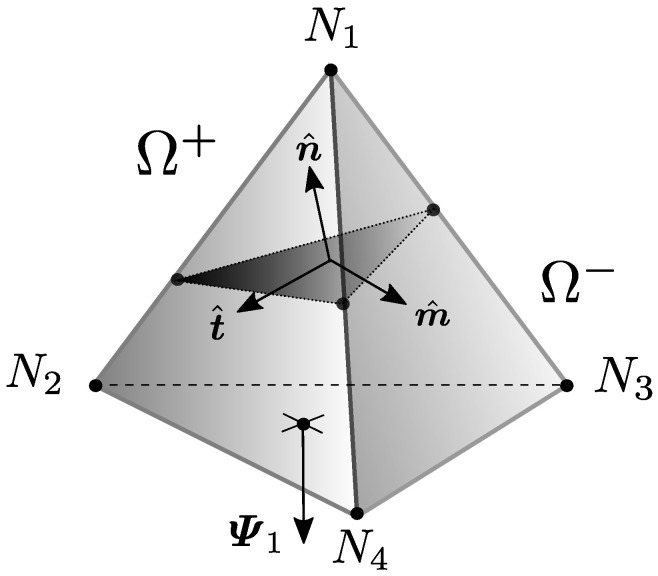
Tetrahedron element having a strong discontinuity with a basis $\hat{n},\hat{t},\hat{m}$ and a partition 3-1, node 1 located on $\Omega^{+}$.

For now, let us assume a fracture plane that makes a 3-1 partition of the tetrahedron’s nodes, having a single node on $\Omega^{+}$ (Figure 6), so that the interpretation of Ψ becomes easier. For the case of the linear tetrahedron, it is geometrically impossible to have the orientation of the opposite face to the single node to be perpendicular to $\hat{n}$, thus $\Psi \cdot \hat{n}$ will be always different than zero. Given this fact and the structure given by Equation (40), it can be safely assumed that M will not be singular. The system defined in (33) will then have a unique solution for ${\left[\left|u\right|\right]}_{n},{\left[\left|u\right|\right]}_{t},{\left[\left|u\right|\right]}_{m}$.

It is relevant to note the presence of coupling fracture stiffness coefficients between different directions ($M_{ij},i\neq j$). This means that, even at terminal conditions (Equation (33)), a normal displacement ${\left[\left|u\right|\right]}_{n}$ of the fractured piece of the element $\Omega^{+}$ will automatically induce a parallel rigid body motion ${\left[\left|u\right|\right]}_{t},{\left[\left|u\right|\right]}_{m}$ as well. This does not make physical sense since at this stage $\Omega^{+}$ is already a completely independent body provisioned with free rigid body displacement modes. Another way to see this would be that the traction vector components at the fracture are strongly coupled through ${\left[\left|u\right|\right]}_{n},{\left[\left|u\right|\right]}_{t},{\left[\left|u\right|\right]}_{m}$. Moving in a normal direction would mean to produce traction on the parallel direction. Again, this is not representative of fractured body kinematics.

Equation (40) also states that all coefficients $M_{ij}$ will depend in general on the orientation of the parent element’s geometry with respect to the fracture plane. That is, the $M_{ij}$ coefficients have an explicit mesh dependency. Thus, if we were to avoid any unwanted kinematic/traction coupling, the element geometry **must** have a specific orientation with respect to the fracture plane. Otherwise, while a mathematical solution for the system given by Equation (33) will always be assured, the overall physical sense of $\left[\left|u\right|\right]$ will be once again compromised. Wells [9] had already identified the influence of the function φ on the overall fracture kinematics and the induced strong coupling between traction components at the fracture plane. He proposed to fix it by redefining φ in such a way that the M components having the dot products $\Psi \cdot \hat{t}$ and $\Psi \cdot \hat{m}$ were driven to zero. That is, a φ capable of diagonalising M. Illustrating the process in two dimensions, if an initial function φ was defined for a CST element on the xy plane having partial derivatives $\varphi_{,x}$ and $\varphi_{,y}$, the following redefinition for the partial derivatives was made:

(41a) $$\begin{array}{c} \varphi_{,x}^{\prime }=n_{x}^{2}\varphi_{,x}+n_{x}n_{y}\varphi_{,y} \end{array}$$

(41b) $$\begin{array}{c} \varphi_{,y}^{\prime }=n_{y}^{2}\varphi_{,y}+n_{x}n_{y}\varphi_{,x} \end{array}$$

It is not necessary to check after φ directly, as only the partial derivatives of φ are needed during the numerical calculation.

**Figure 7:**
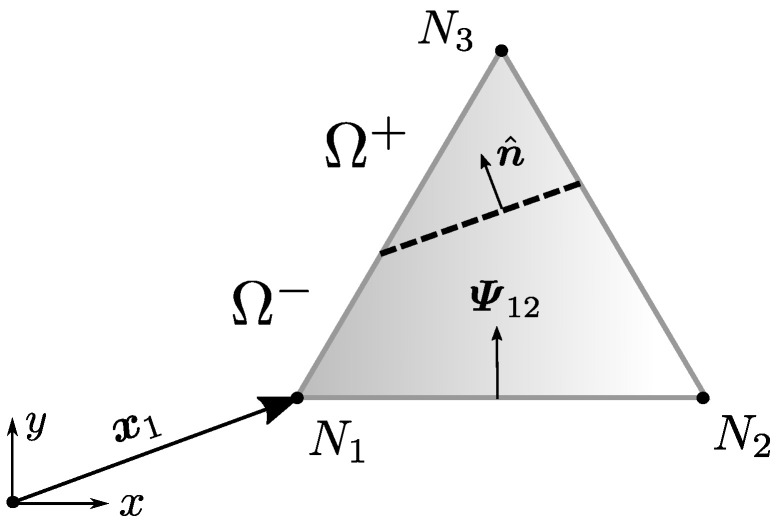
Two-dimensional CST element having a strong discontinuity with a vector $\hat{n}$ and a partition 2-1, node 3 located on $\Omega^{+}$. The vector Ψ, in this case, corresponds to the normal to the surface defined by nodes $N_{2},N_{3},N_{4}$.

Indeed, such a redefinition of φ will avoid the unwanted coupling of traction components. However, this φ will no longer be able to satisfy the basic requirements of Equation (3). Remember that this was the main reason for conceiving φ in the first place. If this alternate definition of φ is checked on the position $x_{1}$ shown in Figure 7 where it is supposed to be zero, it can be shown that:

(42) $$\varphi \left(x_{1}\right)\propto \left|\left(\hat{n}\times \Psi_{12}\right)\right|\left|\left(x_{1}\times \hat{n}\right)\right|,$$

where × denotes a cross product, $\Psi_{12}$ is the normal vector to the edge between nodes 1 and 2 and $x_{1}$ is the global position vector for node 1. This product of magnitudes nullifies whether the fracture line is aligned with the normal of the opposed element edge or if it is also aligned with the position vector in question. The reader can find more details in Appendix B.

Again, a mesh dependency is introduced, albeit more silent and rather impacting the pursuit of global equilibrium. It is clear that a deep knowledge of the fundamentals of the E-FEM framework is required for any kind of improvement proposals. This is the reason why this work has been devoted in the first half to describing its theoretical basis.

### 4.3. General Crack Kinematics Formulations

At terminal separation conditions, there should be no internal forces in the element associated with any rigid body motion of $\Omega^{+}$ with respect to $\Omega^{-}$. Otherwise, the global strength assessment on a complete FEM model will never be realistic since these elemental contributions are out of control, regardless of their already damaged state. Liberalisation of the three rigid body translation modes for $\left[\left|u\right|\right]$ ensures that it is possible to drive traction components $\bar{T_{n}},\bar{T_{m}},\bar{T_{n}}$ down to zero. Unfortunately, this does not ensure a complete release of element internal forces. Internal forces can be obtained extracting a part of the internal–external force balance in Equation (31):

(43) $$f_{int}=\int_{\Omega }B^{t}\sigma \left(\varepsilon \right)dV=\int_{\Omega }B^{t}\sigma dV=V_{e}B^{t}s,$$

where Equation (20a) and the fact that the present work stands for a constant stress field (σ=Ss=s) have been used. On the other hand, the kinematics proposed in Section 4.2 will only ensure the average traction vector T acting upon $\Gamma_{d}$ is driven down to zero:

(44) $$T=-\frac{1}{A_{\Gamma }}\int_{\Omega }G_{sb}^{*t}\sigma \left(\varepsilon \right)dV=0$$

For a constant stress parent element (constant $G_{sb}^{*},B$), it is obvious to see that Equation (44) does not imply reaching zero in Equation (43) since, in general, we have $G_{sb}^{*}\;\propto \;/\;B$. In this case, the whole real stress tensor on the element must be nullified so that all internal forces come to zero. Thanks to Equation (44), we also know that some of the components of this stress tensor expressed on the local coordinate base ($\hat{n},\hat{t},\hat{m}$) correspond precisely to the traction vector components that have been already worked out in Section 4.2. There are thus four components left without any damage process:

(45) $$\sigma :\left[\begin{array}{ccc} \sigma_{nn}=T_{n}\to 0 & \sigma_{nt}=T_{t}\to 0 & \sigma_{nm}=T_{m}\to 0\\ \sigma_{tn}=T_{t}\to 0 & \sigma_{tt} & \sigma_{tm}\\ \sigma_{mn}=T_{m}\to 0 & \sigma_{mt} & \sigma_{mm} \end{array}\right]$$

These untouched components $\sigma_{tt},\sigma_{tm},\sigma_{tt}$ (only three since the tensor is symmetric) should be damaged in such a way that the framework retains mathematical consistency and physical meaningfulness.

In order to damage σ, Equation (21) can be taken as a point of departure to relate a damaged state of stress σ to the constitutive stress $\sigma \left(\varepsilon \right)$. Then, Equation (14) is used to see the implications on specific terms within the formulation. At terminal conditions, nullifying σ will thus imply:

(46) $$\begin{array}{c} \int_{\Omega }\sigma dV=\frac{1}{V_{e}}\int_{\Omega }\sigma \left(\varepsilon \right)dV=\frac{1}{V_{e}}\int_{\Omega }C\left(G_{sb}\left[\left|u\right|\right]+Bd\right)dV=0 \end{array}$$

(47) $$\begin{array}{c} \frac{1}{V_{e}}\left(\int_{\Omega }G_{sb}dV\right)\left[\left|u\right|\right]=-\frac{1}{V_{e}}\left(\int_{\Omega }BdV\right)d \end{array}$$

(48) $$\bar{G_{sb}}\left[\left|u\right|\right]=-\bar{B}d$$

**Figure 8:**
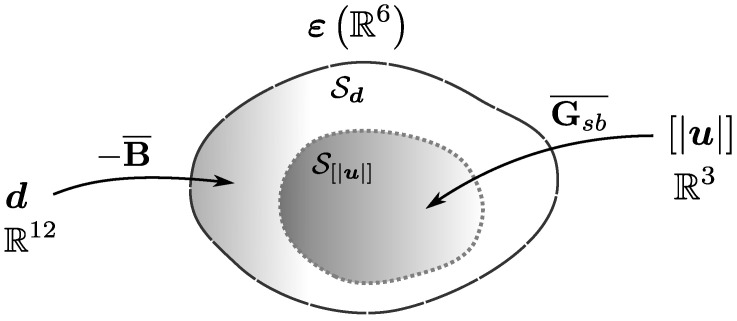
Generation of strain sub-spaces coming from d and $\left[\left|u\right|\right]$. The subspace generated by the fracture kinematics only represented through rigid body translations is not able to cover the subspace spanned by the nodal standard displacement vector.

In the end, Equation (48) makes a relation between two average strain fields: one coming from standard displacements and the other coming from the strong discontinuity enrichment function. For the case of a linear tetrahedron, the average standard strain field on the right side of Equation (48) maps a $R^{12}$ vector to a $R^{6}$ subspace $S_{d}$. We also have $\bar{B}=B$, as B is constant for this case. On the other hand, the left side enriched average strain maps a $R^{3}$ vector to a $R^{6}$ subspace $S_{\left[\left|u\right|\right]}$. If $G_{sb}$ happens to be constant, then we would also have $G_{sb}=\bar{G_{sb}}$. Despite the fact that we can stretch more demands to the matrix $G_{sb}$ through a richer definition of φ, it can be shown that, in general, $S_{\left[\left|u\right|\right]}\;⫆\;/\;S_{d}$. Figure 8 illustrates this situation. It is impossible to nullify the strain subspace spanned by d only counting on a three rigid body displacement mode vector $\left[\left|u\right|\right]$. Fracture kinematics **have** to be enriched to at least 6 modes to do so. Thus, even full crack translation models might need to force a stress damaging mechanism such as in single mode formulations discussed in Section 4.1 to avoid stress locking.

The idea of an extension for fracture kinematics variables on the E-FEM framework has been started by introducing the concept of a non-homogeneous (linear) crack displacement fields over a fracture surface in [26] in bi-dimensional elements. The concept was further consolidated by associating a set of fracture kinematic modes to this feature in [11,12] and later applied in [8,27,28], also for two-dimensional problems.

**Figure 9:**
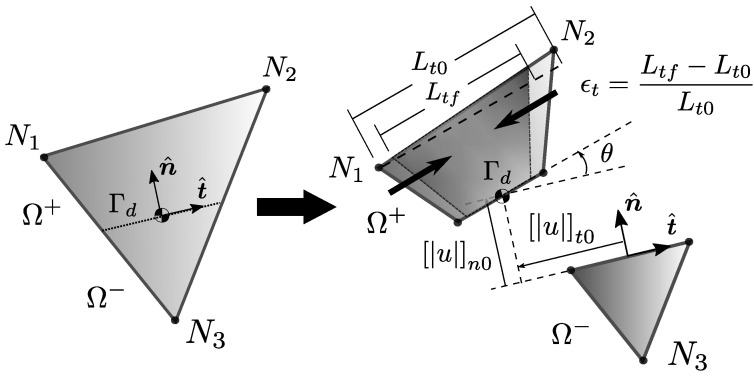
Enriched kinematics variables on the fracture local frame for a 2D CST element having a fracture line $\Gamma_{d}$ with orientation vectors $\hat{n},\hat{t}$ and nodes $N_{1},N_{2}$ lying on $\Omega^{+}$. This kinematic variable set is composed of two rigid body translations ${\left[\left|u\right|\right]}_{n0},{\left[\left|u\right|\right]}_{t0}$, one rigid body rotation θ and a single axial strain $\epsilon_{t}$ on the direction parallel to $\Gamma_{d}$. The centroid of $\Gamma_{d}$ is taken as the zero reference for rigid body motion description.

The approach is to consider that each of the components of $\left[\left|u\right|\right]$ are rather defined using linear functions instead of constant values. Each of the parameters within these linear functions is associated with general kinematics for the split body representing the $\Omega^{+}$ domain. This can include rigid body translations, rigid body rotations and even simple axial strains in specific directions. The centroid of the $\Gamma_{d}$ is generally taken as the reference position for the rigid body modes. In essence, fracture kinematics is enriched by providing $\Omega^{+}$ with improved modes that are able to represent more than just rigid body translations ${\left[\left|u\right|\right]}_{n},{\left[\left|u\right|\right]}_{t},{\left[\left|u\right|\right]}_{m}$. For a 2D case, Figure 9 illustrates how $\Omega^{+}$ kinematics on a CST element is enriched by considering two rigid body translations, a single rigid body rotation on the plane and a single extension (simple axial strain) on the parallel direction to $\Gamma_{d}$. This is the type of enrichment studied by Armero and Linder [11]. For the sake of readability, all descriptions on the rest of this section will take this CST triangle example as reference, knowing that the proposal in later sections will make a 3D generalisation of this approach.

The enrichment of fracture kinematics implies a redefinition for $\left[\left|u\right|\right]$ that introduces a new intermediary matrix J along with a more general fracture kinematics vector ξ:

(49) $$\begin{array}{c} \left[\left|u\right|\right]=J\xi \end{array}$$

(50) $$\begin{array}{c} \xi ={\left[\begin{array}{cccc} {\left[\left|u\right|\right]}_{n0} & {\left[\left|u\right|\right]}_{t0} & \theta & \epsilon_{t} \end{array}\right]}^{t} \end{array}$$

This enrichment has consequences on the mathematical framework developed in Section 2 and Section 3, starting by the basic strain expression derived in Equation (5), where the handling of gradients will change due to J. Additional interactions will also emerge between φ and this new matrix operator, yet giving rise to a different structure for the strain enrichment operators. Equations (47) and (48) will now be:

(51) $$\begin{array}{c} \frac{1}{V_{e}}\left(\int_{\Omega }G_{sb}JdV\right)\xi =\frac{1}{V_{e}}\left(\int_{\Omega }G_{sb}^{\prime }dV\right)\xi =-\frac{1}{V_{e}}\left(\int_{\Omega }BdV\right)d \end{array}$$

(52) $$\begin{array}{c} \bar{G_{sb}^{\prime }}\xi =-\bar{B}d, \end{array}$$

where a new compound operator $G_{sb}^{\prime }$ along with its averaged counterpart $\bar{G_{sb}^{\prime }}$ have been defined.

In order to build a new coherent structure for $G_{sb}^{\prime }$ under this scheme, authors typically choose not to rework Equation (5) and prefer to build $\bar{G_{sb}^{\prime }}$ directly in a numerical, column by column fashion [11,27]. Each of the columns of $\bar{G_{sb}^{\prime }}$ represent a link between a fracture kinematic mode $\xi_{k}$ and a specific nodal displacement state $d_{k}$:

(53) $$\bar{G_{sb_{k}}^{\prime }}\xi_{k}=-Bd_{k}$$

Here, the matrix $\bar{G_{sb}^{\prime }}$ is given physical meaning by linking kinematic states of the element standard nodes to each of the fracture kinematic modes. Since all other sections of the framework can be expressed as a function of $\bar{G_{sb}^{\prime }}$, this approach is enough to define the internal numerical solution process.

For example, suppose we would like to link the kinematic variable ${\left[\left|u\right|\right]}_{n0}$ to an actual set of nodal displacements $d_{{\left[\left|u\right|\right]}_{n0}}$ on the element. To do so, a real ${\left[\left|u\right|\right]}_{n0}$ is meant to represent a constant displacement of ${\left[\left|u\right|\right]}_{n0}$ for nodes $N_{1},N_{2}$ lying on $\Omega^{+}$ in the $\hat{n}$ direction. This specific displacement state $d_{{\left[\left|u\right|\right]}_{n0}}$ will generate a standard strain through $B=\bar{B}$. The negative of this strain has to be equal to the product of a specific column of $\bar{G_{sb}^{\prime }}$ (the first one) and the kinematic mode ${\left[\left|u\right|\right]}_{n0}$. The block structure of $B=\left[\begin{array}{ccc} B_{1} & B_{2} & B_{3} \end{array}\right]$ can be used to our advantage:

$$\begin{array}{c} {\bar{G_{sb}^{\prime }}}_{{\left[\left|u\right|\right]}_{n0}}{\left[\left|u\right|\right]}_{n0}=-Bd_{{\left[\left|u\right|\right]}_{n0}}\\ {\bar{G_{sb}^{\prime }}}_{{\left[\left|u\right|\right]}_{n0}}{\left[\left|u\right|\right]}_{n0}=-\left[\begin{array}{ccc} B_{1} & B_{2} & B_{3} \end{array}\right]\left[\begin{array}{c} {\left[\left|u\right|\right]}_{n0}\hat{n}\\ {\left[\left|u\right|\right]}_{n0}\hat{n}\\ 0 \end{array}\right]=-\sum_{i}^{N_{e}}p_{i}\left(B_{i}\hat{n}\right){\left[\left|u\right|\right]}_{n0} \end{array}$$

(54) $$\begin{array}{c} {\bar{G_{sb}^{\prime }}}_{{\left[\left|u\right|\right]}_{n0}}=-\sum_{i}^{N_{e}}p_{i}\left(B_{i}\hat{n}\right) \end{array}$$

The remaining columns ${\bar{G_{sb}^{\prime }}}_{{\left[\left|u\right|\right]}_{t0}},{\bar{G_{sb}^{\prime }}}_{\theta },{\bar{G_{sb}^{\prime }}}_{\epsilon_{t}}$ can be deducted following the same logic. This process allows to numerically define $\bar{G_{sb}^{\prime }}$ completely based on basic parent element characteristics.

One remark to this process is the nature of the *link* made between nodal displacements and kinematic variables (Equation (53)). A given mapping from the $R^{12}$ displacement space to the $R^{6}$ strain space is *not unique*, meaning that two or more different displacement states $d_{k}$ might correspond to exactly the same standard strain vector. This means that, even if we create a link between a specific fracture kinematic variable $\xi_{k}$ to a desired displacement state through a given $d_{k}$, the strain produced by $d_{k}$ could also be produced by other entirely different $d_{k}^{\prime }$, breaking the meaning of this link, and thus the physical meaning of $\xi_{k}$.

This is especially true when trying to distinguish what happens on $\Omega^{+}$ or $\Omega^{-}$. There might be cases in which nodes lying on $\Omega^{-}$ will contribute to a vector d capable of exciting unexpected modes $\xi_{k}$ that were originally associated with the kinematics of $\Omega^{+}$. Rigid body modes are reflexive in the sense that they can be interpreted by fixing a reference frame whether at $\Omega^{-}$ or $\Omega^{+}$, avoiding the risk of any mapping confusion. Simple axial strain modes are not, and these can be confused by this imperfect bijection. There is thus an inevitable kinematic coupling between $\Omega^{+}$ and $\Omega^{-}$ that prevents the approach from being fully objective. There is some work that has been undertaken, as an example, to consider a more general definition for the factor $p_{i}$: make it a continuous variable from 0 to 1 to control the coupling between $\Omega^{+}$ and $\Omega^{-}$ in a more flexible manner [29]. As this does not really solve the root problem, the authors of the present work have decided to keep the classical *binary* definition for $p_{i}$.

**Figure 10:**
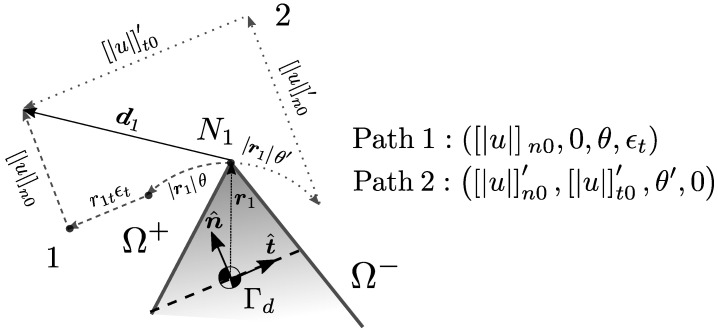
Illustration of linearly dependent kinematic mode decomposition for a CST having one single node on the $\Omega^{+}$ domain. Two different paths using different values for the kinematic modes are portrayed. Both paths (and endless others) lead to $d_{1}$ in this case.

Another issue with the approach, pointed out well by Armero and Linder [11], is the fact that it is assumed that the column decomposition of $\bar{G_{sb}^{\prime }}$ yields a set of vectors that compose a *linearly independent base* for building a ε space. For some node partitions on $\Omega^{+},\Omega^{-}$, specifically when we have a single node on $\Omega^{+}$, it is found that the base will not be linearly independent. This means that there is no way to know a unique solution for all $\xi_{k}$ modes for a given element kinematic state. Let us take the same CST triangle as an example, for a 1-2 node domain partition having node 1 on $\Omega^{+}$. For a given displacement $d_{1}$ of this node, there are infinite ways to make a ${\left[\left|u\right|\right]}_{n0},{\left[\left|u\right|\right]}_{t0},\theta ,\epsilon_{t}$ distribution in such a way that the resulting $\Omega^{+}$ motion returns the same $d_{1}$ displacement on node 1 (See Figure 10). Linder and Armero [11] proposed a numerical workaround to this problem by making use of Lagrange multipliers to relax unconstrained parameters during the solution process. This still leaves the question of physical meaningfulness and legitimacy of one solution over any other.

Finally, we already know the risk of forcing a definition of any $G_{sb}$-related operators without making a direct assessment of φ. None of the previous relations actually ensures satisfaction of any of the main requirements for φ in Equation (3). Linder and Armero [11] mention the possibility of integrating $\bar{G_{sb}^{\prime }}$ to inquire about φ, but this process is far from trivial given the multiple gradients implied in Equation (5). A lot of information about the specific φ giving rise to the given $\bar{G_{sb}^{\prime }}$ has been already lost at this point. The only evident fact would be that φ remains far from linear. The next section will take this development state as a departure point, and will continue to work on a 3D setting.

## 5. Formulation Approach Proposal for Three Dimensional Problems

This section makes a formulation proposal considering all the fundamentals from Section 2 and Section 3 as well as the issues found during the evolution of different strong discontinuity modelling approaches described in Section 4. At the end, this proposal is not meant to eradicate all the theoretical faults analysed so far. It is rather meant to do its best to reach a sound compromise between a theoretically sound E-FEM formulation but still remain operationally attractive. The key is to really retain mathematical robustness, ease of implementation and mesh independence as much as possible, which are the key characteristics that make the E-FEM framework attractive.

### 5.1. A Consistent Enrichment for φ

In Section 4.2, the role of the function φ has been discussed. φ allows for a consistent boundary condition imposition, but it also introduces a mesh dependency that generates an unwanted coupling between traction components at the fracture surface. This in turn compromises the physical meaning of fracture displacement variables ${\left[\left|u\right|\right]}_{n},{\left[\left|u\right|\right]}_{t},{\left[\left|u\right|\right]}_{m}$. Wells [9] proposed a fix, but it is not completely effective due to a violation of the mathematical framework.

The theory described in Section 2 and Section 3 can be used to make a consistent φ proposal. The idea is to propose a φ with enough flexibility to both satisfy the requirements in Equation (3) and the nullification of the out-of-diagonal terms of the M matrix in Equation (40), as well as any other eventual requirements. Recalling the analysis made in Section 3.2.1, it is allowed to increase the complexity for the definition for φ as long as it remains within the $C^{0}$ class of functions (continuous, derivative not necessarily continuous). It does not have to follow the same polynomial degree as the parent element. As an example, for a linear tetrahedron, a complete quadratic definition for φ is perfectly valid:

(55) $$\begin{array}{c} \varphi =P_{2}^{T}\alpha \\ \alpha =\left[\begin{array}{cccccccccc} \alpha_{0} & \alpha_{1} & \alpha_{2} & \alpha_{3} & \alpha_{12} & \alpha_{23} & \alpha_{13} & \alpha_{11} & \alpha_{22} & \alpha_{33} \end{array}\right]\\ P_{2}={\left[\begin{array}{cccccccccc} 1 & x & y & z & xy & yz & xz & x^{2} & y^{2} & z^{2} \end{array}\right]}^{t} \end{array}$$

This increase in order for φ implies an increase in order for the element strain field ε through $G_{sb}$ (Equation (11)), and thus for the calculated stress field $\sigma \left(\varepsilon \right)$. If the real stress field σ is chosen to follow the natural order in the parent element (a constant field for a linear tetrahedron), the strong constitutive equality (Equation (8b)) will not be possible and stresses σ will have to be calculated through volume averages (Equation (21)).

On the other hand, the advantage of having a discordance of $\sigma \left(\varepsilon \right)$ with respect to a constant σ field is that absolutely nothing will change in the treatment of the EAS part of the framework (Section 3.2.4). This means that Equation (27) can still be used for managing a constant expression for $G_{sb}^{*}$.

The integrand in Equation (29) is not constant anymore. This means that the integration process for all traction calculations will have to consider the geometric parameters of $\Gamma_{d}$ as well as the specific geometries of the subvolumes $\Omega^{+}$ and $\Omega^{-}$. Eventually, after vanishing all variable terms of the base $P_{2}$ through definite integration, an expression for the fracture stiffness matrix M as a function of φ coefficients α will be attained. As per Equation (40), we know that M remains *almost* symmetric, where the respective out-of-diagonal term pairs $\left(M_{ij},M_{ji}\right)$ share the same internal products $\left({\hat{t}}^{T}\Psi \right),\left({\hat{m}}^{T}\Psi \right)$, and that there is already a pair $\left(M_{tm},M_{mt}\right)$ that is zero. Thus, nullifying the out-of-diagonal terms in M for avoiding unwanted traction couplings implies the imposition of two additional linear equations on the α coefficients:

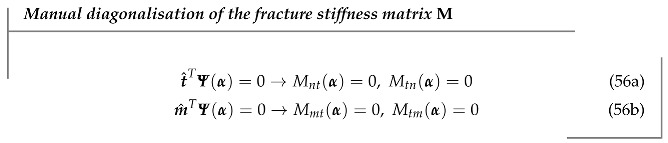


Adding this to the basic φ requirements (Equation (3), four linear equations on $k_{\varphi }$) makes a total of 6 linear constraints. As a complete quadratic base $P_{2}$ allows for a total of 10 free parameters, it is completely feasible to design a φ function capable of satisfying BC imposition consistency and at the same time the mechanical requirements for traction behaviour.

While the relations ((56a) and (56b)) ensure diagonalisation of the fracture stiffness matrix M, there is nothing that ensures that the magnitude of these diagonal components is physically meaningful. Each diagonal term $M_{jj}$ independently controls the fracture stiffness associated with each displacement ${\left[\left|u\right|\right]}_{j}$. These displacements should be consistent with the element standard nodal displacement vector d. As expected, φ can also help on this errand by imposing additional conditions for the diagonal fracture stiffness terms. Note that, as per Equation (40), the diagonal terms are *not* independent, and only one single constraint would be needed to regulate them all.

The case depicted in Figure 5 is taken as a reference for devising such constraints. For a 2-1 node partition on a 2D triangle, it is easy to find an exact relation between, for instance, the fracture normal separation ${\left[\left|u\right|\right]}_{n}$ and the normal displacement $\Delta d_{n}$ of a single isolated node partition on $\Omega^{+}$: they should be equal. For other configurations, however, such as a 2-2 node partition on a tetrahedron, it is not evident to assign a constraint on two independent nodes versus a single rigid body displacement: there are multiple ways to do so and none of them would be rigorously correct. This work proposes to take the *average* separation $\Delta \bar{d}$ between the groups of nodes on $\Omega^{+}$ and $\Omega^{-}$:

(57a) $$\begin{array}{c} \Delta \bar{d}=\bar{d^{+}}-\bar{d^{-}} \end{array}$$

(57b) $$\begin{array}{c} \Delta \bar{d}=\frac{\sum p_{i}d_{i}}{\sum p_{i}}-\frac{\sum \left(1-p_{i}\right)d_{i}}{\sum \left(1-p_{i}\right)}, \end{array}$$

Taking the normal direction $\hat{n}$, we require:

(58) $${\left[\left|u\right|\right]}_{n}=\Delta d_{n}={\hat{n}}^{T}\Delta \bar{d}$$

At this point, it will be assumed that φ has been already arranged as to diagonalise M through Equations (56a) and (56b). With this, at terminal conditions, we can practically use the first equation of the system in Equation (33) to finally arrive to a linear constraint for $M_{nn}$:

$$\begin{array}{c} T_{en}+M_{nn}{\left[\left|u\right|\right]}_{n}=0\\ {\hat{n}}^{T}K_{e}d+M_{nn}\left({\hat{n}}^{T}\Delta \bar{d}\right)=0 \end{array}$$

(59) $$M_{nn}\left(\alpha \right)=M_{nn_{\infty }}=-\frac{{\hat{n}}^{T}K_{e}d}{{\hat{n}}^{T}\left[\frac{F^{t}}{\sum p_{i}}-\frac{I_{3(4)}^{T}-F^{T}}{\sum \left(1-p_{i}\right)}\right]d}$$

A proper block matrix algebra has been used to express this constraint in terms of the complete vector d, using some auxiliary matrix blocks:

(60a) $$\begin{array}{c} F^{T}=\left[\begin{array}{cccc} p_{1}I_{3} & p_{2}I_{3} & p_{3}I_{3} & p_{4}I_{3} \end{array}\right] \end{array}$$

(60b) $$\begin{array}{c} I_{3(4)}^{T}=\left[\begin{array}{cccc} I_{3} & I_{3} & I_{3} & I_{3} \end{array}\right], \end{array}$$

having the 3×3 identity matrix $I_{3}$ as the basic block.

Note that in Equation (59), we are defining a component of M in terms of a nodal displacement vector d, which is the load imposed to the element. As the solution for all α coefficients takes place only once when reaching localisation, the load vector taken into account is precisely that corresponding to the closest localisation state for the element. For the kinematics to remain perfectly consistent under the definition of this $M_{nn}$ stiffness, the load path would have to remain proportional. This is highly unlikely during a global fracture phenomenon, since there are different loading and relaxation stages within the same element that would make d change direction in an aggressive fashion. $M_{nn}$ can be updated as well in such cases. This will imply more than one solution for the α coefficients. Imposing the constraint on a different component $M_{tt},M_{mm}$ will yield the same overall results given that a scaling factor of $c_{s}/c_{n1}$ is used as per Equation (40). As this represents only one additional linear constraint on the system for the α coefficients (making 7 constraints and 10 free parameters), once again it is still possible to build a proper φ function handling all requirements at once.

The next discussion will continue to explore the flexibility of φ under the light of fracture kinematics enrichment for 3D elements.

### 5.2. Fracture Kinematics Enrichment in 3D Coexisting with φ

In Section 4.3, an introduction has been made to a more general definition of fracture kinematics. The approach has been partially illustrated for a 2D parent element following the work in [11], where an explicit φ is practically disregarded. This section will describe a detailed application for a 3D element, including the clear distinction and managing of the φ function.

The process starts with the same definition of $\left[\left|u\right|\right]$ as a linear field vector, but for three dimensions in the local frame:

(61) $$\left[\left|u\right|\right]=\left[\begin{array}{c} {\left[\left|u\right|\right]}_{n}\\ {\left[\left|u\right|\right]}_{t}\\ {\left[\left|u\right|\right]}_{m} \end{array}\right]=\left[\begin{array}{c} a_{n}\eta +b_{n}\zeta +c_{n}\xi +d_{n}\\ a_{t}\eta +b_{t}\zeta +c_{t}\xi +d_{t}\\ a_{m}\eta +b_{m}\zeta +c_{m}\xi +d_{m} \end{array}\right],$$

where each of the parameters $a_{j},b_{j},c_{j},d_{j}$ is to be related to different fracture kinematic modes $\xi_{k}$. It can be shown that such linear field structure allows for the modelling of three rigid body translations ${\left[\left|u\right|\right]}_{n_{0}}$, ${\left[\left|u\right|\right]}_{t_{0}}$, ${\left[\left|u\right|\right]}_{m_{0}}$, three rigid body rotations $\theta_{n},\theta_{t},\theta_{m}$ and three axial deformations (associated with $\Omega^{+}$) $\epsilon_{n},\epsilon_{t},\epsilon_{m}$.

**Figure 11:**
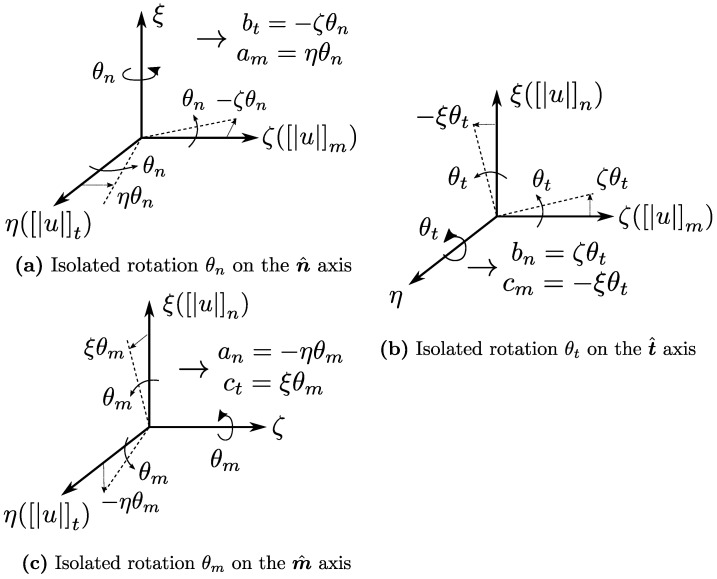
Relations between the parameters $a_{i},b_{i},c_{i},d_{i}$ and the rigid body rotation modes $\theta_{n},\theta_{t},\theta_{m}$. Note that two parameters may be influenced by the same rotation mode. Each plot (**a**), (**b**), (**c**) illustrates the effect of having a small rotation in the axes corresponding to $\theta_{n},\theta_{t},\theta_{m}$, respectively.

Figure 11 illustrates how to build the relations between all 3D rotation modes and the parameters $a_{j},b_{j},c_{j},d_{j}$. The general idea is to make a careful geometric interpretation for the meaning of each parameter by introducing a small variation while keeping all the remaining parameters at zero. This helps to build simple equations to relate these parameters to each fracture mode $\xi_{k}$. At the end, if small-angle approximations are allowed, the fields are finally expressed as:

(62) $$\left[\begin{array}{c} {\left[\left|u\right|\right]}_{n}\\ {\left[\left|u\right|\right]}_{t}\\ {\left[\left|u\right|\right]}_{m} \end{array}\right]=\left[\begin{array}{c} -\theta_{m}\eta +\theta_{t}\zeta +\epsilon_{n}\xi +{\left[\left|u\right|\right]}_{n_{0}}\\ \epsilon_{t}\eta -\theta_{n}\zeta +\theta_{m}\xi +{\left[\left|u\right|\right]}_{t_{0}}\\ \theta_{n}\eta +\epsilon_{m}\zeta -\theta_{t}\xi +{\left[\left|u\right|\right]}_{m_{0}} \end{array}\right]=J\xi$$

From this point, an expression for the intermediary matrix J can be readily made having a definition for the vector ξ:

(63) $$J\xi =\left[\begin{array}{ccccccccc} 1 & 0 & 0 & 0 & \zeta & -\eta & \xi & 0 & 0\\ 0 & 1 & 0 & -\zeta & 0 & \xi & 0 & \eta & 0\\ 0 & 0 & 1 & \eta & -\xi & 0 & 0 & 0 & \zeta \end{array}\right]\left[\begin{array}{c} {\left[\left|u\right|\right]}_{n_{0}}\\ {\left[\left|u\right|\right]}_{t_{0}}\\ {\left[\left|u\right|\right]}_{m_{0}}\\ \theta_{n}\\ \theta_{t}\\ \theta_{m}\\ \epsilon_{n}\\ \epsilon_{t}\\ \epsilon_{m} \end{array}\right]$$

With this analysis, Equation (5) can be worked for a general definition for the strain field ε considering both ξ and φ. As all structures having to do with the enriched kinematics and J are in the local fracture surface frame (ξ,η,ζ), it makes sense to work all further developments on the local frame from now on. The same bounded–unbounded structure for ε can be derived:

(64a) $$\begin{array}{c} \varepsilon =Bd+\left[G_{sb}^{\prime }+G_{su}^{\prime }\right]\xi \end{array}$$

(64b) $$\begin{array}{c} G_{sb}^{\prime }=H_{\Gamma }\nabla J-\left(\varphi \nabla J\right)-\nabla \varphi J \end{array}$$

(64c) $$\begin{array}{c} G_{su}^{\prime }=\delta_{\Gamma }H_{s}J \end{array}$$

The term $\left(\varphi \nabla J\right)$ has been grouped because it is *not* the product of a deformation gradient operator on J and φ, but rather a compound operator. The Heaviside function $H_{\Gamma }$ also forces a distinction between a bounded operator defined on $\Omega^{+}$ ($G_{sb}^{\prime +}$) and on $\Omega^{-}$ ($G_{sb}^{\prime -}$).

In general, ($G_{sb}^{\prime \pm }$) will be an explicit function of φ coefficients α. To start the kinematic linking process between fracture modes and nodal displacements followed in Section 4.3, the requirement stated in Equation (52) can be worked on a subvolume-basis:

(65) $$\bar{G_{sb}^{\prime \pm }}\xi =\frac{1}{V^{\pm }}\int_{\Omega^{\pm }}G_{sb}^{\prime \pm }dV\xi =-\bar{B}d,$$

where the averaged operators $\bar{G_{sb}^{\prime \pm }}$ will be explicitly linear matrices on the coefficients α.

The linking of each kinematic mode will establish a series of linear equations on each of the column elements of $\bar{G_{sb}^{\prime \pm }}$. For instance, the first mode ${\left[\left|u\right|\right]}_{n_{0}}$ already studied in Section 4.3 for two dimensions, would require satisfaction of exactly the same form of Equation (54). Fortunately, it can be shown that the first column of $\bar{G_{sb}^{\prime \pm }}$ has exactly the same zeros as $B_{i}$ so that in the local base, the following would be obtained:

(66) $${\bar{G_{sb}^{\prime \pm }}}_{{\left[\left|u\right|\right]}_{n0}}=\left[\begin{array}{c} {\bar{G_{sb}^{\prime \pm }}}_{1{\left[\left|u\right|\right]}_{n0}}\left(\alpha \right)\\ 0\\ 0\\ {\bar{G_{sb}^{\prime \pm }}}_{4{\left[\left|u\right|\right]}_{n0}}\left(\alpha \right)\\ 0\\ {\bar{G_{sb}^{\prime \pm }}}_{6{\left[\left|u\right|\right]}_{n0}}\left(\alpha \right) \end{array}\right]=-\sum_{i}^{N_{e}}p_{i}\left[\begin{array}{c} B_{ni}\\ 0\\ 0\\ B_{ti}\\ 0\\ B_{mi} \end{array}\right],$$

where the first index *l* in the ${\bar{G_{sb}^{\prime \pm }}}_{l{\left[\left|u\right|\right]}_{n0}}$ scalars is just the row placement. By looking at this structure, it can be seen that linking this kinematic mode requires satisfaction of three linear equations on the α coefficients. The specific expressions for ${\bar{G_{sb}^{\prime \pm }}}_{1{\left[\left|u\right|\right]}_{n0}},{\bar{G_{sb}^{\prime \pm }}}_{4{\left[\left|u\right|\right]}_{n0}},{\bar{G_{sb}^{\prime \pm }}}_{6{\left[\left|u\right|\right]}_{n0}}$ have to be retrieved by working φ and ξ in Equation (64b) and then taking the volume averaged integral demanded in Equation (65). Note that φ is to be expressed as a function of local coordinates (ξ,η,ζ), and the subvolume coordinate description in the local frame will also be required for evaluating the volume-averaged integrals.

The linking of some kinematic modes can be more demanding than others. Some will even repeat linear equations, and these can be naturally omitted, e.g., the first three columns of $G_{sb}^{\prime \pm }$, which contain repetitions of the simple partial derivatives $\varphi_{,\xi },\varphi_{,\eta },\varphi_{,\zeta }$. This depends on the symmetry of the kinematics defined and the model of the strong discontinuity overall. At the end, it is true that the demands for φ will do nothing but increase, as the φ free parameters are the only way to satisfy these relations. As a result, a full quadratic base proposal $P_{2}$ might not be enough to fulfil all these requirements additionally from Equation (3) and other constraints on the fracture stiffness matrix M: nullification of non-diagonal terms (Sys. (56)) and terminal separation conditions (Equation (59)). While increasing the polynomial base order of φ might be a quick and tempting option, there are also other options for improvement:

- As mentioned already in Section 2.1, it is not required to have the same φ structure associated for all fracture displacement components ${\left[\left|u\right|\right]}_{n},{\left[\left|u\right|\right]}_{t},{\left[\left|u\right|\right]}_{m}$. Indeed, three different functions $\varphi_{n},\varphi_{t},\varphi_{m}$ may coexist in the model:
  (67) $$\varphi \left[\begin{array}{c} {\left[\left|u\right|\right]}_{n}\\ {\left[\left|u\right|\right]}_{t}\\ {\left[\left|u\right|\right]}_{m} \end{array}\right]\to \left[\begin{array}{ccc} \varphi_{n} & 0 & 0\\ 0 & \varphi_{t} & 0\\ 0 & 0 & \varphi_{m} \end{array}\right]\left[\begin{array}{c} {\left[\left|u\right|\right]}_{n}\\ {\left[\left|u\right|\right]}_{t}\\ {\left[\left|u\right|\right]}_{m} \end{array}\right]$$
  While this might apparently triple the amount of free parameters, some of the model symmetries that are allowed for redundancy simplifications will no longer emerge. As an example, the basic deformation gradient operator on φ (the first three columns of $G_{sb}^{\prime \pm }$) will now have all different terms with respect to the one developed with a single φ:
  (68) $$\left[\begin{array}{ccc} \varphi_{,\xi } & 0 & 0\\ 0 & \varphi_{,\eta } & 0\\ 0 & 0 & \varphi_{,\zeta }\\ \varphi_{,\eta } & \varphi_{,\xi } & 0\\ 0 & \varphi_{,\zeta } & \varphi_{,\eta }\\ \varphi_{,\zeta } & 0 & \varphi_{,\xi } \end{array}\right]\to \left[\begin{array}{ccc} \varphi_{n,\xi } & 0 & 0\\ 0 & \varphi_{t,\eta } & 0\\ 0 & 0 & \varphi_{m,\varsigma }\\ \varphi_{n,\eta } & \varphi_{t,\xi } & 0\\ 0 & \varphi_{t,\varsigma } & \varphi_{m,\eta }\\ \varphi_{n,\varsigma } & 0 & \varphi_{m,\xi } \end{array}\right]$$
  On the other hand, basic φ requirements in Equation (3) will now require the triple of linear relations for boundary condition consistency: one set for each $\varphi_{n},\varphi_{t},\varphi_{m}$.
  (69a) $$\begin{array}{cc} \varphi_{n}\left(x_{i}\right) & =p_{i} \end{array}$$
  (69b) $$\begin{array}{cc} \varphi_{t}\left(x_{i}\right) & =p_{i} \end{array}$$
  (69c) $$\begin{array}{cc} \varphi_{m}\left(x_{i}\right) & =p_{i} \end{array}$$
  While there is evidently a trade-off, there is still a gain in the effective number of free parameters in the global φ structure, without the need to modify the complexity of the algebraic base of it.
- In general, φ has only a $C^{0}$ continuity requirement. This means that while the function itself is required to be continuous through space, its derivatives are not. This allows for a piece-wise definition for φ. The most natural choice is to propose a first function for $\Omega^{+}$ and then a second one for $\Omega^{-}$. Piece-wise φ does not increase the number of equations for Equation (3) requirements, but it still breaks some of the model symmetries. There will also be new linear equations to satisfy, which are associated with the basic $C^{0}$ continuity of φ at $\Gamma_{d}$:
  (70) $${\left.\varphi^{-}\right|}_{\Gamma_{d}}={\left.\varphi^{+}\right|}_{\Gamma_{d}},$$
  which will depend on the nature of the base P chosen for the structure of φ. For a $P_{2}$ base, $C^{0}$ continuity requires six linear relations. Again, the gain in free parameters is worth the trade-off.

By considering the combination of both options, φ can globally add up to 120 free parameters using a complete $P_{3}$ base. While this might seem just too much, the E-FEM model managed in [24], which also features a weak discontinuity to represent different material phases within the element, requires approximately 110 linear relations to reach *full consistency* as described in this work. This process is carried out only one time for each element that has reached localisation.

This last model is the one definitively used for later element simulations in this work. The reader can find the detail on the mode linking expressions as well as general φ handling on Appendix C.

### 5.3. A Comment on Linear System Handling

Calculation of all φ parameters based on all kinematic considerations may represent a formidable linear system depending on the parent element needs. The more complex the overall definition for φ is (Appendix C), the harder it is to ensure the well-posedness of this linear system on the α coefficients. This is basically the price to pay when building a more robust kinematic model depending on φ.

On the one hand, the linear dependency issues arising for some conditions during the construction of the columns of $\bar{G_{sb}^{\prime \pm }}$ (discussed in Section 4.3) will still be present on the 3D proposal. This will render sometimes the linear system globally overconstrained (for the total number of equations) and locally underconstrained (impossible to find a unique solution for some groups of parameters).

On the other hand, for complex φ structures, many symmetries in the overall kinematic model are lost. As an example, the structure of the M matrix deduced in Equation (40) is not guaranteed for a piece-wise, direction dependent, $P_{3}$ definition for φ. This implies that more α coefficients have to be used to *re-enforce* some desired constraints. It is not known by the authors of this work if this leads to the introduction of additional linear relations that might contradict some others already present in the system, thus compromising well-posedness.

To avoid the need of introducing complex numerical relaxation mechanisms during the solution of the α coefficients system, this work has used a Singular Value Decomposition (SVD) approach [30]. The SVD approach basically takes the system, whether under or overconstrained, and solves whether for the least-square-optimal solution if overconstrained or for the minimum value possible for free independent variables if underconstrained. The SVD decomposition is only made once the element reaches localisation. Once definite values are obtained for the α coefficients, the $\bar{G_{sb}^{\prime \pm }}$ can be numerically populated and these can be used for the rest of the load steps in the numerical analysis.

### 5.4. Further Treatment of the Traction–Separation Law System

The introduction of additional fracture variables poses questions on how to handle the traction–separation part of the framework. The procedure depends basically on how the virtual fracture displacement field $\delta \left[\left|u\right|\right]$ is discretised and the impact on the structure of Equation (25). While the real fracture displacement field vector $\left[\left|u\right|\right]$ effectively requires a 9 variable interpolation matrix through φ and J, the virtual counterpart $\delta \left[\left|u\right|\right]$**does not** have to share the same structure. $\delta \left[\left|u\right|\right]$ can still be assumed as a three constant field vector, or it can take the same detailed description as $\left[\left|u\right|\right]$. The Hu–Washizu framework allows this.

In this case, Equation (25) retains exactly the same structure having the real stress interpolation matrix S and the EAS operator $G_{sb}^{*}$. Despite the fact that now we have a $G_{sb}^{\prime \pm }$ considering the ξ related upgrades, the EAS definition for $G_{sb}^{*}$ does not have to consider any structures from the enriched fracture kinematics. The only requirement for the EAS definition of $G_{sb}^{*}$ is just to have the same bounded-unbounded composition featuring a Dirac delta and a projection operator $H_{s}$ as a minimum. This will allow the emergence and equality of the traction vector terms from Equation (24) onwards.

Equation (32) will return a new system with an enriched set of 9 variables in ξ:

(71) $$T=T_{e}+M\xi ,$$

where $T_{e}$ remains exactly the same as per Equation (38). Note that if the work has already been performed on the local frame, there is no need to use the rotation matrix R anymore. The expression for base fracture stiffness matrix M will have an updated definition:

$$\begin{array}{c} M=\left(\frac{1}{V_{e}}\int_{\Omega }H_{s}^{T}CG_{sb}^{\prime }dV\right)\xi =\frac{1}{V_{e}}H_{s}^{T}C\int_{\Omega }G_{sb}^{\prime \pm }dV \end{array}$$

(72) $$\begin{array}{c} M=\frac{1}{V_{e}}H_{s}^{T}C\left(V^{+}\bar{G_{sb}^{\prime +}}+V^{-}\bar{G_{sb}^{\prime -}}\right) \end{array}$$

Here, all matrices have been already assumed in the local frame to avoid further transformations, and the definitions implied in Equation (65) have been used. Note that matrix M now has dimensions 3×9.

The original three traction–separation laws associated with traction components $T_{n},T_{t}$, $T_{m}$ will return an underdetermined system:

(73a) $$\begin{array}{c} T_{n}=T_{en}+\sum_{k}M_{nk}\xi_{k}=q_{n}\left(\xi \right) \end{array}$$

(73b) $$\begin{array}{c} T_{t}=T_{et}+\sum_{k}M_{tk}\xi_{k}=q_{t}\left(\xi \right) \end{array}$$

(73c) $$\begin{array}{c} T_{m}=T_{em}+\sum_{k}M_{mk}\xi_{k}=q_{m}\left(\xi \right), \end{array}$$

where $q_{n},q_{t},q_{m}$ are decaying expressions controlling the overall magnitude of $T_{n},T_{t},T_{m}$ until reaching fracture terminal conditions, where $T_{n},T_{t},T_{m}$ should be zero.

To fully determine the fracture mechanics, six additional relations are needed. Three equations can be proposed to explicitly damage the $\sigma_{ij}$ terms left untouched by the original approach: $\sigma_{tt},\sigma_{tm},\sigma_{mm}$ (Equation (45)). This additional damaging process can be performed through additional traction–separation equations with specific decay behaviours. This brings a set of three additional equations to the system:

(74a) $$\begin{array}{c} \sigma_{tt}=T_{tt}+\sum_{k}M_{t_{tk}}\xi_{k}=q_{tt}\left(\xi \right) \end{array}$$

(74b) $$\begin{array}{c} \sigma_{tm}=T_{tm}+\sum_{k}M_{t_{mk}}\xi_{k}=q_{tm}\left(\xi \right) \end{array}$$

(74c) $$\begin{array}{c} \sigma_{mm}=T_{mm}+\sum_{k}M_{m_{mk}}\xi_{k}=q_{mm}\left(\xi \right), \end{array}$$

where the new load-driven terms $T_{tt},T_{tm},T_{mm}$ are calculated using the same structure as Equation (38), but now using stress projection operators $H_{st},H_{sm}$ in the remaining local directions $\hat{t},\hat{m}$, respectively:

(75) $$H_{st}=\left[\begin{array}{ccc} t_{x} & 0 & 0\\ 0 & t_{y} & 0\\ 0 & 0 & t_{z}\\ t_{y} & t_{x} & 0\\ 0 & t_{z} & t_{y}\\ t_{z} & 0 & t_{x} \end{array}\right],\;\mathrm{in}\;\mathrm{local}\;\mathrm{frame}:H_{st}=\left[\begin{array}{ccc} 0 & 0 & 0\\ 0 & 1 & 0\\ 0 & 0 & 0\\ 1 & 0 & 0\\ 0 & 0 & 1\\ 0 & 0 & 0 \end{array}\right]$$

(76) $$\begin{array}{c} H_{sm}=\left[\begin{array}{ccc} m_{x} & 0 & 0\\ 0 & m_{y} & 0\\ 0 & 0 & m_{z}\\ m_{y} & m_{x} & 0\\ 0 & m_{z} & m_{y}\\ m_{z} & 0 & m_{x} \end{array}\right],\;\mathrm{in}\;\mathrm{local}\;\mathrm{frame}:H_{sm}=\left[\begin{array}{ccc} 0 & 0 & 0\\ 0 & 0 & 0\\ 0 & 0 & 1\\ 0 & 0 & 0\\ 0 & 1 & 0\\ 1 & 0 & 0 \end{array}\right], \end{array}$$

and then using analogous expressions for new load-driven traction vectors $T_{et},T_{em}$ for finally projecting to $\hat{t},\hat{m}$ and obtaining the desired components:

(77) $$\begin{array}{c} T_{et}=K_{et}d=\left(\frac{1}{V_{e}}\int_{\Omega }H_{st}^{T}CBdV\right)d=H_{st}^{T}CBd \end{array}$$

(78) $$\begin{array}{c} T_{tt}=T_{et}\cdot \hat{t},\;T_{tm}=T_{et}\cdot \hat{m} \end{array}$$

(79) $$\begin{array}{c} T_{em}=K_{em}d=\left(\frac{1}{V_{e}}\int_{\Omega }H_{sm}^{T}CBdV\right)d=H_{sm}^{T}CBd \end{array}$$

(80) $$\begin{array}{c} T_{mm}=T_{em}\cdot \hat{m}, \end{array}$$

The stiffness vectors $M_{tt},M_{tm},M_{mm}$ are calculated in the same way as the rows of the original M matrix, but using the newly defined projection operators $H_{st},H_{sm}$:

(81) $$\begin{array}{c} M_{t}=\frac{1}{V_{e}}H_{st}^{T}C\left(V^{+}\bar{G_{sb}^{\prime +}}+V^{-}\bar{G_{sb}^{\prime -}}\right) \end{array}$$

(82) $$\begin{array}{c} M_{tt}={\hat{t}}^{T}M_{t},\;M_{tm}={\hat{m}}^{T}M_{t} \end{array}$$

(83) $$\begin{array}{c} M_{m}=\frac{1}{V_{e}}H_{sm}^{T}C\left(V^{+}\bar{G_{sb}^{\prime +}}+V^{-}\bar{G_{sb}^{\prime -}}\right) \end{array}$$

(84) $$\begin{array}{c} M_{mm}={\hat{m}}^{T}M_{m} \end{array}$$

It is important to remark that introducing the system of Equation (74) is not the same as enforcing an *artificial* decay on $\sigma_{tt},\sigma_{tm},\sigma_{tt}$ as in Section 4.1. We are systematically involving all fracture modes $\xi_{k}$ in both systems (Equations (73) and (74)). Simultaneous solutions of these systems will ensure kinematically and statically consistent values for ξ nullifying $\sigma_{tt},\sigma_{tm},\sigma_{tt}$, which should not exist at all once the crack develops.

The overall system in ξ still remains underdetermined. The last remaining three equations are free to implement more effects in fracture dynamics without having to resort to φ parameters exclusively. For example, three final relations could be established to finish the uncoupling of rigid body translations within the system in Equation (73) by grouping and isolating the effects of all other kinematic modes different than rigid body displacements:

(85a) $$\begin{array}{c} M_{n\theta_{n}}\theta_{n}+M_{n\theta_{t}}\theta_{t}+M_{n\theta_{m}}\theta_{m}+M_{n\epsilon_{n}}\epsilon_{n}+M_{n\epsilon_{t}}\epsilon_{t}+M_{n\epsilon_{m}}\epsilon_{m}=0 \end{array}$$

(85b) $$\begin{array}{c} M_{t\theta_{n}}\theta_{n}+M_{t\theta_{t}}\theta_{t}+M_{t\theta_{m}}\theta_{m}+M_{t\epsilon_{n}}\epsilon_{n}+M_{t\epsilon_{t}}\epsilon_{t}+M_{t\epsilon_{m}}\epsilon_{m}=0 \end{array}$$

(85c) $$\begin{array}{c} M_{m\theta_{n}}\theta_{n}+M_{m\theta_{t}}\theta_{t}+M_{m\theta_{m}}\theta_{m}+M_{m\epsilon_{n}}\epsilon_{n}+M_{m\epsilon_{t}}\epsilon_{t}+M_{m\epsilon_{m}}\epsilon_{m}=0 \end{array}$$

This way, the system is completely closed and a complete solution for ξ can be found at each load state d after element localisation. The approach ensures that $\sigma_{tt},\sigma_{tm},\sigma_{mm}$ becomes progressively suppressed as the fracture develops. On the other hand, it might seem awkward to have a $\delta \left[\left|u\right|\right]$ discretised very differently with respect to $\left[\left|u\right|\right]$, but this already happens with other fields within the framework.

## 6. Elemental Validations

In this section, calculations with a single element have been performed on many of the discussed formulations to illustrate the quality and pertinence of the results concerning fracture mechanics and element kinematics. Five models will be will be presented in a progressively complex fashion, going from a single mode approach up to the latest formulation proposal of Section 5. A graphical comparison will be made between all models, covering static behaviour (evolution of the fracture traction and its related stress state) and kinematic behaviour (consistency between internal kinematic modes and *true* element kinematics through d). This will allow to see how the framework gains robustness and physical meaningfulness overall as the formulations evolve.

**Figure 12:**
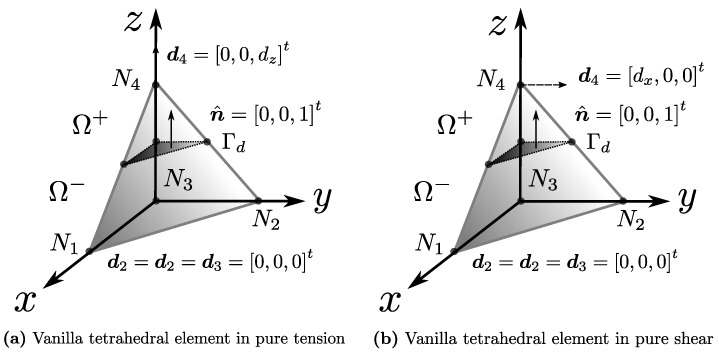
Schematics of typical vanilla elements taken for formulation testing at a small scale. Left element (**a**) for pure tension, right element (**b**) for pure shear.

Many authors working on the 3D version of the framework perform element-level calculations with an idealised regular element geometry and fracture orientations. The load direction is also often well-aligned to element edges or faces. Figure 12 shows examples of such cases on a linear tetrahedron for tension and shear testing. As discussed in previous sections, these conditions will hide the framework deficiencies since these are often the conditions in which almost all formulation versions will work properly. These will be referred to as *vanilla* conditions. Obtaining meaningful results using vanilla conditions at the element level does not guarantee successful numerical simulations with larger scale models.

It can be easily seen that even a simple large scale model such as a cubical material sample with a tetrahedral unstructured mesh in pure tension will generate far from vanilla conditions at the local element level. If element fracture planes are generated through free and spontaneous element localisations, a complex loading-unloading state will be observed with plane orientations having random partition types between nodes in all elements. In some elements, the combination of element geometry and the nodal displacement vector d will be such that it will activate complex kinematic modes (Section 5.2), and a formulation not prepared for this will inevitably fail to return physical or even mathematically meaningful results.

**Figure 13:**
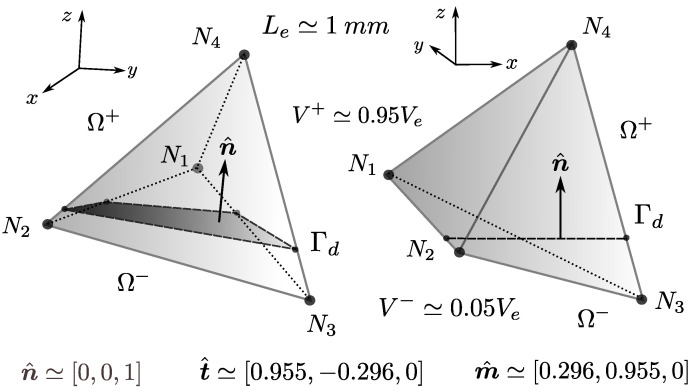
Two different views of a *realistic* element taken as the reference for the numerical simulations to test all formulations described in this work.

A robust E-FEM formulation should be able to undertake such scenarios to be truly useful. From the view of the authors of this work, there is no point in moving forward with a formulation if it is not capable of passing a *realistic* element test. This is the reason why this section presents the results at the element level. We have taken one of such non-vanilla element samples coming from a real large scale model, having a specific geometry and fracture plane orientation. An approximate illustration of the chosen element (again, a linear tetrahedron) is shown in Figure 13. It has a characteristic length of approximately 1 mm. The reference frame is such that the normal axis to the fracture plane $\hat{n}$ is almost perfectly aligned with the global *z* direction, but not to any of the tetrahedron’s faces. The remaining parallel directions $\hat{t},\hat{m}$ are not aligned with the x,y directions. It has a 2-2 node partition with the volume of $\Omega^{-}$ representing approximately 5% of total elemental volume while $\Omega^{+}$ has the rest.

**Figure 14:**
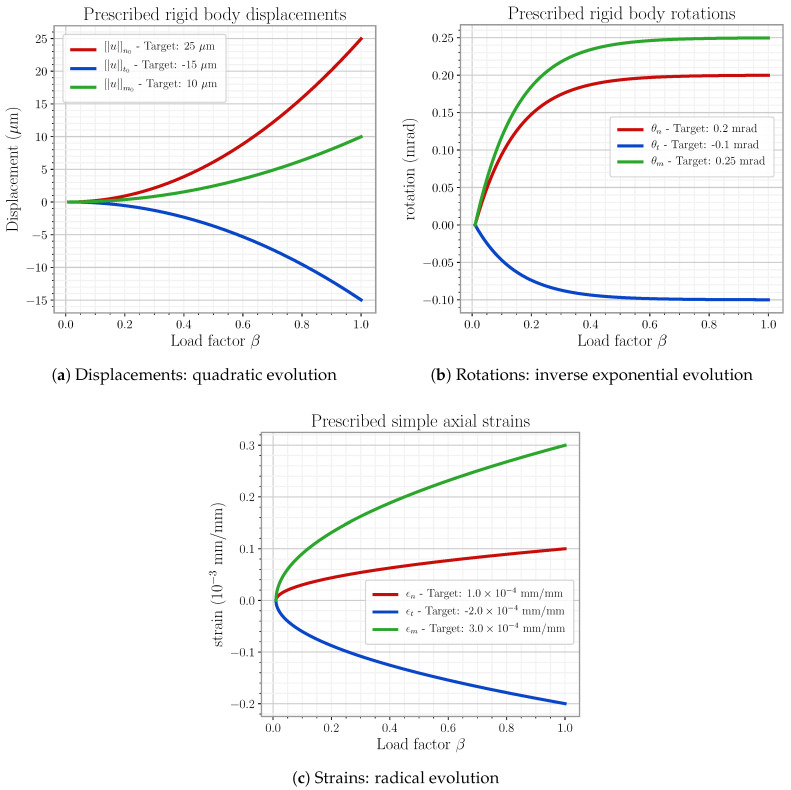
Proposed load evolution through a controlled behaviour of generalised kinematic modes. Part (**a**) prescribes a quadratic evolution for the displacement modes, part (**b**) prescribes an inverse exponential evolution for the rotation modes and in (**c**), a radical evolution is prescribed for simple axial strain modes.

The load displacement vector d has been built in such a way that it helps to validate the kinematic consistency of the formulations. For this, load requirements are proposed as a function of the generalised fracture kinematic modes $\xi_{k}$ described in Section 5.2. A monotonic, nonlinear behaviour is prescribed for each of these modes, reaching a final target value. A load progression factor β between 0 and 1 is used to calculate any intermediate value between the starting point (zero) and the target value. Figure 14a–c show the chosen behaviours for each $\xi_{k}$. For this case, a quadratic behaviour has been assigned for rigid body translations, an inverted exponential behaviour for rigid body rotations and a radical behaviour for simple axial strains.

The load demand overall remains a composition of strong rigid body displacements along with mild rotations and strains, having an emphasis on normal separation. **All** kinematic modes are activated without exception. The load proposed ensures the element arrives at the terminal separation conditions at target values.

The load displacement vector d associated with this particular evolution of the modes $\xi_{k}$ can be retrieved by knowing beforehand the nodes of the element corresponding to the $\Omega^{+}$ domain and the mode linking equations discussed in Section 5.2. Once again, expressions have been explicitly detailed in Appendix C. Starting from a load $d_{0}$ corresponding to element localisation, the effective d prescription can be calculated as a superposition of all effects:

(86) $$d\left(\beta \right)=d_{0}+\sum_{j}^{m,n,t}d_{{\left[\left|u\right|\right]}_{j}}\left(\beta \right)+\sum_{j}^{m,n,t}d_{\theta_{j}}\left(\beta \right)+\sum_{j}^{m,n,t}d_{\epsilon_{j}}\left(\beta \right),$$

where $d_{{\left[\left|u\right|\right]}_{j}}$ are the displacement vectors associated with rigid body translation demands, $d_{\theta_{j}}$ for rigid body rotations and $d_{\epsilon_{j}}$ the displacements related to simple axial strains.

Since the load requirements are finally prescribed based precisely on a controlled local fracture kinematics behaviour, a good E-FEM formulation should return a *kinematics calculation* ξ *as close to and most consistent as possible* with the source fracture kinematics, now referred to as $\xi_{ref}$: the kinematic reference.

As mentioned before, the element is assumed to have already gone through localisation with a given stress state $\sigma_{y}$ at the fracture plane:

$$\left[\begin{array}{ccc} \sigma_{y_{nn}} & \sigma_{y_{nt}} & \sigma_{y_{nm}}\\ \sigma_{y_{tn}} & \sigma_{y_{tt}} & \sigma_{y_{tm}}\\ \sigma_{y_{mn}} & \sigma_{y_{mt}} & \sigma_{y_{mm}} \end{array}\right]\to \sigma_{y}=\left[\begin{array}{c} \sigma_{y_{nn}}\\ \sigma_{y_{nt}}\\ \sigma_{y_{nm}}\\ \sigma_{y_{tt}}\\ \sigma_{y_{tm}}\\ \sigma_{y_{mm}} \end{array}\right]=\left[\begin{array}{c} 9.0\\ 0.0\\ 0.0\\ 2.5\\ 0.138\\ 1.459 \end{array}\right]\mathrm{MPa},$$

where $\sigma_{y_{nn}},\sigma_{y_{tn}},\sigma_{y_{mn}}$ correspond to the components of the traction vector $T_{n},T_{t},T_{m}$ at localisation. These are calculated through a specific localisation criterion in the element, which in this case has been a Rankine criterion. The remaining components $\sigma_{ytt},\sigma_{ytm},\sigma_{ymm}$ are found by projecting the entire state of stresses to $\hat{t}$ and $\hat{m}$ exactly at this load level.

The traction–separation equations consider an exponential decay law for all pertinent fracture traction/stress components:

(87) $$q_{j}=\sigma_{y_{j}}e^{-\left(\frac{\sigma_{y_{nn}}}{G_{fI}}{\left[\left|u\right|\right]}_{n_{0}}+\frac{\sigma_{y_{nt}}}{G_{fII}}{\left[\left|u\right|\right]}_{t_{0}}+\frac{\sigma_{y_{nm}}}{G_{fII}}{\left[\left|u\right|\right]}_{m_{0}}\right)},$$

where $\sigma_{y_{j}}$ are each of the aforementioned initial fracture yield stress components. Note that the exponential argument is exclusively controlled by the fracture kinematics corresponding to rigid body translations. $G_{fI},G_{fII}$ are the associated fracture energies for fracture separation ${\left[\left|u\right|\right]}_{n_{0}}$ and fracture sliding ${\left[\left|u\right|\right]}_{t_{0}},{\left[\left|u\right|\right]}_{m_{0}}$, respectively. This is a particular proposal that will conveniently fit all formulation types proposed so far, although richer proposals could be made for the generalised kinematics approaches.

All calculations have been made using SageMath 8.7 [31] mathematical open software on a Jupyter notebook 5.7.6 [32] platform. This has included all symbolic integrations to handle all enhancement functions parameters and operators such as φ, J and $\bar{G_{sb}^{\prime }}$. All numerical procedures such as SVD operations and linear system solutions have been treated in this platform, as well. Graphical visualisation of the realistic element in question (Figure 13) was also originally performed using these tools.

In the following subsections, five different formulation versions will be described in order of increasing complexity. At the end, a graphical comparison will be made on their kinematic and static behaviours: the evolution of the relevant fracture kinematic state variables and the fracture stress state variables over load progression starting from localisation.

### 6.1. Single Mode Formulation

For a single mode approach, the calculation begins by considering rigid body translations ${\left[\left|u\right|\right]}_{n}$, ${\left[\left|u\right|\right]}_{t}$, ${\left[\left|u\right|\right]}_{m}$ and having only one of them different than zero (Section 4.1). To follow the example, a normal separation calculation will be made, leaving thus ${\left[\left|u\right|\right]}_{t}=0,{\left[\left|u\right|\right]}_{m}=0$. The φ function remains linear and unique, so that $G_{sb}$ adopts its simplest way possible as a constant matrix (Equation (37)). The approach can be summarised in Equation (89).

The approach considers only the first equation of the traction–separation law system, and solves for ${\left[\left|u\right|\right]}_{n}$. This solution implies finding the intersection between a straight line $T_{en}+M_{nn}{\left[\left|u\right|\right]}_{n}$ and the nonlinear behaviour $\sigma_{yn}e^{-\frac{\sigma_{ynn}}{G_{fI}}{\left[\left|u\right|\right]}_{n}}$. The solution can be expressed in a closed, explicit form using the main branch of the Lambert function $W_{0}$ [22].

After a solution for ${\left[\left|u\right|\right]}_{n}$ is obtained, the approach then proceeds to calculate the remaining statics of the fracture plane, mainly the three fracture traction components $T_{n},T_{t},T_{m}$. At last, the remaining components of the state of stresses $\sigma_{tt},\sigma_{tm},\sigma_{mm}$ are retrieved for reference by calculating the entire stress σ (Equation (14)) and its projection to the corresponding directions.

### 6.2. Full Translation Formulations

The next step is to consider formulations that make complete liberalisation of all fracture displacement components ${\left[\left|u\right|\right]}_{n}$, ${\left[\left|u\right|\right]}_{t}$, ${\left[\left|u\right|\right]}_{m}$ (Section 4.2). Two versions from this family are considered for elemental simulations, depending on their assumptions made on the φ function. One considers a classical linear definition for φ (and therefore a constant $G_{sb}$), representing the direct extension of the single mode approach of the previous section. The other builds a quadratic φ that helps the diagonalisation of the M matrix as well as for kinematic consistency at terminal separation conditions. Regardless of the φ assumptions, these formulations require a solution for the three-variable nonlinear system:

(88) $$T=T_{e}+M\left[\left|u\right|\right]=\sigma_{y}e^{-\left(\frac{\sigma_{y_{nn}}}{G_{fI}}{\left[\left|u\right|\right]}_{n}+\frac{\sigma_{y_{nt}}}{G_{fII}}{\left[\left|u\right|\right]}_{t}+\frac{\sigma_{y_{nm}}}{G_{fII}}{\left[\left|u\right|\right]}_{m}\right)},$$

which still has a closed, analytical solution using condensation techniques and the same Lambert W function $W_{0}$ approach. The first three-mode approach is summarised in Equation (90).

For this case, the calculation of the traction vector components $T_{n},T_{t},T_{m}$ and the stress components $\sigma_{tt},\sigma_{tm},\sigma_{mm}$ is performed exactly the same way as with the single mode approach using the solution values for ${\left[\left|u\right|\right]}_{n},{\left[\left|u\right|\right]}_{t},{\left[\left|u\right|\right]}_{m}$.

The other three-mode approach will go forward and consider a more complex definition for the φ function, namely a quadratic one, as discussed at the beginning of Section 5.1 supported by a complete $P_{2}$ quadratic polynomial base (Equation (55)).

Ten free parameters (α coefficients) are available with this φ proposal. These will be used to build a linear system satisfying specific kinematic and static conditions: four of them to satisfy the basic boundary condition imposition requirements (Equation (3)), two for driving out-of-diagonal $M_{ij}$ fracture stiffness terms to zero (Equation (40)) and a last one to achieve consistent terminal separation conditions according to Equation (59). This makes seven linear constraints, which leaves three free parameters. An SVD approach is used (Section 5.3) to manage these conditions and to look for the α coefficient vector with the smallest norm possible.

(89) $$\begin{array}{c} \underline{\mathrm{Single Mode Formulation}}\\ \begin{array}{cccccc} \mathrm{Kinematic}\;\mathrm{modes}: & \; & & \; & & \;{\left[\left|u\right|\right]}_{n}\\ \mathrm{Linear},\;\mathrm{known}\;\varphi : & \; & & \; & & \varphi =\alpha_{0}+\alpha_{01}x+\alpha_{01}y\\ \mathrm{Fixed},\;\mathrm{constant}\;G_{sb}: & \; & & \; & & G_{sb}=-\sum_{i}^{N_{e}}p_{i}B_{i}\\ \mathrm{Traction}–\mathrm{separation}\;\mathrm{system}: & \; & & \; & & T=T_{e}+M{\left[\left|u\right|\right]}_{\hat{n}}=q\left({\left[\left|u\right|\right]}_{\hat{n}}\right)\\ \mathrm{Nullify}\;{\left[\left|u\right|\right]}_{t},{\left[\left|u\right|\right]}_{m}: & \; & & \; & & {\left[\left|u\right|\right]}_{t}=0,{\left[\left|u\right|\right]}_{m}=0\\ \mathrm{Solve}\;\mathrm{only}\;\mathrm{for}\;{\left[\left|u\right|\right]}_{n}: & \; & & \; & & T_{en}+M_{nn}{\left[\left|u\right|\right]}_{n}=\sigma_{y_{nn}}e^{-\frac{\sigma_{y_{nn}}}{G_{fI}}{\left[\left|u\right|\right]}_{n}}\\ \mathrm{Calculate}\;T_{n},T_{t},T_{m}: & \; & & \; & & T_{n}=T_{en}+M_{nn}{\left[\left|u\right|\right]}_{n}\\ \; & \; & & \; & & T_{t}=T_{et}+M_{tn}{\left[\left|u\right|\right]}_{n}\\ \; & \; & & \; & & T_{m}=T_{em}+M_{mn}{\left[\left|u\right|\right]}_{n}\\ \mathrm{Calculate}\;\sigma : & \; & & \; & & \sigma =C\varepsilon =C\left(Bd+G_{sb}\hat{n}{\left[\left|u\right|\right]}_{n}\right)\\ \mathrm{Determine}\;\sigma_{tt},\sigma_{tm},\sigma_{mm}: & \; & & \; & & \sigma_{tt}={\hat{t}}^{T}\bar{\bar{\sigma }}\;\hat{t},\;\sigma_{tm}={\hat{t}}^{T}\bar{\bar{\sigma }}\;\hat{m}\\ \; & \; & & \; & & \sigma_{mm}={\hat{m}}^{T}\bar{\bar{\sigma }}\;\hat{m} \end{array} \end{array}$$

(90) $$\begin{array}{c} \underline{\mathrm{Three modes formulation approach definition}}\\ \begin{array}{cccccc} \mathrm{Kinematic}\;\mathrm{modes}: & \; & & \; & & \;{\left[\left|u\right|\right]}_{n},{\left[\left|u\right|\right]}_{t},{\left[\left|u\right|\right]}_{m}\\ \mathrm{Linear},\;\mathrm{known}\;\varphi : & \; & & \; & & \varphi =\alpha_{0}+\alpha_{01}x+\alpha_{01}y\\ \mathrm{Fixed},\;\mathrm{constant}\;G_{sb}: & \; & & \; & & G_{sb}=-\sum_{i}^{N_{e}}p_{i}B_{i}\\ \mathrm{Traction}-\mathrm{separation}\;\mathrm{system}↘ & \; & & \; & & \\ \mathrm{Solve}\;\mathrm{for}\;{\left[\left|u\right|\right]}_{n},{\left[\left|u\right|\right]}_{t},{\left[\left|u\right|\right]}_{m}: & \; & & \; & & T=T_{e}+M{\left[\left|u\right|\right]}_{\hat{n}}=q\left({\left[\left|u\right|\right]}_{\hat{n}}\right)\\ \mathrm{Calculate}\;T_{n},T_{t},T_{m}↗ & \; & & \; & & \\ \mathrm{Calculate}\;\sigma : & \; & & \; & & \sigma =C\varepsilon =C\left(Bd+G_{sb}R{\left[\left|u\right|\right]}_{\hat{n}}\right)\\ \mathrm{Determine}\;\sigma_{tt},\sigma_{tm},\sigma_{mm}: & \; & & \; & & \sigma_{tt}={\hat{t}}^{T}\bar{\bar{\sigma }}\;\hat{t},\;\sigma_{tm}={\hat{t}}^{T}\bar{\bar{\sigma }}\;\hat{m}\\ \; & \; & & \; & & \sigma_{mm}={\hat{m}}^{T}\bar{\bar{\sigma }}\;\hat{m} \end{array} \end{array}$$

Once α is solved, the element internal solution process continues as usual, solving the three-variable system and calculating the fracture traction vector. The complete fracture stress σ has to be calculated using a volume average (Equation (21)) as $G_{sb}$ is no longer constant in space. Please refer to Equation (91).

(91) $$\begin{array}{c} \underline{\mathrm{Three modes formulation, controlled}\;\varphi }\\ \begin{array}{cccccc} \mathrm{Kinematic}\;\mathrm{modes}: & \; & & \; & & \;{\left[\left|u\right|\right]}_{n},{\left[\left|u\right|\right]}_{t},{\left[\left|u\right|\right]}_{m}\\ \mathrm{Quadratic},\;\mathrm{unknown}\;\mathrm{single}\;\varphi : & \; & & \; & & \varphi =P_{2}^{T}\alpha \\ \mathrm{Linear}\;G_{sb},\;\mathrm{function}\;\mathrm{of}\;\alpha : & \; & & \; & & G_{sb}=-\sum_{i}^{N_{e}}p_{i}\nabla \varphi \left(\alpha ,x,y,z\right)\\ \mathrm{Solution}\;\mathrm{for}\;\alpha \;\mathrm{coefficients}: & \; & & \; & & \varphi \left(x_{i},\alpha \right)=p_{i},\;M_{i\neq j}\left(\alpha \right)=0\\ \; & \; & & \; & & M_{nn}\left(\alpha \right)=M_{nn_{\infty }}\\ \mathrm{Traction}–\mathrm{separation}\;\mathrm{system}↘ & \; & & \; & & \\ \mathrm{Solve}\;\mathrm{for}\;{\left[\left|u\right|\right]}_{n},{\left[\left|u\right|\right]}_{t},{\left[\left|u\right|\right]}_{m}: & \; & & \; & & T=T_{e}+M{\left[\left|u\right|\right]}_{\hat{n}}=q\left({\left[\left|u\right|\right]}_{\hat{n}}\right)\\ \mathrm{Calculate}\;T_{n},T_{t},T_{m}↗ & \; & & \; & & \\ \mathrm{Calculate}\;\sigma : & \; & & \; & & \sigma =\frac{1}{V_{e}}\int C\left[Bd+G_{sb}R{\left[\left|u\right|\right]}_{\hat{n}}\right]dV\\ \mathrm{Determine}\;\sigma_{tt},\sigma_{tm},\sigma_{mm}: & \; & & \; & & \sigma_{tt}={\hat{t}}^{T}\bar{\bar{\sigma }}\;\hat{t},\;\sigma_{tm}={\hat{t}}^{T}\bar{\bar{\sigma }}\;\hat{m}\\ \; & \; & & \; & & \sigma_{mm}={\hat{m}}^{T}\bar{\bar{\sigma }}\;\hat{m} \end{array} \end{array}$$

### 6.3. Enriched Kinematics Formulation

The last formulation type uses full enriched fracture kinematics as described in Section 4.3 and Section 5.2 through a generalised mode vector ξ:

$$\xi ={\left[\begin{array}{ccccccccc} {\left[\left|u\right|\right]}_{n0} & {\left[\left|u\right|\right]}_{t0} & {\left[\left|u\right|\right]}_{m0} & \theta_{n} & \theta_{t} & \theta_{m} & \epsilon_{n} & \epsilon_{t} & \epsilon_{m} \end{array}\right]}^{t},$$

counting three rigid body displacements, three rigid body rotations and three simple axial strains. Once again, two different models from this family are included in the present analysis: one considering a fixed definition for φ and the other a more complex, parameterised one.

For a fixed φ approach, the idea is to numerically define the $\bar{G_{sb}^{\prime \pm }}$ operators by calculating each column corresponding to each of the modes $\xi_{k}$ using kinematic mode linking relations. The process was already discussed in Section 4.3 for 2D as originally conceived in [11]. The same idea is brought now to the current three-dimensional problem. The specific expressions for each of the columns for $\bar{G_{sb}^{\prime \pm }}$ are explicitly detailed in Appendix C, as well as all the rationale behind the kinematic mode linking in a 3D context. There is no need to look after the original shape for the φ function. This formulation is summarised in Equation (92).

(92) $$\begin{array}{c} \underline{\mathrm{Enriched Modes Formulation, Fixed}\;\varphi }\\ \begin{array}{cccccc} \mathrm{Kinematic}\;\mathrm{modes}: & \; & & \; & & \;{\left[\left|u\right|\right]}_{n_{0}},{\left[\left|u\right|\right]}_{t_{0}},{\left[\left|u\right|\right]}_{m_{0}}\\ \; & \; & & \; & & \theta_{n},\theta_{t},\theta_{m}\;;\;\epsilon_{n},\epsilon_{t},\epsilon_{m}\\ \mathrm{Fixed}\;(\mathrm{unknown}\;\mathrm{shape})\;\varphi : & \; & & \; & & \mathrm{Not}\;\mathrm{used}\;\mathrm{explicitly}\\ \mathrm{Fixed}\;\mathrm{columns}\;\mathrm{for}\;\bar{G_{sb}^{\prime \pm }}: & \; & & \; & & {\bar{G_{sb}^{\prime \pm }}}_{k}\xi_{k}=-Bd_{k}\\ \mathrm{Traction}–\mathrm{separation}\;\mathrm{system}: & \; & & \; & & T_{n}=T_{en}+\sum M_{n_{nk}}\xi_{k}=q_{nn}\left(\xi \right)\\ \; & \; & & \; & & T_{t}=T_{et}+\sum M_{n_{tk}}\xi_{k}=q_{nt}\left(\xi \right)\\ \; & \; & & \; & & T_{m}=T_{em}+\sum M_{n_{mk}}\xi_{k}=q_{nm}\left(\xi \right)\\ \mathrm{Complementary}\;\mathrm{damage}: & \; & & \; & & \sigma_{tt}=T_{tt}+\sum M_{t_{tk}}\xi_{k}=q_{tt}\left(\xi \right)\\ \; & \; & & \; & & \sigma_{tm}=T_{tm}+\sum M_{t_{mk}}\xi_{k}=q_{tm}\left(\xi \right)\\ \; & \; & & \; & & \sigma_{mm}=T_{mm}+\sum M_{m_{mk}}\xi_{k}=q_{mm}\left(\xi \right)\\ \mathrm{Closing}\;\mathrm{constraints}: & \; & & \; & & \sum A_{lk}\xi_{k}=B_{l},\;l=1,2,3\\ \mathrm{Solve}\;\mathrm{for}\;\xi & \; & & \; & & ↘\\ \mathrm{Calculate}\;T_{n},T_{t},T_{m} & \; & & \; & & \to \;\mathrm{From}\;\mathrm{the}\;\mathrm{set}\;\mathrm{of}\;9\;\mathrm{equations}\;\mathrm{just}\;\mathrm{above}\\ \mathrm{Calculate}\;\sigma_{tt},\sigma_{tm},\sigma_{mm} & \; & & \; & & ↗ \end{array} \end{array}$$

Once the $\bar{G_{sb}^{\prime \pm }}$ operator is populated, the approach follows the ideas proposed in Section 5.4 for building the three equation system depicted in Equation (71) for the traction vector components $T_{n},T_{n},T_{n}$. Three additional equations for damaging the fracture stress components $\sigma_{tt},\sigma_{tt},\sigma_{tt}$ are built as well following the structure in Equation (74). The last required three equations are taken from Equation (85), which help to uncouple fracture translations completely. Again, it is remarked that these last three auxiliary equations are proposed to close the system completely and to further help giving a physical sense to the kinematic modes $\xi_{k}$, using some of them as buffer variables. At the end, a 9×9 nonlinear system is obtained, regardless of the φ approach. Once the system is solved, all fracture statics variables $\left(T_{n},T_{t},T_{m},\sigma_{tt},\sigma_{tm},\sigma_{mm}\right)$ will be available directly.

The controlled φ version of this model deals with a considerably more complex φ definition compared to that used on the three-mode approach: a full cubic, piece-wise, triple φ function capable of delivering up to 120 free parameters. The $G_{sb}^{\prime \pm }$ operators will be dependent on the α coefficients following the discussion of Section 5.2, and mode linking relations will serve to build linear restrictions on α. The specific structure of the complete linear system on α for this proposal is detailed in Appendix C. It makes a total of 117 linear constraints. An SVD solution approach is once again used to avoid under/over-constraint problems. Once the α system is solved, the static solution process continues exactly the same as mentioned before. The approach is stated in Equation (93).

(93) $$\begin{array}{c} \underline{\mathrm{Enriched Modes Formulation, Controlled}\;\varphi }\\ \begin{array}{cccccc} \mathrm{Kinematic}\;\mathrm{modes}: & \; & & \; & & \;{\left[\left|u\right|\right]}_{n_{0}},{\left[\left|u\right|\right]}_{t_{0}},{\left[\left|u\right|\right]}_{m_{0}}\\ \; & \; & & \; & & \theta_{n},\theta_{t},\theta_{m}\;;\;\epsilon_{n},\epsilon_{t},\epsilon_{m}\\ \mathrm{Cubic},\;\mathrm{Piece-wise},\;\mathrm{triple}\;\varphi : & \; & & \; & & \varphi_{j}^{\pm }=P_{3}^{T}\alpha_{j}^{\pm },\;j=n,t,m\\ \mathrm{Quad}.\;G_{sb}^{\prime \pm },\;\mathrm{function}\;\mathrm{of}\;\alpha : & \; & & \; & & G_{sb}^{\prime \pm }\left(\alpha \right)=H_{\Gamma }\nabla J-\left(\varphi \nabla J\right)-\nabla \varphi J\\ \mathrm{Solution}\;\mathrm{for}\;\alpha \;\mathrm{coefficients}: & \; & & \; & & {\bar{G_{sb}^{\prime \pm }}}_{k}\left(\alpha \right)\xi_{k}=-Bd_{k}\\ \; & \; & & \; & & \varphi \left(x_{i},\alpha \right)=p_{i}\\ \; & \; & & \; & & {\left.\varphi^{+}\left(\alpha \right)\right|}_{\Gamma_{d}}={\left.\varphi^{-}\left(\alpha \right)\right|}_{\Gamma_{d}}\\ \; & \; & & \; & & M_{i\neq j}\left(\alpha \right)=0,\;i,j=n,t,m\\ \; & \; & & \; & & M_{nn}\left(\alpha \right)=M_{nn_{\infty }},M_{tt}\left(\alpha \right)=M_{tt_{\infty }}\\ \; & \; & & \; & & M_{mm}\left(\alpha \right)=M_{mm_{\infty }}\\ \mathrm{Traction}-\mathrm{separation}\;\mathrm{system}: & \; & & \; & & T_{n}=T_{en}+\sum M_{n_{nk}}\xi_{k}=q_{nn}\left(\xi \right)\\ \; & \; & & \; & & T_{t}=T_{et}+\sum M_{n_{tk}}\xi_{k}=q_{nt}\left(\xi \right)\\ \; & \; & & \; & & T_{m}=T_{em}+\sum M_{n_{mk}}\xi_{k}=q_{nm}\left(\xi \right)\\ \mathrm{Complementary}\;\mathrm{damage}: & \; & & \; & & \sigma_{tt}=T_{tt}+\sum M_{tt_{i}}\xi_{i}=q_{tt}\left(\xi \right)\\ \; & \; & & \; & & \sigma_{tm}=T_{tm}+\sum M_{tm_{i}}\xi_{i}=q_{tm}\left(\xi \right)\\ \; & \; & & \; & & \sigma_{mm}=T_{mm}+\sum M_{mm_{i}}\xi_{i}=q_{mm}\left(\xi \right)\\ \mathrm{Closing}\;\mathrm{constraints}: & \; & & \; & & \sum A_{lk}\xi_{k}=B_{l},\;l=1,2,3\\ \mathrm{Solve}\;\mathrm{for}\;\xi & \; & & \; & & ↘\\ \mathrm{Calculate}\;T_{n},T_{t},T_{m} & \; & & \; & & \to \;\mathrm{From}\;\mathrm{the}\;\mathrm{set}\;\mathrm{of}\;9\;\mathrm{equations}\;\mathrm{just}\;\mathrm{above}\\ \mathrm{Calculate}\;\sigma_{tt},\sigma_{tm},\sigma_{mm} & \; & & \; & & ↗ \end{array} \end{array}$$

### 6.4. Results and Discussion

A series of plots is presented next to make a direct comparison of the results obtained from the elemental simulations for the five formulations types. The results are divided into two sections: static analysis and kinematic analysis. The static analysis pertains the results of all fracture traction vector components $T_{n},T_{t},T_{m}$ and the fracture stress state components $\sigma_{tt},\sigma_{tm},\sigma_{mm}$. The kinematic analysis concerns the behaviour of the pertinent kinematic modes (one for the single formulation, three for the full translation and nine for the completely enriched formulations) and their comparison to the kinematic reference already defined at the beginning of this section.

#### 6.4.1. Static Results

**Figure 15:**
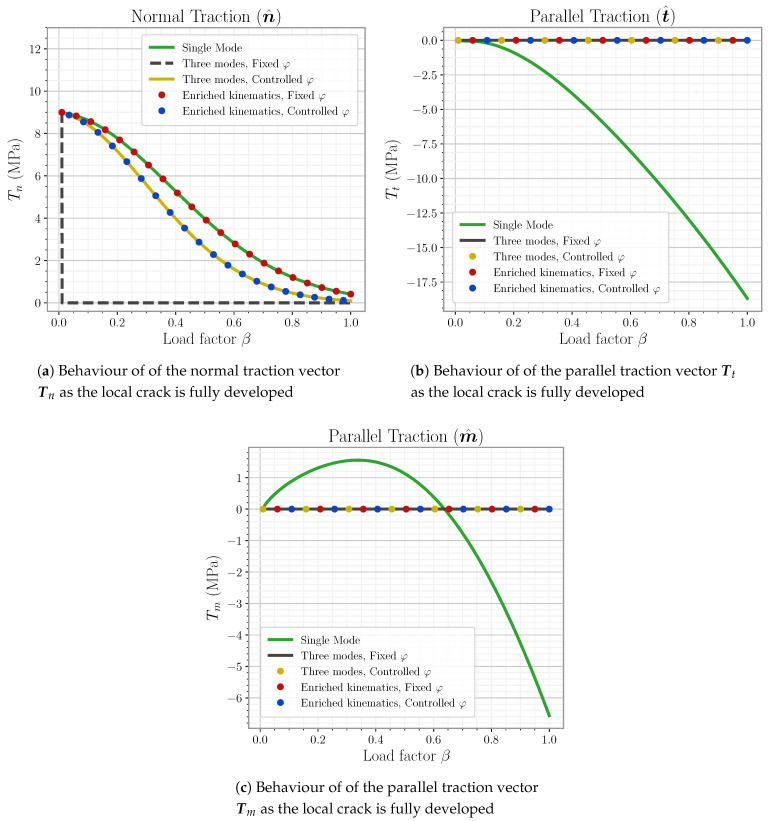
Comparison of the fracture traction components response for all five formulations. In (**a**), the behaviour of the normal traction component $T_{n}$ is shown, while (**b**) and (**c**) show the parallel components $T_{t}$ and $T_{m}$, respectively.

The load profile is conceived with enough crack separation and sliding for placing the element at terminal separation conditions. The $\Omega^{+}$ and the $\Omega^{-}$ regions thus act as completely independent bodies, and no forces shall be exerted from one body to the other at all. This means that all fracture traction vector components $T_{n},T_{t},T_{m}$ and all the remaining stress state components associated with the fracture interface $\sigma_{tt},\sigma_{tm},\sigma_{mm}$ should be zero at this point. The expected behaviour for a good E-FEM formulation would be then to drive all these static components to zero at the end of the load profile. That is, the following plots evidence the damaging quality of the formulations. Figure 15 shows the behaviour of the traction vector components $T_{n}$, $T_{t}$, $T_{m}$ as a function of the load factor β. All formulations manage to damage $T_{n}$ completely: this is the most basic functionality of any E-FEM-based model. Generally speaking, the decaying rate depends on the fracture stiffness component $M_{nn}$, with the only exception of the three-mode, fixed φ formulation. The value of $M_{nn}$ will differ slightly for formulations where normal separation consistency has been ensured through φ coefficients (Equation (59)). The three-mode, fixed φ approach will have an *effective* normal fracture stiffness that will be the resulting effect of combining $M_{nn}$ as well as coupled stiffness terms $M_{nt}$, $M_{nm}$, which will produce for this case a very low effective normal stiffness, so that the traction $T_{n}$ is driven down immediately after starting the load application.

For $T_{t}$ and $T_{m}$, all formulations managing at least three kinematic modes are capable of successfully damaging these parallel traction components. These already start at zero since their localisation states $\sigma_{ynt},\sigma_{ynm}$ have begun at zero attending to the Rankine criterion assumed before. The task for the formulations is just to keep these traction components stabilised at zero. The plots only point out the fact that a single mode formulation will never be able to do so. Despite this fact, single mode calculations remain conveniently simple and these stress anomalies can be manually suppressed to continue with global nonlinear calculations in a large scale model. This is the approach followed by [22,24,33,34]. Even with its partial loss of physical meaning at the local level, its numerical robustness and fracture representation capabilities at large scale models have proven to be successful.

**Figure 16:**
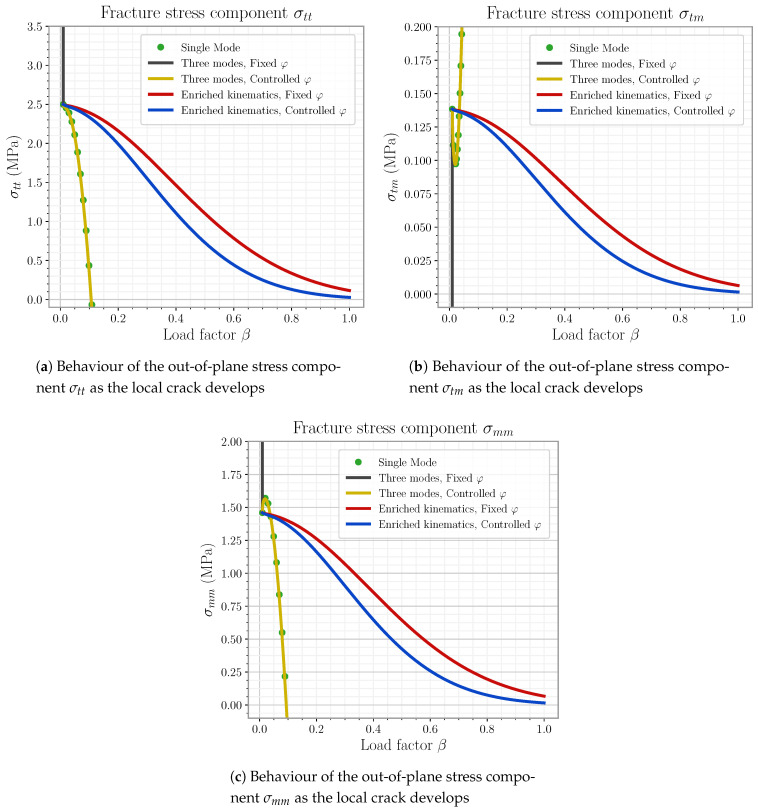
Comparison of the response for stress state components (**a**) $\sigma_{tt}$, (**b**) $\sigma_{tm}$ and (**c**) $\sigma_{mm}$ as the local crack kinematics develops completely for all five formulations currently studied.

Figure 16 shows the behaviour of the stress state components $\sigma_{tt}$, $\sigma_{tm}$ and $\sigma_{mm}$. Fracture stress components are only accessible when projecting the complete state σ to the $\hat{t}$ and $\hat{m}$ directions. Only the enriched mode formulations are designed to explicitly track these components and to damage them properly. They do so at different rates, again, due to φ implications on fracture stiffness. All remaining formulations simply make these stress components grow without control, rendering the need for manual suppression necessary for avoiding stress locking problems.

Based on this static results analysis, only enriched mode formulations exhibit proper damaging properties for this realistic element.

#### 6.4.2. Kinematic Results

For the kinematic consistency analysis, we already have the kinematic reference on which the load design was based. The following plots will show how the formulations manage to follow this reference for each kinematic mode $\xi_{k}$, knowing beforehand that the global approach taken for all formulations possesses mathematical ambiguities that will always prevent a perfect match (Section 4.3).

**Figure 17:**
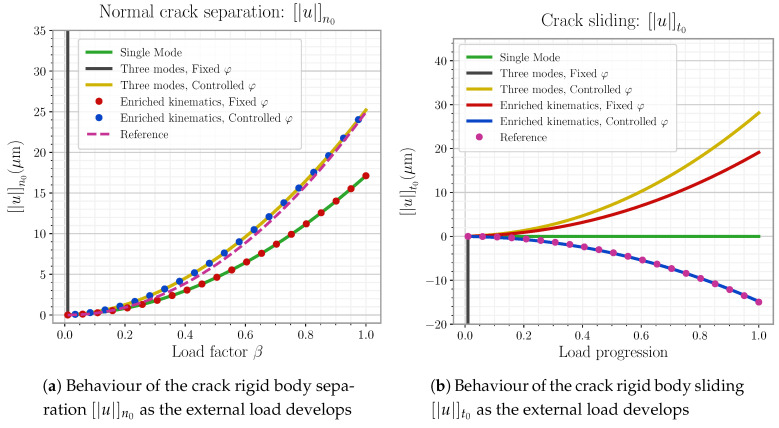

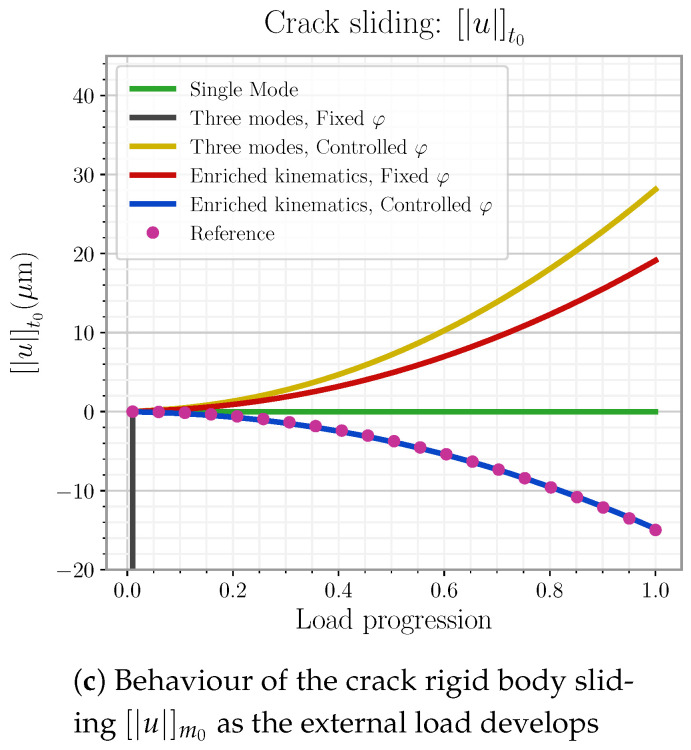
Comparison of the response for fracture rigid body displacements for all five formulations, having (**a**) normal crack separation ${\left[|u|\right]}_{n_{0}}$, (**b**) and (**c**) as parallel sliding ${\left[|u|\right]}_{t_{0}}$ and ${\left[|u|\right]}_{m_{0}}$, respectively. The single mode formulation has been set at zero for both sliding modes.

Figure 17 shows the analysis of rigid body displacements. The normal crack separation ${\left[\left|u\right|\right]}_{n_{0}}$ plot is the one still having all five formulations with correct predictions at once. The formulations having a controlled φ will be naturally closer to the kinematic reference as the fracture stiffness has been designed to be consistent with normal displacement at terminal separation conditions. The other two having slightly different $M_{nn}$ miss the target by 30% but still retain the same behaviour tendency. Again, all formulations were prepared, one way or another, to handle normal separation kinematics. The three-mode, fixed φ again, has a very different effective normal stiffness, which causes even the first load step solution for ${\left[\left|u\right|\right]}_{n_{0}}$ to be excessively large.

The lack of kinematic consistency in the models starts to be truly appreciated in the analysis of crack sliding modes ${\left[\left|u\right|\right]}_{t_{0}},{\left[\left|u\right|\right]}_{m_{0}}$. In this case, there is only one single approach that achieves the correct the kinematic reference: the enhanced kinematics with controlled φ. This is because this approach is the only one with a definition for φ complex enough to allow free parameters to help impose terminal separation conditions on the fracture sliding stiffness terms $M_{tt},M_{mm}$. It is important to note that, while the three-mode, controlled φ attempts to do the same, its limited φ structure does not brake the linear dependency observed on the diagonal of the M matrix. In this case, only one direction can be made to comply with terminal separation conditions, and it was chosen to be $\hat{n}$. The three-mode fixed φ approach, once again, has mixed stiffness interactions, which drive the effective sliding stiffness to unrealistic levels in both directions.

**Figure 18:**
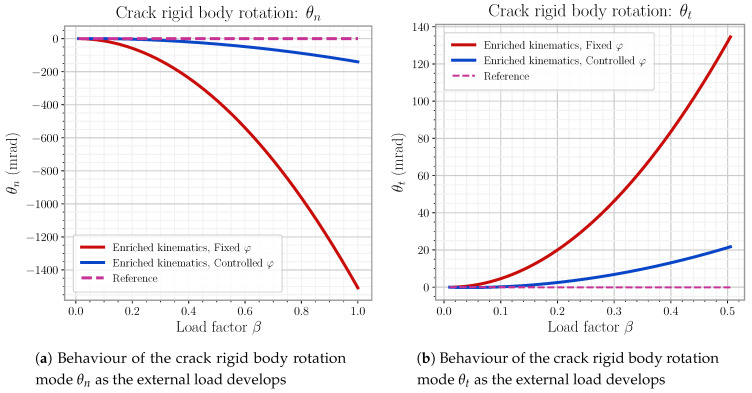

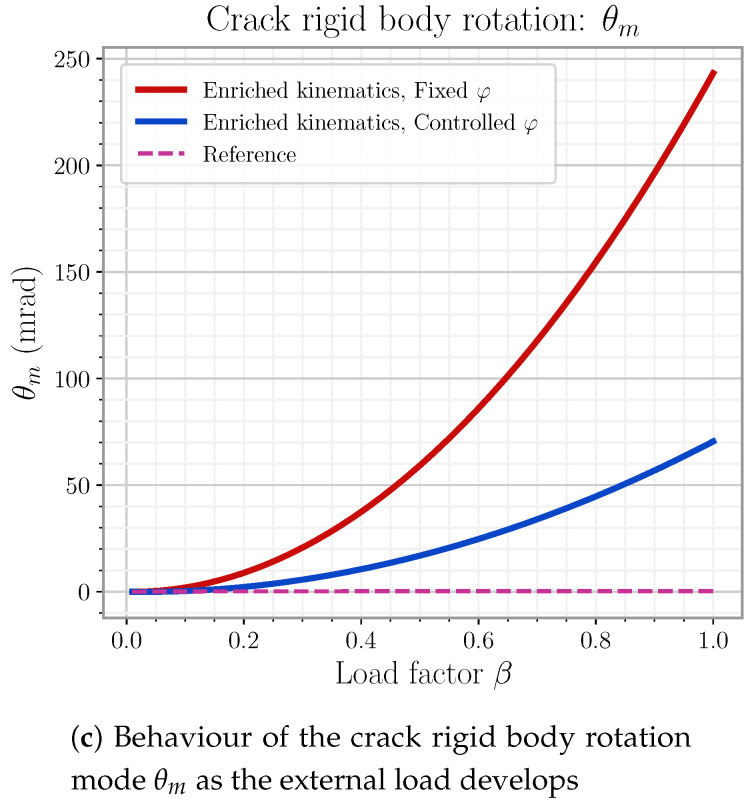
Comparison of the response of fracture rigid body rotations for the enriched modes formulations, having (**a**) rigid body rotation $\theta_{n}$, (**b**) rotation $\theta_{t}$ and (**c**) rotation $\theta_{m}$. The other formulations do not count with such kinematic modes.

Formulations not being able to control sliding fracture stiffness will even predict a completely opposite sliding direction to that demanded by the load vector d. This totally breaks the physical sense of these kinematic modes. It is interesting to note that, if the zero response from the single mode formulation was included, it would still be missing the kinematic target *by less* than the other formulations. In some conditions, the lack of response is indeed a good response, after all. Figure 18 shows the analysis on rigid body rotations. From now on, only the last two formulations involving enriched modes are able to contend. The kinematic reference remains low compared to the displacement demands, so that the task for the formulations is basically to keep the response as low as possible. This is related to the fact that rotation modes, as well as axial strain modes, are mainly conceived as *buffer* variables that help kinematic consistency efforts to focus on fracture displacement modes.

In all cases, both formulations follow the same rotation tendency, having the controlled φ version exhibit a considerably lower response. The torsion $\theta_{n}$ presents the most notable deviations, with almost $83^{\circ }$ for the case of a fixed φ and $8^{\circ }$ for a controlled one.

**Figure 19:**
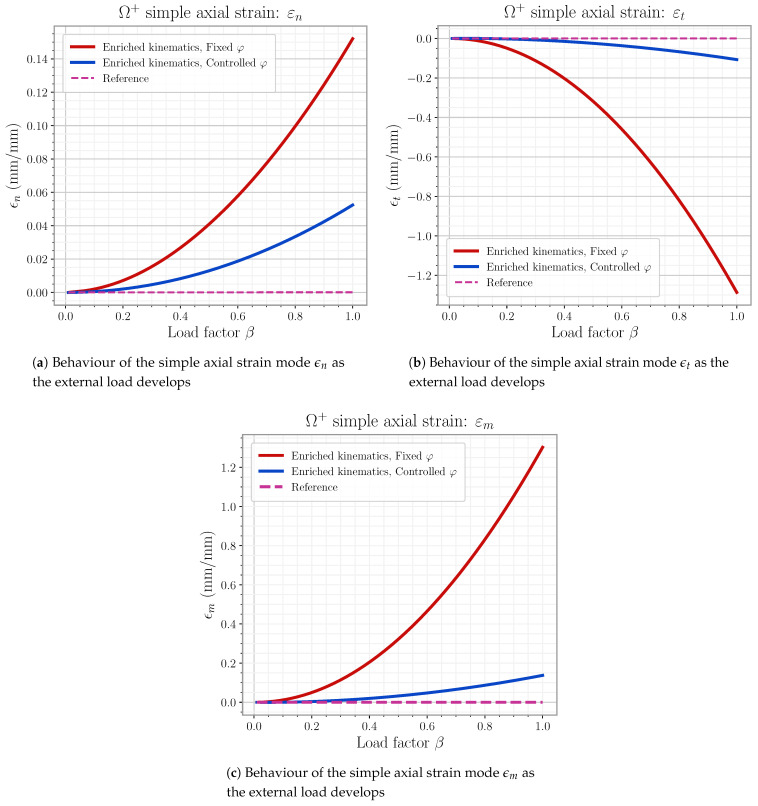
Comparison of the response of the simple axial strain modes associated with the $\Omega^{+}$ body partition: (**a**) axial strain $\epsilon_{n}$, (**b**) axial strain $\epsilon_{t}$ and (**c**) axial strain $\epsilon_{m}$. Only for enriched mode formulations.

Finally, the same trend is observed with the simple axial strains, as shown in Figure 19. The fixed φ approach presents excessively high values for lateral strains. No formulation is able to contain the strain response at zero, but the results suggest the controlled φ formulation fairs better at controlling this buffer kinematic mode.

To better explain the tendency of results in both Figure 18 and Figure 19, it is worth noting that the exponential decay proposed (Equation (87)) for damaging all traction vector and stress components related to the local fracture only involves the rigid body translation fracture kinematic modes ${\left[\left|u\right|\right]}_{n_{0}},{\left[\left|u\right|\right]}_{t_{0}},{\left[\left|u\right|\right]}_{m_{0}}$. This implies that, when the local fracture develops, the normal separation and parallel sliding modes are the ones dominating the development and the final onset of the element segmentation. Thus, it is also implied that rotation and simple axial strain modes $\left(\theta_{n},\theta_{t},\theta_{m},\epsilon_{n},\epsilon_{t},\epsilon_{m}\right)$ will not have the same degree of influence on the overall fracture process and that the formulation as it is intends not to involve them during this part of the solution for all local element variables. This is a very particular choice made by the authors in this work, and a different choice could be made.

Such choice will sacrifice some of the capability to achieve kinematic consistency for rigid body rotations and simple axial strains up to a certain degree. In the case of the fixed and controlled φ formulations portrayed in Figure 18 and Figure 19, it is seen that both have the natural tendency to diverge from low values as the external load is increased. However, the most complex formulation is able to deal better with such partial loss of robustness and is rendered acceptable by the authors of this work. Thus, this choice allows for a reasonable compromise between obtaining near perfect kinematic consistency for an entire 3D E-FEM framework and keeping it mathematically manageable as to make a large-scale implementation process as efficient and less error-prone as possible. As the analysis in previous cases, a single mode formulation would technically obtain the correct kinematic consistency, as having these modes by default as non-existent implies a zero value for them and therefore a much closer match with respect to the kinematic reference in Figure 18 and Figure 19. However, it should be kept in mind that single mode formulations come with the inconvenience of inherent static inconsistency, and they will not function properly without a deliberate element erosion process. The latest generation of E-FEM formulations avoids such undesired traits.

## 7. Conclusions

An extensive study has been presented on the E-FEM strong discontinuity analysis framework, stating a solid basis of its fundamentals and discussing the most common recent formulation approaches. This base has helped to analyse general flaws observed in current formulations and to propose reasonable options for evolving the framework. Many authors had already hinted at some improvements. This work has consolidated this knowledge, added some enhancements and conformed a generalised working proposal in three-dimensional models.

A realistic test element has been taken as a reference to compare the performance of all described formulations. Simulations at this level have been performed considering a prescription of fracture kinematic modes just right after localisation, producing a nodal displacement profile. This load has been designed to make the element reach terminal separation conditions. A comparison of all responses allowed to study relevant behaviours on fracture kinematics and statics.

As more complexity is introduced in the formulations, an increase in consistency is perceived in both the kinematic and static outputs of the element. The most elaborate proposal (fully enriched kinematics with explicit φ parameters) is the only one to report a relatively clean and sound fracture kinematics vector in its entirety. However, its mathematical complexity and computational cost would raise questions on its practicality and ease of implementation. For the case developed in this study, this last formulation requires solving (only once) a linear system of 120 equations to finish the definition of the structure of the enhancement functions. The previous formulation following in degree of complexity (fully enriched kinematics, direct φ) does not require solving such system at all. Still, it is able to satisfactorily remove all relevant fracture stresses, avoiding all numerical locking nuisances observed with other formulations.

Is it really worth adding this much complexity to the formulations just for the sake of obtaining cleaner fracture statics and kinematics? If it is desired to use the fracture kinematics information for more advanced modelling of internal fracture phenomena such as frictional sliding, fault reclosure and the effects of compression, then having these outputs correct becomes important. These complex calculations will rely on having a more realistic state of how the fracture moves. On the other hand, if the modelling goal is just to have a general idea of how a global fracture pattern starts to develop in a model, an approximate path and an estimation of a global strength, the fixed φ approaches will suffice.

In all cases, while none of the formulations are perfectly robust and mechanically correct, it remains satisfactory to see that the E-FEM framework can be taken this far for the modelling of strong discontinuities, gaining a lot in physical meaningfulness and still retaining the charms that normally attract many computational mechanics researchers.

## Data Availability

Data available from authors.

## Abbreviations

For the ease of reading through all theoretical developments in this work, a supplementary symbol list involving the most significant variables is provided to the reader attending to the different parts of the E-FEM strong discontinuity framework. The following abbreviations are used in this manuscript:

**Basic kinematics:**	
u	Displacement field vector
$\Gamma_{d}$	Discontinuity (local fracture) surface
$\Omega^{+}$	“+” domain of the local fracture element partitions
$\Omega^{-}$	“−” domain of the local fracture element partitions
$H_{\Gamma_{d}}$	Heaviside function having $\Gamma_{d}$ as a positional trigger
$\left[\left|u\right|\right]$	Displacement jump vector
$\hat{u}$	Discontinuity-integrated displacement vector
φ	Auxiliary function aiding the construction of $\hat{u}$, scalar version
φ	Compound, three-component definition of φ
$\delta_{\Gamma }$	Dirac delta having $\Gamma_{d}$ as positional trigger
$\hat{n}$	Normal vector to the local fracture surface $\Gamma_{d}$
$\hat{t}$	First parallel vector to the local fracture surface $\Gamma_{d}$
$\hat{m}$	Second parallel vector to the local fracture surface $\Gamma_{d}$
**Variational Analysis:**	
ε	Strain field vector (real)
δε	Strain field vector variation (virtual)
σ	Stress field vector (real)
δσ	Stress field vector variation (virtual)
$\sigma \left(\varepsilon \right)$	Stress field calculated via constitutive relations based on real strains
C	Linear elastic constitutive matrix
d	Nodal displacement vector
δd	Nodal displacement vector variation (virtual)
$\delta \left[\left|u\right|\right]$	Displacement jump variation (virtual)
N	Standard finite element displacement interpolation matrix
B	Standard finite element strain interpolation matrix
$G_{s}$	Strong discontinuity matrix operator
$G_{sb}$	Bounded section of the operator $G_{s}$
$G_{su}$	Unbounded section of the operator $G_{s}$
$G_{s}^{*}$	Strong discontinuity **virtual** matrix operator
$G_{sb}^{*}$	Bounded section of the operator $G_{s}^{*}$
$G_{su}^{*}$	Unbounded section of the operator $G_{s}^{*}$
s	Nodal stress vector
δs	Nodal stress vector variation (virtual)
S	Real stress interpolation matrix
$S^{*}$	Virtual stress interpolation matrix
$H_{s}$	Projection matrix operator ($\hat{n}$ direction)
$V_{e}$	Elemental volume
$V^{+}$	Volume of the $\Omega^{+}$ domain
$V^{-}$	Volume of the $\Omega^{-}$ domain
$A_{\Gamma }$	Area of local fracture surface $\Gamma_{d}$
T	Local fracture surface traction vector
$T_{e}$	Nodal displacement-driven part of surface traction vector T
M	Crack stiffness matrix, derived from projections on $\hat{n}$
${\left[\left|u\right|\right]}_{\hat{n}}$	Displacement jump vector in the local fracture surface frame
**Advanced pathology analysis and new proposals:**	
R	Rotation matrix onto the local $\left(\hat{n},\hat{t},\hat{m}\right)$ frame
ξ	Generalised fracture kinematics vector
${\left[\left|u\right|\right]}_{j}$	Fracture rigid body translation modes ($j=\hat{n},\hat{t},\hat{m}$)
$\theta_{j}$	Fracture rigid body rotation modes ($j=\hat{n},\hat{t},\hat{m}$)
$\epsilon_{j}$	Fracture simple axial strain modes ($j=\hat{n},\hat{t},\hat{m}$)
J	Generalised fracture kinematics interpolation matrix
$G_{sb}^{\prime }$	Compound strong discontinuity involving the J operator
$G_{su}^{\prime }$	Compound strong discontinuity involving the J operator
$\bar{G_{sb}^{\prime \pm }}$	Volume-averaged $G_{sb}^{\prime }$ operator through $V^{+}$ or $V^{-}$
$d_{k}$	Nodal displacement state associated with the fracture kinematic mode $\xi_{k}$
$p_{i}$	Binary indicator for element domains
α	Polynomial coefficients for a compound φ definition
ξ,η,ζ	Local coordinates in the $\left(\hat{n},\hat{t},\hat{m}\right)$ fracture frame
$M_{jj_{\infty }}$	Fracture stiffness in terminal separation conditions for the *j* direction
$H_{st}$	Projection matrix operator ($\hat{t}$ direction)
$H_{sm}$	Projection matrix operator ($\hat{m}$ direction)
$M_{t}$	Crack stiffness matrix, derived from projections on $\hat{t}$
$M_{m}$	Crack stiffness matrix, derived from projections on $\hat{m}$

## Appendix A. Derivation of an Explicit Crack Stiffness Matrix M

The final expression for the crack stiffness matrix M shown in Equation (40) may appear surprisingly compact considering its origin in Equation (38). This appendix will clear out the derivation process for the case of a 1-3 node partition on $\Omega^{+},\Omega^{-}$. Other cases can be expressed as linear superpositions of this one.

Isolating an expression for M we have:

(A1) $$M=\frac{1}{V_{e}}R^{T}\int_{\Omega }H_{s}^{T}CG_{sb}\;dV\;R=R^{T}H_{s}^{T}CG_{sb}R,$$

which assumes a constant $G_{sb}$. The matrix C, considering a linear elastic constitutive law, can be expressed as:

(A2) $$C=\left[\begin{array}{cccccc} c_{n1} & c_{n2} & c_{n2} & 0 & 0 & 0\\ c_{n2} & c_{n1} & c_{n2} & 0 & 0 & 0\\ c_{n2} & c_{n2} & c_{n1} & 0 & 0 & 0\\ 0 & 0 & 0 & c_{s} & 0 & 0\\ 0 & 0 & 0 & 0 & c_{s} & 0\\ 0 & 0 & 0 & 0 & 0 & c_{s} \end{array}\right],$$

where:

(A3) $$\begin{array}{cc} c_{n1} & =\frac{E}{1+\nu }\left(\frac{1-\nu }{1-2\nu }\right) \end{array}$$

(A4) $$\begin{array}{cc} c_{n2} & =\frac{E}{1+\nu }\left(\frac{\nu }{1-2\nu }\right) \end{array}$$

(A5) $$\begin{array}{cc} c_{s} & =\frac{E}{2\left(1+\nu \right)} \end{array}$$

It is not hard to see that these coefficients entail the following relation between them:

(A6) $$c_{n1}-c_{n2}=2c_{s}$$

Recalling the definitions for $H_{s}$ from Equation (16) and for $G_{sb}$ from Equations (36) and (37), the matrix multiplication in Equation (A1) can be developed. The approach is to explicitly devise a couple of coefficients $M_{ij}$ to find general simplification rules for the whole M matrix. Taking, for instance, the resulting expression for the element $M_{11}$, one can group terms by the coefficients of the vector components of Ψ: $\Psi_{ix},\Psi_{iy},\Psi_{iz}$. The following is obtained:

(A7) $$\begin{array}{cc} M_{11}= & \left[c_{n1}n_{x}^{2}+\left(c_{n2}+2c_{s}\right)\left(n_{y}^{2}+n_{z}^{2}\right)\right]n_{x}\Psi_{ix}+\\ \; & \left[c_{n1}n_{y}^{2}+\left(c_{n2}+2c_{s}\right)\left(n_{x}^{2}+n_{z}^{2}\right)\right]n_{y}\Psi_{iy}+\\ \; & \left[c_{n1}n_{x}^{2}+\left(c_{n2}+2c_{s}\right)\left(n_{y}^{2}+n_{z}^{2}\right)\right]n_{x}\Psi_{ix} \end{array}$$

Here, unitary vector properties ($n_{x}^{2}+n_{y}^{2}+n_{z}^{2}=1$) along with Equation (A6) can be used to reach:

(A8) $$M_{11}=c_{n1}\left(n_{x}\Psi_{ix}+n_{y}\Psi_{iy}+n_{z}\Psi_{iz}\right)=c_{n1}{\hat{n}}^{T}\Psi =c_{n1}\hat{n}\cdot \Psi$$

Other $M_{ij}$ can be worked out this way, sometimes making use of orthogonality properties of the ($\hat{n},\hat{t},\hat{m}$) base. Taking as a last example the grouped coefficient for $\Psi_{ix}$ in the expression for $M_{23}$:

(A9) $$\begin{array}{cc} \; & n_{x}t_{x}m_{x}c_{n1}+\left(n_{y}t_{y}+n_{z}t_{z}\right)m_{x}c_{n2}+\left[n_{x}\left(t_{y}m_{y}+t_{z}m_{z}\right)+t_{x}\left(n_{y}m_{y}+n_{z}m_{z}\right)\right]c_{s}=\\ \; & n_{x}t_{x}m_{x}c_{n1}-n_{x}t_{x}m_{x}c_{n2}-2n_{x}t_{x}m_{x}c_{s}=\\ \; & n_{x}t_{x}m_{x}c_{n1}-n_{x}t_{x}m_{x}c_{n2}-n_{x}t_{x}m_{x}\left(c_{n1}-c_{n2}\right)=0, \end{array}$$

where coefficients of $\Psi_{iy}$, $\Psi_{iz}$ follow the same result in a symmetrical fashion.

## Appendix B. Analysis of *φ* Proposal by Wells

In this Appendix, the derivation of Equations (41) and (42) is explained by taking the same 2D triangular element example discussed in Section 4.2.

To fix the crack displacement coupling introduced by means of a fixed definition for φ, Wells [9] proposes a workaround through a redefinition of φ as follows: the vector $\nabla \varphi ={\left[\varphi_{,x},\varphi_{,y}\right]}^{T}$ is rotated to the local frame $\left(\hat{n},\hat{t}\right)$:

(A10) $$\begin{array}{cc} \; & \nabla \varphi =\left[\begin{array}{c} \varphi_{,x}\\ \varphi_{,y} \end{array}\right],\;R=\left[\begin{array}{cc} n_{x} & t_{x}\\ n_{y} & t_{y} \end{array}\right]=\left[\begin{array}{cc} n_{x} & -n_{y}\\ n_{y} & n_{x} \end{array}\right]\\ \; & R^{T}\nabla \varphi =\nabla \varphi_{\hat{n}}=\left[\begin{array}{cc} n_{x} & n_{y}\\ -n_{y} & n_{x} \end{array}\right]\left[\begin{array}{c} \varphi_{,x}\\ \varphi_{,y} \end{array}\right]=\left[\begin{array}{c} n_{x}\varphi_{,x}+n_{y}\varphi_{,y}\\ -n_{y}\varphi_{,x}+n_{x}\varphi_{,y} \end{array}\right], \end{array}$$

where the fact that $\left[t_{x},t_{y}\right]=\left[-n_{y},n_{x}\right]$ for a 2D setting has been used.

Once in the local frame, the $\nabla \varphi_{\hat{n}}$ component in the $\hat{t}$ direction is driven to zero, and this new vector is once again rotated to the global frame:

(A11) $$\begin{array}{cc} \nabla \varphi_{\hat{n}}^{\prime } & =\left[\begin{array}{c} n_{x}\varphi_{,x}+n_{y}\varphi_{,y}\\ 0 \end{array}\right]\\ \nabla \varphi^{\prime } & =\left[\begin{array}{c} \varphi_{,x}^{\prime }\\ \varphi_{,y}^{\prime } \end{array}\right]=R\nabla \varphi_{\hat{n}}^{\prime }=\left[\begin{array}{cc} n_{x} & -n_{y}\\ n_{y} & n_{x} \end{array}\right]\left[\begin{array}{c} n_{x}\varphi_{,x}+n_{y}\varphi_{,y}\\ 0 \end{array}\right]=\left[\begin{array}{c} n_{x}^{2}\varphi_{,x}+n_{x}n_{y}\varphi_{,y}\\ n_{y}^{2}\varphi_{,y}+n_{x}n_{y}\varphi_{,x} \end{array}\right] \end{array}$$

The components of this updated vector become the new definitions for the derivatives $\varphi_{,x},\varphi_{,y}$, thus recovering Equation (41). This would be a sort of equivalent to *forcing* an alignment between element side normals and the fracture normal $\hat{n}$.

The impact of this redefinition on the properties of φ alone can be assessed as follows. Let the bi-dimensional linear φ be expressed as:

(A12) $$\varphi =C+\varphi_{,x}x+\varphi_{,x}y,$$

where *C* is a constant. This φ yields the original $\varphi_{,x},\varphi_{,y}$ when applying a gradient operator. As such, this φ satisfies the basic requirements of Equation (3) by definition. Let us assume a 1-2 node partition, where node 1 belongs to the $\Omega^{-}$ domain. We will naturally have:

(A13) $$\varphi \left(x_{1}\right)=C+\varphi_{,x}x_{1}+\varphi_{,x}y_{1}=0$$

Based on this, the outcome of a new function $\varphi^{\prime }$ built upon $\varphi_{,x}^{\prime },\varphi_{,y}^{\prime }$ under the same conditions can be studied. Its definition will be:

(A14) $$\varphi^{\prime }=C+\varphi_{,x}^{\prime }x+\varphi_{,x}^{\prime }y,$$

If this $\varphi^{\prime }$ were to satisfy the same basic requirements, then we should have $\varphi^{\prime }\left(x_{1}\right)=0$. Solving this and considering Equation (A11) we obtain:

$$\begin{array}{cc} \varphi^{\prime }\left(x_{1}\right) & =C+\varphi_{,x}^{\prime }x_{1}+\varphi_{,x}^{\prime }y_{1}\\ \; & =C+\left(n_{x}^{2}\varphi_{,x}+n_{x}n_{y}\varphi_{,y}\right)x_{1}+\left(n_{y}^{2}\varphi_{,y}+n_{x}n_{y}\varphi_{,x}\right)y_{1}\\ \; & =C+\left[\left(n_{x}^{2}+n_{y}^{2}\right)\varphi_{,x}-n_{y}^{2}\varphi_{,x}+n_{x}n_{y}\varphi_{,y}\right]x_{1}+\left[\left(n_{y}^{2}+n_{x}^{2}\right)\varphi_{,y}-n_{x}^{2}\varphi_{,y}+n_{x}n_{y}\varphi_{,x}\right]y_{1}\\ \; & =\underset{0}{\underset{︸}{C+\varphi_{,x}x_{1}+\varphi_{,x}y_{1}}}+\left(n_{x}n_{y}\varphi_{,y}-n_{y}^{2}\varphi_{,x}\right)x_{1}+\left(n_{x}n_{y}\varphi_{,x}-n_{x}^{2}\varphi_{,y}\right)y_{1}\\ \; & =\left(n_{x}\varphi_{,y}-n_{y}\varphi_{,x}\right)\left(n_{y}x_{1}-n_{x}y_{1}\right)=A\left|\left(\hat{n}\times \Psi_{12}\right)\right|\left|\left(x_{1}\times \hat{n}\right)\right|, \end{array}$$

for some constant *A*. This proves the statement of Equation (42). Similar expressions can be derived for nodes 2 and 3.

## Appendix C. Detailed Description of *φ* Coefficients Linear System Building

In this Appendix, a detailed description of the process for stating a complete strong discontinuity enhancement function definition through an explicit φ in three dimensions is made. We start by proposing a form for φ, for then enunciating each of the mode linking expressions required to start building the linear system to solve for the α coefficients. Then, additional linear constraints related to basic φ properties are described, adding the constraints related to the crack stiffness matrix M as well.

The model works the φ function on a full cubic base $P_{3}$ (20 terms) on the local frame (ξ,η,ζ) (terms described earlier in $P_{2}$ are not shown):

(A15) $$P_{3}={\left[\begin{array}{cccccccccccc} 1 & \cdots & \xi^{2}\eta & \xi \eta^{2} & \eta^{2}\zeta & \eta \zeta^{2} & \xi^{2}\zeta & \xi \zeta^{2} & \xi \eta \zeta & \xi^{3} & \eta^{3} & \zeta^{3} \end{array}\right]}^{T}$$

A separate φ function ($\varphi_{n},\varphi_{t},\varphi_{m}$) is defined for each local direction, and each one is further partitioned in a dual, piece-wise fashion:

(A16) $$\varphi_{n}=\left\{\begin{array}{cc} \varphi_{n}^{+} & \left(\xi ,\eta ,\zeta \right)\in \Omega^{+}\\ \varphi_{n}^{-} & \left(\xi ,\eta ,\zeta \right)\in \Omega^{-} \end{array}\right.\;\varphi_{t}=\left\{\begin{array}{cc} \varphi_{t}^{+} & \left(\xi ,\eta ,\zeta \right)\in \Omega^{+}\\ \varphi_{t}^{-} & \left(\xi ,\eta ,\zeta \right)\in \Omega^{-} \end{array}\right.\;\varphi_{m}=\left\{\begin{array}{cc} \varphi_{m}^{+} & \left(\xi ,\eta ,\zeta \right)\in \Omega^{+}\\ \varphi_{m}^{-} & \left(\xi ,\eta ,\zeta \right)\in \Omega^{-} \end{array}\right.$$

Consequently, different coefficient vectors $\alpha_{j}^{\pm }$ (j=n,m,t) will be assigned to each of the functions $\varphi_{j}^{\pm }$:

(A17) $$\alpha_{j}^{\pm }={\left[\begin{array}{ccccccccccc} \alpha_{j_{0}}^{\pm } & \cdots & \alpha_{j_{122}}^{\pm } & \alpha_{j_{223}}^{\pm } & \alpha_{j_{233}}^{\pm } & \alpha_{j_{113}}^{\pm } & \alpha_{j_{133}}^{\pm } & \alpha_{j_{113}}^{\pm } & \alpha_{j_{111}}^{\pm } & \alpha_{j_{222}}^{\pm } & \alpha_{j_{333}}^{\pm } \end{array}\right]}^{T}$$

Afterwards, the definition of a global coefficient vector α is made having a total of 120 coefficients: 20 (size of the base) times 3 (separate directions) times 2 (piece-wise in $\Omega^{+},\Omega^{-}$). It will be expressed by blocks:

(A18) $$\alpha ={\left[\begin{array}{cccccc} \alpha_{n}^{+} & \alpha_{n}^{-} & \alpha_{t}^{+} & \alpha_{t}^{-} & \alpha_{m}^{+} & \alpha_{m}^{-} \end{array}\right]}^{T}$$

Having cleared a definition for the φ functions, the construction of linear constraints for all α coefficients is described. From now on it will be already implied that the $\varphi_{j}$ functions may take a different form on $\Omega^{+}\left(\varphi_{j}^{+}\right)$ or $\Omega^{-}\left(\varphi_{j}^{-}\right)$, and the signs will be omitted in many of the following equations for the sake of readability.

We start with the kinematic mode linking process, which produces most of the bulk of such constraints. This work is divided in two parts attending Equation (53): devising the left-hand expressions for the α coefficients within each ${\bar{G_{sb}^{\prime \pm }}}_{lk}$ component of $\bar{G_{sb}^{\prime \pm }}$ ($l=1,\cdots ,6;k={\left[\left|u\right|\right]}_{n0},\cdots ,\epsilon_{m}$), and working out the *right-hand* expressions for strains associated with each displacement state $d_{k}$ specific to each excited kinematic mode *k*, so that the linear relations are complete. The left-hand expressions require developing each of the terms of Equation (64b). Recalling the expression for the matrix J found in Equation (63), let $J_{n},J_{t},J_{m}$ be the first, second and third rows, respectively. We now have:

$$\begin{array}{c} G_{sb}^{\prime }=H_{\Gamma }\partial J-\bar{\varphi \partial J}-\partial \varphi J \end{array}$$

(A19) $$\begin{array}{c} H_{\Gamma }\partial J=H_{\Gamma }\left[\begin{array}{c} J_{n,\xi }\\ J_{t,\eta }\\ J_{m,\varsigma }\\ J_{n,\eta }+J_{t,\xi }\\ J_{t,\varsigma }+J_{m,\eta }\\ J_{n,\varsigma }+J_{m,\xi } \end{array}\right]=H_{\Gamma }\left[\begin{array}{ccccccccc} 0 & 0 & 0 & 0 & 0 & 0 & 1 & 0 & 0\\ 0 & 0 & 0 & 0 & 0 & 0 & 0 & 1 & 0\\ 0 & 0 & 0 & 0 & 0 & 0 & 0 & 0 & 1\\ 0 & 0 & 0 & 0 & 0 & 0 & 0 & 0 & 0\\ 0 & 0 & 0 & 0 & 0 & 0 & 0 & 0 & 0\\ 0 & 0 & 0 & 0 & 0 & 0 & 0 & 0 & 0 \end{array}\right] \end{array}$$

(A20) $$\begin{array}{c} \bar{\varphi \partial J}=\left[\begin{array}{c} \varphi_{n}J_{n,\xi }\\ \varphi_{t}J_{t,\eta }\\ \varphi_{m}J_{m,\varsigma }\\ \varphi_{n}J_{n,\eta }+\varphi_{t}J_{t,\xi }\\ \varphi_{t}J_{t,\varsigma }+\varphi_{m}J_{m,\eta }\\ \varphi_{n}J_{n,\varsigma }+\varphi_{m}J_{m,\xi } \end{array}\right]=\left[\begin{array}{ccccccccc} 0 & 0 & 0 & 0 & 0 & 0 & \varphi_{n} & 0 & 0\\ 0 & 0 & 0 & 0 & 0 & 0 & 0 & \varphi_{t} & 0\\ 0 & 0 & 0 & 0 & 0 & 0 & 0 & 0 & \varphi_{m}\\ 0 & 0 & 0 & 0 & 0 & -\varphi_{n}+\varphi_{t} & 0 & 0 & 0\\ 0 & 0 & 0 & -\varphi_{t}+\varphi_{m} & 0 & 0 & 0 & 0 & 0\\ 0 & 0 & 0 & 0 & \varphi_{n}-\varphi_{m} & 0 & 0 & 0 & 0 \end{array}\right] \end{array}$$

(A21) $$\partial \varphi J=\left[\begin{array}{ccc} \varphi_{n,\xi } & 0 & 0\\ 0 & \varphi_{t,\eta } & 0\\ 0 & 0 & \varphi_{m,\varsigma }\\ \varphi_{n,\eta } & \varphi_{t,\xi } & 0\\ 0 & \varphi_{t,\varsigma } & \varphi_{m,\eta }\\ \varphi_{n,\varsigma } & 0 & \varphi_{m,\xi } \end{array}\right]\left[\begin{array}{ccccccccc} 1 & 0 & 0 & 0 & \varsigma & -\eta & \xi & 0 & 0\\ 0 & 1 & 0 & -\varsigma & 0 & \xi & 0 & \eta & 0\\ 0 & 0 & 1 & \eta & -\xi & 0 & 0 & 0 & \varsigma \end{array}\right]$$

It is important to remember that the *∂* operator is the same partial differential operator used to map a displacement interpolation matrix N to a strain operator matrix B in classical FEM theory. For the sake of readability, we present the final expression for $G_{sb}^{\prime }$ in blocks related to kinematic groups.

For the rigid body translation block:

(A22) $$G_{sb_{1-3}}^{\prime }=-\left[\begin{array}{ccc} \varphi_{n,\xi } & 0 & 0\\ 0 & \varphi_{t,\eta } & 0\\ 0 & 0 & \varphi_{m,\varsigma }\\ \varphi_{n,\eta } & \varphi_{t,\xi } & 0\\ 0 & \varphi_{t,\varsigma } & \varphi_{m,\eta }\\ \varphi_{n,\varsigma } & 0 & \varphi_{m,\xi } \end{array}\right]$$

For the rigid body rotation block:

(A23) $$G_{sb_{4-6}}^{\prime }=-\left[\begin{array}{ccc} 0 & \varphi_{n,\xi }\varsigma & -\varphi_{n,\xi }\eta \\ -\varphi_{t,\eta }\varsigma & 0 & \varphi_{t,\eta }\xi \\ \varphi_{m,\varsigma }\eta & -\varphi_{m,\varsigma }\xi & 0\\ -\varphi_{t,\xi }\varsigma & \varphi_{n,\eta }\varsigma & \left[\begin{array}{cc} -\varphi_{n,\eta }\eta & +\varphi_{t,\xi }\xi \\ -\varphi_{n} & +\varphi_{t} \end{array}\right]\\ \left[\begin{array}{cc} -\varphi_{t,\varsigma }\varsigma & +\varphi_{m,\eta }\eta \\ -\varphi_{t} & +\varphi_{m} \end{array}\right] & -\varphi_{m,\eta } & \varphi_{t,\varsigma }\xi \\ \varphi_{m,\xi }\eta & \left[\begin{array}{cc} \varphi_{n,\varsigma }\varsigma & -\varphi_{m,\xi }\xi \\ +\varphi_{n} & -\varphi_{m} \end{array}\right] & -\varphi_{n,\varsigma }\eta \end{array}\right]$$

Finally, for the simple axial strain block:

(A24) $$G_{sb_{7-9}}^{\prime \pm }=-\left[\begin{array}{ccc} \varphi_{n,\xi }\xi +\varphi_{n}-H & 0 & 0\\ 0 & \varphi_{t,\eta }\eta +\varphi_{t}-H & 0\\ 0 & 0 & \varphi_{m,\varsigma }\varsigma +\varphi_{m}-H\\ \varphi_{n,\eta }\xi & \varphi_{t,\xi }\eta & 0\\ 0 & \varphi_{t,\varsigma }\eta & \varphi_{m,\eta }\varsigma \\ \varphi_{n,\varsigma }\xi & 0 & \varphi_{m,\xi }\varsigma \end{array}\right]$$

With this, the complete $G_{sb}^{\prime \pm }$ matrix is integrated at each subvolume to obtain the averaged operators $\bar{G_{sb}^{\prime \pm }}$ as per Equation (65). All populated fields within $\bar{G_{sb}^{\prime \pm }}$ are a function of some of the 120 α coefficients defined so far. Note that, for the case of the simple axial strain block, the Heaviside function H will **not** be a function of α coefficients, since it can only have a value of 1 and zero and it is not multiplied by any function of the φ family. This specific term piece has to be moved to the right-hand expressions when passing to the solution of the complete linear system. With this, the left-hand expression work is completed.

For the right-side expressions, the procedure is similar to that followed in Section 4.3 and proposed by Armero and Linder [11] but generalised to three-dimensions. The key is to find the expression for each displacement state $d_{k}$ associated with each kinematic mode $k={\left[\left|u\right|\right]}_{n0},{\left[\left|u\right|\right]}_{t0},...,\epsilon_{m}$, for then multiplying by B.

For the rigid body displacement modes, the deduction was already explained for 2D and can be easily extrapolated to 3D:

(A25a) $$\begin{array}{c} d_{{\left[\left|u\right|\right]}_{n0}}=\underset{12\times 1}{\underset{︸}{\left[\begin{array}{c} p_{1}{\left[\left|u\right|\right]}_{n0}\hat{n}\\ p_{2}{\left[\left|u\right|\right]}_{n0}\hat{n}\\ p_{3}{\left[\left|u\right|\right]}_{n0}\hat{n}\\ p_{4}{\left[\left|u\right|\right]}_{n0}\hat{n} \end{array}\right]}}=\left[\begin{array}{c} p_{1}\hat{n}\\ p_{2}\hat{n}\\ p_{3}\hat{n}\\ p_{4}\hat{n} \end{array}\right]{\left[\left|u\right|\right]}_{n0} \end{array}$$

(A25b) $$\begin{array}{c} {\bar{G_{sb}^{\prime }}}_{{\left[\left|u\right|\right]}_{n0}}{\left[\left|u\right|\right]}_{n0}=-Bd_{{\left[\left|u\right|\right]}_{n0}} \end{array}$$

$$\begin{array}{c} {\bar{G_{sb}^{\prime }}}_{{\left[\left|u\right|\right]}_{n0}}{\left[\left|u\right|\right]}_{n0}=-\left[\begin{array}{cccc} B_{1} & B_{2} & B_{3} & B_{4} \end{array}\right]\left[\begin{array}{c} p_{1}\hat{n}\\ p_{2}\hat{n}\\ p_{3}\hat{n}\\ p_{4}\hat{n} \end{array}\right]{\left[\left|u\right|\right]}_{n0}=-\sum_{i}^{N_{e}}p_{i}\left(B_{i}\hat{n}\right){\left[\left|u\right|\right]}_{n0} \end{array}$$

(A25c) $$\begin{array}{c} {\bar{G_{sb}^{\prime }}}_{{\left[\left|u\right|\right]}_{n0}}=-\sum_{i}^{N_{e}}p_{i}\left(B_{i}\hat{n}\right) \end{array}$$

${\bar{G_{sb}^{\prime }}}_{{\left[\left|u\right|\right]}_{t0}},{\bar{G_{sb}^{\prime }}}_{{\left[\left|u\right|\right]}_{m0}}$ follow the same way:

(A26) $$\begin{array}{c} {\bar{G_{sb}^{\prime }}}_{{\left[\left|u\right|\right]}_{t0}}=-\sum_{i}^{N_{e}}p_{i}\left(B_{i}\hat{t}\right) \end{array}$$

(A27) $$\begin{array}{c} {\bar{G_{sb}^{\prime }}}_{{\left[\left|u\right|\right]}_{m0}}=-\sum_{i}^{N_{e}}p_{i}\left(B_{i}\hat{m}\right) \end{array}$$

The displacements state $d_{k}$ associated with rigid body rotation modes can be easily found by taking a cross product of the type s=θ×r. The centroid of the fracture surface $x_{0}$ is considered as the centre of rotation to calculate a rotation radius $r_{i}$ to each element node position $x_{i}$, while the rotation itself will have an axis corresponding to each of the local directions:

(A28a) $$\begin{array}{c} d_{\theta_{n}}=\left[\begin{array}{c} p_{1}\left(\left[\theta_{n}\hat{n}\right]\times r_{1}\right)\\ p_{2}\left(\left[\theta_{n}\hat{n}\right]\times r_{2}\right)\\ p_{3}\left(\left[\theta_{n}\hat{n}\right]\times r_{3}\right)\\ p_{4}\left(\left[\theta_{n}\hat{n}\right]\times r_{4}\right) \end{array}\right]=\left[\begin{array}{c} p_{1}\left(\hat{n}\times r_{1}\right)\\ p_{2}\left(\hat{n}\times r_{2}\right)\\ p_{3}\left(\hat{n}\times r_{3}\right)\\ p_{4}\left(\hat{n}\times r_{4}\right) \end{array}\right]\theta_{n} \end{array}$$

(A28b) $$\begin{array}{c} r_{i}=x_{i}-x_{0} \end{array}$$

(A28c) $$\begin{array}{c} {\bar{G_{sb}^{\prime }}}_{\theta_{n}}\theta_{n}=-Bd_{\theta_{n}} \end{array}$$

$$\begin{array}{c} {\bar{G_{sb}^{\prime }}}_{\theta_{n}}\theta_{n}=-\left[\begin{array}{cccc} B_{1} & B_{2} & B_{3} & B_{4} \end{array}\right]\left[\begin{array}{c} p_{1}\left(\hat{n}\times r_{1}\right)\\ p_{2}\left(\hat{n}\times r_{2}\right)\\ p_{3}\left(\hat{n}\times r_{3}\right)\\ p_{4}\left(\hat{n}\times r_{4}\right) \end{array}\right]\theta_{n}=-\sum_{i}^{N_{e}}p_{i}\left[B_{i}\left(\hat{n}\times r_{i}\right)\right]\theta_{n} \end{array}$$

(A28d) $$\begin{array}{c} {\bar{G_{sb}^{\prime }}}_{\theta_{n}}=-\sum_{i}^{N_{e}}p_{i}\left[B_{i}\left(\hat{n}\times r_{i}\right)\right] \end{array}$$

Noting that, if the system has been already worked in a local frame having the origin at the centroid of the fracture surface, nodal positions will be already the rotation radii with $r_{i}=x_{i}$. Again, ${\bar{G_{sb}^{\prime }}}_{\theta_{t}},{\bar{G_{sb}^{\prime }}}_{\theta_{m}}$ just follow:

(A29) $$\begin{array}{c} {\bar{G_{sb}^{\prime }}}_{\theta_{t}}=-\sum_{i}^{N_{e}}p_{i}\left[B_{i}\left(\hat{t}\times r_{i}\right)\right] \end{array}$$

(A30) $$\begin{array}{c} {\bar{G_{sb}^{\prime }}}_{\theta_{m}}=-\sum_{i}^{N_{e}}p_{i}\left[B_{i}\left(\hat{m}\times r_{i}\right)\right] \end{array}$$

Finally, for obtaining the displacement states $d_{k}$ for the case of simple axial strains, the most basic definition of the type ϵ=Δd/Δx→Δd=ϵΔx will be taken. This will consider, once again, the centroid of the fracture surface as the point where there are no displacements associated with this elastic strain. For instance, we will have the following for the case of $\epsilon_{n}$:

(A31) $$d_{\epsilon_{n}}=\left[\begin{array}{c} p_{1}\left(r_{1}\cdot \hat{n}\right)\epsilon_{n}\hat{n}\\ p_{2}\left(r_{2}\cdot \hat{n}\right)\epsilon_{n}\hat{n}\\ p_{3}\left(r_{3}\cdot \hat{n}\right)\epsilon_{n}\hat{n}\\ p_{4}\left(r_{4}\cdot \hat{n}\right)\epsilon_{n}\hat{n} \end{array}\right]=\left[\begin{array}{c} p_{1}r_{1n}\hat{n}\\ p_{2}r_{2n}\hat{n}\\ p_{3}r_{3n}\hat{n}\\ p_{4}r_{4n}\hat{n} \end{array}\right]\epsilon_{n}$$

As the simple axial strain $\epsilon_{n}$ is actually *meant to happen only* on the $\Omega^{+}$ domain, the linking equation for this mode will equal to a Heaviside function rather than to zero, making it imperative to use a domain-dependent ${\bar{G_{sb}^{\prime \pm }}}_{\epsilon_{n}}$:

$$\begin{array}{c} {\bar{G_{sb}^{\prime \pm }}}_{\epsilon_{n}}\epsilon_{n}+Bd_{\epsilon_{n}}=H_{\Gamma_{d}}\left[\begin{array}{c} \epsilon_{n}\\ 0\\ 0\\ 0\\ 0\\ 0 \end{array}\right]\Rightarrow {\bar{G_{sb}^{\prime \pm }}}_{\epsilon_{n}}\epsilon_{n}+\left[\begin{array}{cccc} B_{1} & B_{2} & B_{3} & B_{4} \end{array}\right]\left[\begin{array}{c} p_{1}r_{1n}\hat{n}\\ p_{2}r_{2n}\hat{n}\\ p_{3}r_{3n}\hat{n}\\ p_{4}r_{4n}\hat{n} \end{array}\right]\epsilon_{n}=\left[\begin{array}{c} H_{\Gamma_{d}}\\ 0\\ 0\\ 0\\ 0\\ 0 \end{array}\right]\epsilon_{n} \end{array}$$

(A32) $$\begin{array}{c} {\bar{G_{sb}^{\prime \pm }}}_{\epsilon_{n}}=\left[\begin{array}{c} H_{\Gamma_{d}}\\ 0\\ 0\\ 0\\ 0\\ 0 \end{array}\right]-\sum_{i}^{N_{e}}p_{i}r_{in}\left[B_{i}\hat{n}\right] \end{array}$$

${\bar{G_{sb}^{\prime \pm }}}_{\epsilon_{t}},{\bar{G_{sb}^{\prime \pm }}}_{\epsilon_{m}}$ will follow:

(A33) $$\begin{array}{c} {\bar{G_{sb}^{\prime \pm }}}_{\epsilon_{t}}=\left[\begin{array}{c} 0\\ H_{\Gamma_{d}}\\ 0\\ 0\\ 0\\ 0 \end{array}\right]-\sum_{i}^{N_{e}}p_{i}r_{it}\left[B_{i}\hat{t}\right],\;{\bar{G_{sb}^{\prime +}}}_{\epsilon_{m}}=\left[\begin{array}{c} 0\\ 0\\ H_{\Gamma_{d}}\\ 0\\ 0\\ 0 \end{array}\right]-\sum_{i}^{N_{e}}p_{i}r_{im}\left[B_{i}\hat{m}\right] \end{array}$$

With this, the right-hand expressions are covered, and all constraints concerning kinematic mode linking are closed and ready to be added to the α coefficients system. With the given compound definition for the φ functions, all populated terms of the left-hand side matrix operator $\bar{G_{sb}^{\prime +}}$ yield different expressions for the α coefficients. This adds 33 equations for each ± domain, which makes a total of 66 linear constraints.

We continue with the linear constraints belonging to the basic φ requirements of Equation (3). Considering the three direction-based, piece-wise definition of the φ functions proposed at the beginning of this section, the following constraints are added to the system:

(A34) $$\begin{array}{c} \varphi_{n}^{-}\left(x_{i}\right)=0,\;\varphi_{t}^{-}\left(x_{i}\right)=0,\;\varphi_{m}^{-}\left(x_{i}\right)=0\;x_{i}\in \Omega^{-}\\ \varphi_{n}^{+}\left(x_{i}\right)=1,\;\varphi_{t}^{+}\left(x_{i}\right)=1,\;\varphi_{m}^{+}\left(x_{i}\right)=1\;x_{i}\in \Omega^{+} \end{array}$$

This adds a total of 12 linear constraints.

The piece-wise definition for the φ functions requires constraints related to the $C^{0}$ continuity on the fracture surface $\Gamma_{d}$ as per Equation (70). If the φ functions are expressed on the local frame, it suffices just to set the coordinate ξ=0:

(A35) $${\left.\varphi_{j}^{+}\right|}_{\xi =0}={\left.\varphi_{j}^{-}\right|}_{\xi =0},$$

which implies that all $\alpha^{\pm }$ coefficients with not having the index 1 will impose a linear constraint. For the present work we have:

(A37) $$\begin{array}{cccccccc} \alpha_{j_{0}}^{+} & =\alpha_{j_{0}}^{-}, & \alpha_{j_{2}}^{+} & =\alpha_{j_{2}}^{-}, & \alpha_{j_{3}}^{+} & =\alpha_{j_{3}}^{-}, & \alpha_{j_{23}}^{+} & =\alpha_{j_{23}}^{-},\\ \alpha_{j_{22}}^{+} & =\alpha_{j_{22}}^{-}, & \alpha_{j_{33}}^{+} & =\alpha_{j_{33}}^{-}, & \alpha_{j_{223}}^{+} & =\alpha_{j_{223}}^{-}, & \alpha_{j_{233}}^{+} & =\alpha_{j_{233}}^{-},\\ \alpha_{j_{222}}^{+} & =\alpha_{j_{222}}^{-}, & \alpha_{j_{333}}^{+} & =\alpha_{j_{333}}^{-}, \end{array}$$

for j=n,t,m, making a total of 30 restrictions.

At last, we have the restrictions associated with the crack stiffness matrix M. For an enriched kinematic mode set and the assumption of a constant real stress field, the M matrix will have dimensions of 3×9. The first three columns of the matrix will certainly represent the same rigid body displacement coupling observed on the three crack displacement component approach, although the symmetry with respect to dot products observed in Equation (40) is not assured. At this point, one can choose to use the remaining free α coefficients to enforce some values in the crack stiffness matrix. More relations can be defined through the kinematic modes themselves, but this is left to the crack solution process (Section 5.4).

In this work, it has been decided to uncouple the rigid body displacements as in other approaches (out of diagonal components on the first three columns of M), as these are the most important kinematic modes of the framework. Six constraints are needed:

(A37) $$\begin{array}{c} M_{12}\left(\alpha \right)=0,\;M_{13}\left(\alpha \right)=0,\;M_{23}\left(\alpha \right)=0\\ M_{21}\left(\alpha \right)=0,\;M_{31}\left(\alpha \right)=0,\;M_{32}\left(\alpha \right)=0 \end{array}$$

For a compound φ definition, the diagonal terms lose the linear dependence once observed in Equation (40). If control is desired in all terms regulating crack rigid body displacement stiffness for terminal separation conditions, three more relations are required following the logic of Equation (59):

(A38) $$\begin{array}{cc} M_{11}\left(\alpha \right) & =-\frac{{\hat{n}}^{T}K_{e}d}{{\hat{n}}^{T}\left[\frac{F^{t}}{\sum p_{i}}-\frac{I_{3(4)}^{T}-F^{T}}{\sum \left(1-p_{i}\right)}\right]d}\\ M_{22}\left(\alpha \right) & =-\frac{{\hat{t}}^{T}K_{e}d}{{\hat{t}}^{T}\left[\frac{F^{t}}{\sum p_{i}}-\frac{I_{3(4)}^{T}-F^{T}}{\sum \left(1-p_{i}\right)}\right]d}\\ M_{33}\left(\alpha \right) & =-\frac{{\hat{m}}^{T}K_{e}d}{{\hat{m}}^{T}\left[\frac{F^{t}}{\sum p_{i}}-\frac{I_{3(4)}^{T}-F^{T}}{\sum \left(1-p_{i}\right)}\right]d} \end{array}$$

In the end, all linear relations imposed in terms of α coefficients add up to 117. The α coefficient set has up to 120 free parameters, which is theoretically enough to make the system solvable. Numerical handling of the linear system is discussed in Section 5.3.

## References

[1] Ibrahimbegovic A., Wilson E.L. (1991). A modified method of incompatible modes. Commun. Numer. Methods Eng..

[2] Oliver J., Caicedo M., Roubin E., Huespe A., Hernández J. (2015). Continuum approach to computational multiscale modeling of propagating fracture. Comput. Methods Appl. Mech. Eng..

[3] Moës N., Dolbow J., Belytschko T. (1999). A finite element method for crack growth without remeshing. Int. J. Numer. Methods Eng..

[4] Oliver J., Huespe A., Sánchez P. (2006). A comparative study on finite elements for capturing strong discontinuities: E-FEM vs. X-FEM. Comput. Methods Appl. Mech. Eng..

[5] Tabiei A., Zhang W. (2016). Evaluation of various numerical methods in LS-DYNA® for 3D Crack Propagation. Proceedings of the Conference Proceedings 14th International LS-DYNA Users Conference.

[6] Shi J., Lua J., Chen L., Chopp D., Sukumar N. (2009). X-FEM for Abaqus (XFA) Toolkit for Automated Crack Onset and Growth Simulation: New Development, Validation, and Demonstration. Proceedings of the Conference Proceedings 2009 SIMULIA Customer Conference.

[7] Weaver C.M., Rigg P.A., Cordes J.A., Haynes A. XFEM Analyses of Critical Cracks in a Pressure Tap for a 40mm Gun Breech. Proceedings of the Conference proceedings 2011 SIMULIA Customer Conference.

[8] Contrafatto L., Cuomo M., Tommaso G., Venti D. Computational issues in the Finite Element with Embedded Discontinuity Method based on non-homogenous displacement jump. Proceedings of the Conference proceedings Aimeta XXI.

[9] Wells G.N. (2001). Discontinuous Modelling of Strain Localisation and Failure. Ph.D. Thesis.

[10] Oliver J. (1996). Modelling strong discontinuities in solid mechanics via strain softening constitutive equations. Part 2: Numerical simulation. Int. J. Numer. Methods Eng..

[11] Linder C., Armero F. (2007). Finite elements with embedded strong discontinuities for the modeling of failure in solids. Int. J. Numer. Methods Eng..

[12] Da Costa D.D., Alfaiate J., Sluys L., Júlio E. (2009). A discrete strong discontinuity approach. Eng. Fract. Mech..

[13] Dujc J., Brank B., Ibrahimbegovic A., Brancherie D. (2010). An embedded crack model for failure analysis of concrete solids. Comput. Concr..

[14] Jirásek M. (2000). Comparative study on finite elements with embedded cracks. Comput. Methods Appl. Mech. Eng..

[15] Oliver J. (1996). Modelling strong discontinuities in solid mechanics via strain softening constitutive equations. Part 1: Fundamentals. Int. J. Numer. Methods Eng..

[16] Dvorkin E.N., Cuitiño A.M., Gioia G. (1990). Finite elements with displacement interpolated embedded localization lines insensitive to mesh size and distortions. Int. J. Numer Methods Eng..

[17] Simo J.C., Oliver J., Armero F. (1993). An analysis of strong discontinuities induced by strain-softening in rate-independent inelastic solids. Comput. Mech..

[18] Armero F., Garikipati K. (1996). An analysis of strong discontinuities in multiplicative finite strain plasticity and their relation with the numerical simulation of strain localization in solids. Int. J. Solids Struct..

[19] Oliver J., Huespe A.E., Blanco S., Hirnyj S. (2005). On a finite element with embedded discontinuities for numerical modeling of fracture. Proceedings of the 11th International Conference on Fracture ICF11.

[20] Simo J.C., Rifai M.S. (1990). A class of mixed assumed strain methods and the method of incompatible modes. Int. J. Numer. Methods Eng..

[21] Wells G., Sluys L. (2001). Three-dimensional embedded discontinuity model for brittle fracture. Int. J. Solids Struct..

[22] Roubin E., Vallade A., Benkemoun N., Colliat J.B. (2015). Multi-scale failure of heterogeneous materials: A double kinematics enhancement for Embedded Finite Element Method. Int. J. Solids Struct..

[23] Hauseux P., Roubin E., Colliat J.B., Shojaei A.K., Shao J. (2017). The embedded finite element method (E-FEM) for multicracking of quasi-brittle materials. Porous Rock Fracture Mechanics.

[24] Stamati O., Roubin E., Andò E., Malecot Y. (2019). Tensile failure of micro-concrete: From mechanical tests to FE meso-model with the help of X-ray tomography. Meccanica.

[25] Stamati O. (2020). Impact of Meso-Scale Heterogeneities on the Mechanical Behaviour of Concrete: Insights from In-Situ X-ray Tomography and E-FEM Modelling. Ph.D. Thesis.

[26] Alfaiate J., Simone A., Sluys L. (2003). Non-homogeneous displacement jumps in strong embedded discontinuities. Int. J. Solids Struct..

[27] Dujc J., Brank B., Ibrahimbegovic A. (2010). Quadrilateral finite element with embedded strong discontinuity for failure analysis of solids. Comput. Model. Eng. Sci..

[28] Raina A., Linder C. (2010). Modeling crack micro-branching using finite elements with embedded strong discontinuities. PAMM.

[29] Dias-da Costa D., Alfaiate J., Sluys L., Areias P., Júlio E. (2013). An embedded formulation with conforming finite elements to capture strong discontinuities. Int. J. Numer. Methods Eng..

[30] Brunton S., Kutz J. (2019). Data-Driven Science and Engineering—Machine Learning, Dynamical Systems, and Control.

[31] The Sage Developers (2019). SageMath, the Sage Mathematics Software System (Version 8.7). https://www.sagemath.org.

[32] Kluyver T., Ragan-Kelley B., Pérez F., Granger B., Bussonnier M., Frederic J., Kelley K., Hamrick J., Grout J., Corlay S., Loizides F., Scmidt B. (2016). Jupyter Notebooks—A publishing format for reproducible computational workflows. Positioning and Power in Academic Publishing: Players, Agents and Agendas.

[33] Hauseux P. (2015). Propagation D’Incertitudes Paramétriques Dans Les Modèles Numériques en Mécanique Non Linéaire: Applications à des Problèmes D’excavation. Ph.D. Thesis.

[34] Vallade A. (2016). Modélisation Multi-échelles des Shales: Influence de la Microstructure sur les Propriétés Macroscopiques et le Processus de Fracturation. Ph.D. Thesis.

